# Isolation of the Lanostane Triterpenes Pholiols L–S from *Pholiota populnea* and Evaluation of Their Antiproliferative and Cytotoxic Activities

**DOI:** 10.3390/ph16010104

**Published:** 2023-01-10

**Authors:** Morteza Yazdani, Anita Barta, Anasztázia Hetényi, Róbert Berkecz, Gabriella Spengler, Attila Ványolós, Judit Hohmann

**Affiliations:** 1 Institute of Pharmacognosy, University of Szeged, 6720 Szeged, Hungary; 2 Department of Medical Chemistry, University of Szeged, 6720 Szeged, Hungary; 3 Institute of Pharmaceutical Analysis, University of Szeged, 6720 Szeged, Hungary; 4 Department of Medical Microbiology, Albert Szent-Györgyi Health Center and Albert Szent-Györgyi Medical School, University of Szeged, 6725 Szeged, Hungary; 5 Department of Pharmacognosy, Semmelweis University, 1085 Budapest, Hungary; 6 Interdisciplinary Centre for Natural Products, University of Szeged, 6720 Szeged, Hungary; 7 ELKH-USZ Biologically Active Natural Products Research Group, University of Szeged, 6720 Szeged, Hungary

**Keywords:** *Pholiota populnea*, strophariaceae, antiproliferative activity, cytotoxic activity, lanostane, pholiol

## Abstract

Pholiols L-S (**1**–**8**), eight undescribed triterpenes were isolated from the sporocarps of the mushroom *Pholiota populnea*. Various chromatographic techniques, such as open column chromatography, flash chromatography, gel filtration, preparative thin layer chromatography, and HPLC, were applied to purify the compounds. The structure elucidation was carried out by spectroscopic analysis, including 1D (^1^H NMR and ^13^C JMOD) and 2D NMR (^1^H-^1^H COSY, HSQC, HMBC and NOESY) and HRESIMS experiments. The isolated compounds had lanostane (**1**–**7**) or trinorlanostane (**8**) skeletons; all of them were substituted with 3-hydroxy-3-methylglutaroyl group or its 6-methyl ester. Five compounds (**1**, **2**, **4**, **6**, and **8**) were investigated for their antiproliferative and cytotoxic activity in vitro by MTT assay on breast cancer (MCF-7), human colon adenocarcinoma (sensitive Colo 205, and resistant Colo 320), non-small cell lung cancer (A549), and human embryonic lung fibroblast (MRC-5) cell lines. Pholiols M (**2**) and O (**4**) showed antiproliferative activity against the MCF-7 cell line with IC_50_ of 2.48 and 9.95 µM, respectively. These compounds displayed tumor cell selectivity on MCF-7 cells with SI values of >40 (**2**) and 4.3 (**4**), but they did not show a cytotoxic effect, proving their action exclusively on tumor cell proliferation. Pholiols L (**1**) and Q (**8**) were found to have selective cytotoxicity on drug resistant cells in comparison to their effects on Colo320 and Colo205 cells [relative resistance values 0.84 (**1**) and 0.62 (**8**)].

## 1. Introduction

For many decades, mushrooms have been utilized because of their beneficial nutritional and medicinal values, including immunomodulatory and antitumor activities. Fungal metabolites can have a protective effect by stimulating the immune response and activating immune cells, macrophages, T cells, and natural killer cells. Furthermore, bioactive compounds of mushrooms have shown cytotoxicity, antiproliferative activity, apoptosis-inducing properties, and have been shown to act as cancer cell metastases inhibitory agents. Certain dietary mushrooms (e.g., *Agaricus bisporus*) were reported to contain anticarcinogenic metabolites which alter the aromatase enzyme activity. The active ingredients responsible for the immunomodulatory and antitumor activities of mushrooms include polysaccharides, polysaccharide-protein complexes, lectins, amino acids, polyphenols, sterols, and other terpenoids [1,2,3].

Polysaccharides from *Pholiota adiposa*, especially SPAP2-1, showed strong interference of the cell cycle of HeLa cells and induced apoptosis [4]. A protein purified from the edible fungus *Pholiota nameko* (PNAP) shows promise for the treatment of breast cancer, as it induced apoptosis of human breast adenocarcinoma MCF-7 cells in vivo and modulated cytokine secretion in mice bearing MCF-7 xenografts [5]. The glucan polysaccharides schizophyllan, derived from *Schizophyllum commune*, and lentinan, produced by *Lentinula edodes*, were investigated in clinical trials, and a better overall survival of patients with head and neck cancer and gastric and colorectal carcinomas, respectively, were found [6].

*Ganoderma lucidum* polysaccharides and triterpenoids were found to be potent inhibitors of tumor growth in vitro and in vivo [7]. Moreover, extracts of *G. lucidum* and *G. tsugae* were able to inhibit the growth of colorectal cancer cells in vitro [8]. Ergosterol, the main sterol of mushroom species, is able to induce apoptosis in leukemia cells and inhibits tumor-induced angiogenesis [9]. *Hypholoma lateritium* (Strophariaceae) was reported to contain lanostane-type triterpenoids, sublateriols A–C, and fasciculol A–C, some of them exhibited cytotoxicity against human cancer cell lines (A549, SK-OV-3, SK-MEL-2, and HCT-15) in vitro [10].

In our previous study, *Pholiota populnea* was investigated for antitumor compounds, and lanostane diesters, named pholiols A–D, ergosterol, 3*β*-hydroxyergosta-7,22-diene, and a polyhydroxy-squalene derivative were isolated. Ergosterol was found to show cytotoxic activity against sensitive (Colo 205) and resistant colon adenocarcinoma cells (Colo 320) with IC_50_ values of 4.9 and 6.5 μM, respectively. The *p*-glycoprotein (ABCB1) efflux pump inhibitory activity of the compounds was also tested against resistant Colo 320 cells and inhibitory activity was found for pholiols A and B and the squalene derivative. In addition, the drug interactions of triterpenes with doxorubicin were also studied by the checkerboard method on Colo 320 cells, and it was observed that pholiols B and D interacted in a synergistic manner, while the acyclic triterpene acted in a very strong synergistic manner [11]. Further mycochemical investigations led to the isolation of pholiols E-K and (+)-clavaric acid, some of the triterpenes exerted moderate COX-2 and 5-LOX inhibitory activities. Pholiol F (IC_50_ 194.5 μM against 5-LOX, and 439.8 μM against COX-2) was found to be the most effective among the investigated compounds [12].

In continuation of our study on *P. populnea*, we herein report the isolation of eight undescribed triterpenes, namely, pholiols L–S (**1**–**8**) from the chloroform and ethyl acetate extracts of the mushroom, and investigation of the cytotoxic and antiproliferative activity of the compounds.

## 2. Results and Discussion 

### 2.1. Structure Determination of Compounds 1−8

**Figure 1:**
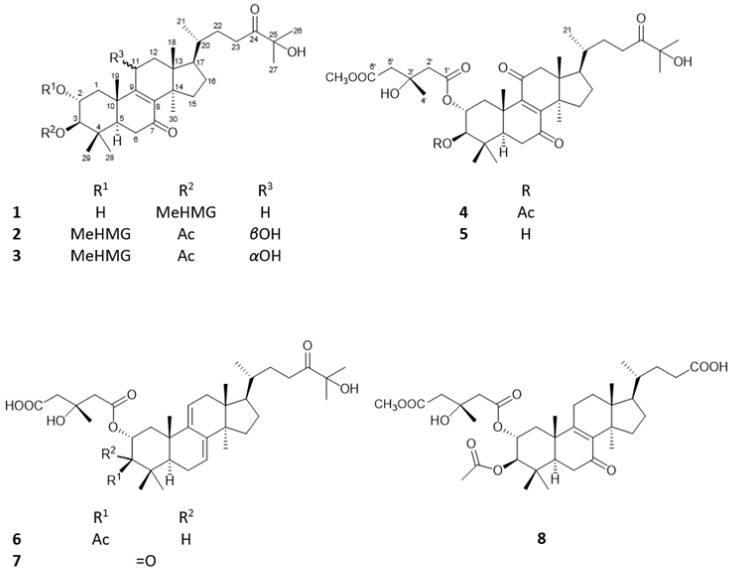
Pholiols L-S (**1**–**8**) isolated from *Pholiota populnea.* MeHMG = 3-hydroxy-3-methyl-glutaryl 6-methyl ester.

Eight triterpenes (**1**–**8**) were isolated from the chloroform- and ethyl acetate–soluble phase of the MeOH extract obtained from *P. populnea* by a combination of different chromatographic methods, such as open column chromatography (OCC), flash chromatography (FC), gel filtration (GF), preparative thin layer chromatography (TLC), and high-performance liquid chromatography (HPLC). The structure elucidation was carried out by spectroscopic analysis, including 1D and 2D NMR [¹H-¹H Correlation Spectroscopy (^1^H-^1^H COSY), Heteronuclear Single Quantum Coherence (HSQC), Heteronuclear Multiple Bond Correlation (HMBC), and Nuclear Overhauser Effect Spectroscopy (NOESY)] and HRESIMS experiments. The NMR and MS data showed that all compounds were lanostane (**1**–**7**) or trinorlanostane esters (**8**) (Figure 1).

**Figure 2:**
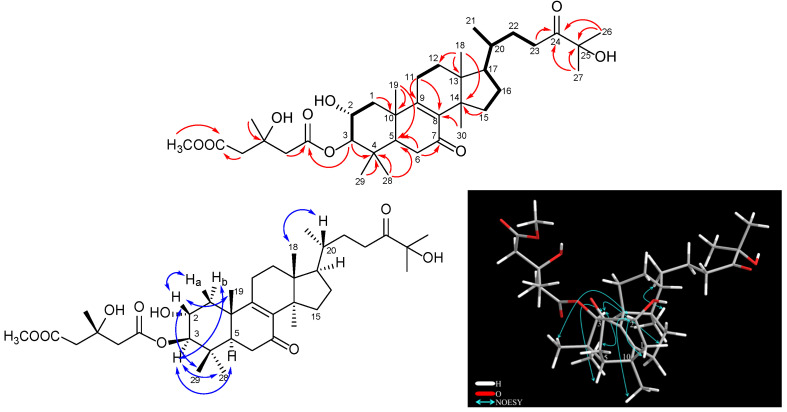
Key COSY (–), HMBC (→), and NOESY (H↔H) correlations of pholiol L (**1**).

Compound **1**, named pholiol L, was obtained as a colorless amorphous solid with [*α*]^23^_D_ −11.6 (*c* 0.1, CHCl_3_). Its HRESIMS spectrum exhibited a protonated molecular ion at *m*/*z* 647.4147 [M + H]^+^ (calcd for C_37_H_59_O_9_^+^ 647.4154), indicating the molecular formula of C_37_H_58_O_9_. The ^1^H NMR and ^13^C-JMOD spectra indicated a 3-hydroxy-3-methylglutaric acid 6-methyl ester (MeHMG) derivative [*δ*_H_ 2.75 m, 2.57 m (H_2_-2′), 2.92 m, 2.27 m (H_2_-5′), 1.42 s (H_3_-4′), 3.72 s (OCH_3_-7′); *δ*_C_ 171.41 (1′), 173.26 (C-6′), 46.33 (C-2′), 43.68 (C-5′), 70.37 (C-3′), 28.29 (C-4′), 52.05 (OCH_3_-7′)], substituted with two keto groups (*δ*_C_ 198.29 and 215.01); the presence of two hydroxy groups was suggested by the carbon resonances of a methine group at *δ*_C_ 66.89 (C-2) and a quaternary carbon at *δ*_C_ 76.34 (C-25). A sequence of the correlated protons in the ^1^H–^1^H COSY spectrum –CH_2_–CH(OR)–CH(OR)– (*δ*_H_ 2.21 dd (12.5, 3.8 Hz), 1.44 m, 3.92 dt (3.8, 11.1 Hz), 4.60 d (9.9 Hz) could be assigned to the C-1–C-3 part of the molecule, with regard to the HMBC correlations between H_2_-1 and C-10; H_3_-19 and C-10; H-3 and C-4; H_3_-28 and H_3_-29 and C-4; H-1a, H_3_-28, H_3_-29, and H_3_-19 with C-5 (Figure 2). NMR data of compound **1** were very similar to those of the previously isolated pholiol H, with the major differences in chemical shifts of H-2, H-3 (**1**: *δ*_H-2_ 3.92 dt, *δ*_H-3_ 4.60 d versus pholiol H: *δ*_H-2_ 5.07 dt, *δ*_H-3_ 3.24 d), and C-2 and C-3 (**1**: *δ*_C-2_ 66.89, *δ*_C-3_ 84.53 versus pholiol H: *δ*_C-2_ 73.41, *δ*_C-3_ 79.99) [12] demonstrating the connection of the ester group at position C-3 and hydroxyl group at C-2 in **1**. The relative configuration of **1** was determined based on the Overhauser effects detected in the NOESY spectrum. The NOESY correlation of H-3 (*δ*_H_ 4.60) with H-1*α* (*δ*_H_ 1.44), H_3_-28 (*δ*_H_ 0.91), and H-5 (*δ*_H_ 1.82) showed these protons in the *α* orientation, while correlations of H-2 (*δ*_H_ 3.92) with H-1*β* (*δ*_H_ 2.21), H_3_-19 (*δ*_H_ 1.24), and H_3_-29 (*δ*_H_ 0.96) indicated the *β* orientation of H-2 and the 19- and 29-methyl groups (Figure 2). The Overhauser effect between H_3_-18 and H-20 was indicative for their *β* position, similar to the previously isolated pholiol compounds [11,12]. All of the above evidence confirmed the structure of pholiol L (**1**), as depicted in Figure 1.

Compound **2** (pholiol M) was obtained as a colorless amorphous solid with an optical rotation of [*α*]^23^_D_ −10.9 (*c* 0.05, CHCl_3_). Its molecular formula was assigned as C_39_H_60_O_11_ by the deprotonated molecular ion at *m*/*z* 703.4078 [M-H]^−^ (calcd for C_39_H_59_O_11_^−^ 703.4063) in HRESIMS. The ^1^H NMR and ^13^C-JMOD spectrum showed that compound **2** is a lanostane diester esterified with an acetic acid [*δ*_H_ 2.07 s (3H); *δ*_C_ 170.84 (CO), 21.07 (CH_3_)], and a 3-hydroxy-3-methylglutaryl 6-methyl ester [*δ*_H_ 2.68 m, 2.54 m (H_2_-2′), 2.70 m, 2.62 m (H_2_-5′), 1.35 s (H_3_-4′), 3.71 s (OCH_3_-7′); *δ*_C_ 171.44 (1′), 172.28 (C-6′), 44.99 (C-2′), 45.04 (C-5′), 69.72 (C-3′), 27.70 (C-4′), 51.94 (OCH_3_-7′)] (Table 1 and Table 2). Similarly to pholiol L (**1**), compound **2** contained the 8,9-en-7-one moiety [*δ*_C_ 139.69 (C-8), 160.48 (C-9), 199.71 (C-7)] and C_8_ aliphatic chain at C-17 substituted with a keto [*δ*_C_ 214.92 (C-24)] and a hydroxy group [*δ*_C_ 76.38 (C-25)]. The ^1^H NMR and JMOD spectra exhibited an additional *O*-substituted methine at *δ*_H_ 4.57 br t (5.7 Hz), *δ*_C_ 67.31, which was placed at C-11 with regard to the ^1^H-^1^H COSY [*δ*_H_ 2.28 m, 1.88 m, 4.57 br t; –CH_2_–CH(OH)–; C-12–C-11] and HMBC correlations of H-11 with C-8 and C-13; and H-12 with C-14. The assignment of C-11 (*δ*_C_ 67.31) and C-12 (*δ*_C_ 42.90) were in agreement with literature data [13]. The ^1^H-^1^H COSY spectrum defined the structural fragment with correlated protons –CH_2_–CH(OR)–CH(OR)– (*δ*_H_ 2.87 dd, 1.70 t, 5.29 dt, and 4.83 d) (C-1–C-3). The positions of the ester groups were established via the HMBC experiment. The correlations of the carbonyl signals at *δ*_C_ 171.44 (1′) and 170.84 (AcCO) with the proton signals at *δ*_H_ 5.29 (H-2) and *δ*_H_ 4.83 (H-3) indicated the presence of the MeHMG and acetyl groups at C-2 and C-3, respectively. The relative configuration was determined with the use of a NOESY measurement. The Overhauser effects between H-5/H-3, H-3/H_3_-28, H-3/H-1*α*, and H-1*α*/H-11 pointed these protons in the *α* position, while those between H-1*β*/H-2, H-2/H_3_-19, H_3_-19/H-1*β*, and H-2/H_3_-29 indicated their *β* position. The above findings were consistent with the proposed structure of **1**, as depicted in Figure 1.

Compound **3**, named pholiol *n*, was isolated as a white amorphous powder with an optical rotation of [*α*]^23^_D_ −8.9 (*c* 0.05, CHCl_3_). Its molecular composition was determined as C_39_H_60_O_11_ based on the sodiated molecular ion peak at *m*/*z* 727.4041 [M + Na]^+^ (calcd for C_39_H_60_O_11_Na^+^ 727.4028) detected in the HRESIMS spectrum. Careful evaluation of the 1D and 2D NMR spectra of **3** resulted in the elucidation of the same planar structure as that of compound **2**. The main differences were found in the chemical shift values of carbons C-11–C-12 [*δ*_C_ 65.55 (C-11), 44.40 (C-12) for **3**; *δ*_C_ 67.31 (C-11), 42.90 (C-12) for **2**], indicating that the orientation of 11-OH group may be different. This was confirmed by the NOESY correlations of **3**; the Overhauser effects between H_3_-18 (*δ*_H_ 0.65 s) and H-12*β* (*δ*_H_ 2.46 m); H-12*β* and H-11 (*δ*_H_ 4.49 m); H-12*α* (*δ*_H_ 1.84 m) and H_3_-30 (*δ*_H_ 1.12 s) proved the *α* position of the 11-hydroxy group. NOEs of H-2 with H_3_-19 and H_3_-28; and that of H-3 with H_3_-29; H_3_-18 with H-20 corroborated the stereostructure of compound **3**, as depicted in Figure 1.

Compound **4**, named pholiol O, was obtained as a colorless amorphous solid with an optical rotation of [*α*]^27^_D_ +11.8 (*c* 0.05, CHCl_3_). It gave the molecular formula C_39_H_58_O_11_, determined from the HRESIMS by the sodiated molecular ion peak at *m*/*z* 725.3877 (M + Na)^+^, (calcd for C_39_H_58_O_11_Na^+^ 725.3871). Analysis of ^1^H NMR and ^13^C-JMOD spectra of **4** indicated the presence of the same esterification pattern and aliphatic chain at C-17 as in compound **2** (Table 1 and Table 2). The main differences were the remarkable difference in chemical shifts of C-8 (**2**: *δ*_C_ 139.69; **4**: *δ*_C_ 152.20), and C-9 (**2**: *δ*_C_ 160.48; **4**: *δ*_C_ 151.87), and the presence of an additional keto group (*δ*_C_ 203.60) at C-11 in compound **4**. The position of this keto group was established based on its HMBC correlations with H_2_-12 [*δ*_H_ 2.87 d, 2.58 d (*J* = 16.1 Hz)]. NOESY correlations between H-3 and H-5, and H_3_-28, H-1*α*; between H-2 and H-1*β*, and H_3_-19; between H-12*β* and H_3_-18; between H-12*α* and H-30; and between H-30 and H-20 allowed the stereochemical features identical with those of compounds **1** and **2** to be assigned for **4** (Figure 1).

Compound **5** (pholiol *p*) was obtained as a white amorphous solid with an optical rotation of [*α*]^23^ −1.6 (*c* 0.05, MeOH). Its molecular formula was deduced to be C_39_H_56_O_10_ from HRESIMS sodium adduct ion peak at *m*/*z* 683.3767 [M + Na]^+^ (calcd for C_37_H_56_O_10_Na^+^ 683.3766). Comprehensive analysis of ^1^H NMR, ^13^C JMOD and 2D NMR data of **4** and **5** indicated the presence of the same 2,3,25-trihydroxy-7,11,24-triketo-triterpene core and MeHMG esterification at C-2 as discussed at compound **4**, with the exception of the acetoxy group at C-3, which was replaced by a hydroxy group. This was clearly shown by the H-3 signal at *δ*_H_ 3.28 d (*J* = 11.0 Hz) for **5**, instead of H-3 at *δ*_H_ 3.28 d (*J* = 11.0 Hz) for **4** (Table 1 and Table 2). NOESY correlations between H-2/H_3_-19, H-3/H_3_-28, H-11*α*/H_3_-30, and H_3_-28/H-6*α* permitted the same stereochemistry of **5** as that of **4**.

Compound **6**, pholiol Q, was isolated as a colorless amorphous solid with an optical rotation of [*α*]^27^_D_ +5.0 (*c* 0.1, MeOH). Its molecular formula was determined to be C_38_H_58_O_9_ based on the HRESIMS ion peak at *m*/*z* 657.4010 [M–H]^−^ (calcd for C_38_H_57_O_9_^−^, 657.4008). Analysis of ^1^H NMR and ^13^C–JMOD as well as 2D NMR data demonstrated the presence of a 3-hydroxy-3-methyl glutarate and an acetate group in **6**, and the same side chain at C-17 as in pholiols L–N (**1**–**5**) (Table 1 and Table 2). NMR signals of **6** at *δ*_H_ 5.53 t (H-7) and 5.39 d (H-11), and *δ*_C_ 121.19 (C-7), 143.87 (C-8), 146.28 (C-9), 118.31 (C-11) suggested two trisubstituted olefin groups which were placed at C-7–C-8, and C-9–C-11 considering the HMBC correlations of C-9 with H-7, H-12b, H-1b, H_3_-19, and those of C-8 with H-11, H-6, and H_3_-30. NOESY cross-peaks between H-2/H_3_-29, H-2/H-1*β*, H-1*β*/H_3_-19, H-3/H-1*α*, and H_3_-30/H-17 afforded the same stereochemical assignment of compound **6** as that of **1**–**5**.

Compound **7**, pholiol R, was isolated as a white amorphous solid with an optical rotation of [*α*]^22^_D_ +24.9 (*c* 0.05, MeOH). Its HRESIMS spectrum exhibited a molecular ion peak at *m*/*z* 613.3768 [M–H]^−^ (calcd for C_36_H_53_O_8_^−^, 613.3746) indicating the molecular formula of C_36_H_54_O_8_. ^1^H and ^13^C NMR assignments of **7**, prepared by analysis of the ^1^H-^1^H COSY, HSQC and HMBC spectra, showed that chemical shifts of **7** were very similar to that of **6** differing only in the resonances of ring A protons and carbons (Table 1 and Table 2). In case of **7** a carbonyl group (*δ*_C_ 210.63) can be found at position C-3, as it was confirmed by the HMBC correlation between C-3 and H_3_-28 and H_3_-29. Cross-peaks in the NOESY spectrum of **7** between H-2/H_3_-29, H-2/H_3_-19, H-20/H_3_-18, H_3_-30/H-17, H-5/H_3_-28, H-5/H-6*α*, and H-6*β*/H-19 were in agreement with the stereostructure of **7**, as depicted in Figure 1.

Compound **8** (pholiol S) was a colorless amorphous solid and had an optical rotation of [*α*]^23^_D_ −14.2 (*c* 0.1, CHCl_3_). Its HRESIMS spectrum exhibited sodium adduct ion peak at 669.3623 [M + H]^+^ (calcd for C_36_H_54_O_10_Na^+^ 669.3609), indicating the molecular formula of C_36_H_54_O_10_. The ^1^H NMR and ^13^C JMOD spectra evidenced the presence of one acetate and one 3-hydroxy-3-methylglutaryl 6-methyl ester group in the molecule, and a 27 carbon atom containing basic skeleton (Table 1 and Table 2). The chemical shifts of skeletal protons and carbons were very similar to those of pholiol B [11], with exception of the resonances of the C-17 side chain. The ^1^H-^1^H COSY spectrum of **8** indicated a structural fragment with correlated protons at *δ*_H_ 1.43 m, 0.92 d (3H), 1.85 m, 1.35 m, 2.40 m, 2.27 m [–CH(CH_3_)–CH_2_–CH_2_–] which was assigned as C-20–C-23 part of **8**. This structural fragment was coupled with a carboxyl group (*δ*_C_ 178.71, C-24) with help of its HMBC correlations with H_2_-22 (*δ*_H_ 1.85 m), and H_2_-23 (*δ*_H_ 2.40 m, 2.27 m), and connected to C-17 as showed by the long-range correlation of C-17 (*δ*_C_ 48.94) with H_3_-21 (*δ*_H_ 0.92 d). NOESY correlations between H-2/H_3_-29, H-2/H_3_-19, H-2/H-1*β*, H-3/H_3_-28, H-3/H-1*α*, H_3_-19/H-1*β*, and H_3_-18/H-20 proved the stereochemistry of compound **8**, as shown in Figure 1.

### 2.2. Evaluation of the Antiproliferative and Cytotoxic Activity of the Isolated Compounds

The compounds isolated in good yield, such pholiols L, M, O, Q, and S (**1**, **2**, **4**, **6**, and **8**), were investigated for their antiproliferative activity in vitro by MTT assay in human colon adenocarcinoma cell lines, both sensitive (Colo 205) and resistant (Colo 320) ones, hormone-responsive breast cancer (MCF-7), human non-small cell lung cancer (A549), and human embryonic lung fibroblast cell line (MRC-5). The anticancer drug doxorubicin was used as a reference agent. The results, obtained as the concentration of the compound that produced half of the inhibition (IC_50_), are shown in Table 3. The highest antiproliferative effects were observed for pholiols M (**2**) and O (**4**) against the MCF-7 cell line, with IC_50_ values of 2.48 and 9.95 µM, respectively. These compounds were also potent against Colo 205 cells, but to a lower extent [IC_50_ 23.91 (**2**), and 23.30 µM (**4**)], while pholiol L (**1**) was moderately active on the MRC-7 cell line (IC_50_ 21.74 µM). Structurally, compounds **2** and **4** are lanostane triterpenes with 2,3-diester-7-on-8 (9)-en functionalities, with a further 11-keto (**4**) or 12-hydroxy group (**2**). All other compounds exhibited weaker activity on the viability of the treated cancer cells, displaying IC_50_ values ranging from 28.07 to 89.84 µM. Compounds **1**, **2**, **4**, **6**, and **8** were tested for their antiproliferative activity in the non-cancerous human embryonic lung fibroblast cell line as well, and on the basis of these results, selectivity indexes (SI) were calculated. The SI values of pholiol M (**2**) > 40 and 4.18 indicated its strong and moderate tumor cell selectivity regarding the MCF-7 and Colo 205 cell lines, respectively, whereas SI values of 4.3 and 1.8 of pholiol O (**4**) indicated this compound to be moderately or slightly selective towards Colo 205 cells [14]. Compounds **1**, **6**, and **8** did not display selectivity to cancerous cell lines over normal cells.

The direct cytotoxic effects of the compounds were determined similarly by MTT assay on the Colo 205, Colo 320, MCF-7, A549, and MRC-5 cell lines, using a higher cell population and a shorter exposure than in the antiproliferative test. Under these conditions, the direct killing effect can be measured instead of the cell growth inhibitory activity. Significant difference between the antiproliferative and cytotoxic effects displayed selectivity for the growing cell population without directly killing the exposed cells. Such a result was exhibited by pholiols M (**2**) and O (**4**), for which, no cytotoxic effect could be detected on MCF-7 cells (IC_50_ > 100 µM), proving their action exclusively on tumor cell proliferation. Considering all other compounds and cell lines, moderate cytotoxic activities were measured with IC_50_ from 31.52 to over 100 µM (Table 4).

Comparison of the cytotoxic activities on drug-resistant (Colo 320) and sensitive (Colo 205) cells allowed for the detection of relative resistance (RR), which was calculated as the ratio of the IC_50_ value in the resistant and in sensitive cancer cell lines. Compounds with RR < 1 show selectivity against resistant cells, whereas RR ≤ 0.5 means that the compounds have a collateral sensitivity (CS) effect [15]. The calculated RR values of the tested compounds showed selectivity against the resistant Colo 320 cells of pholiols L (**1**) (RR 0.84) and Q (**8**) (RR 0.62) (Table 4).

## 3. Materials and Methods

### 3.1. General Experimental Procedures

The optical rotations were determined using a JASCO *p*-2000 polarimeter (JASCO International, Co., Ltd., Hachioji, Tokyo, Japan). High-resolution mass spectrometry (HRMS) was performed on a Thermo Velos Pro Orbitrap Elite (Thermo Fisher Scientific, Bremen, Germany) instrument, using electron spray ionization (ESI) method, operating in the positive or negative ion mode. The (de)protonated molecular ion peaks were fragmented by the collision-induced dissociation method (CID) at a normalized collision energy of 35%. Helium was used as a collision gas in the CID experiments. The data were acquired and processed with MassLynx software. NMR spectra were recorded in CDCl_3_ or CD_3_OD on a Bruker Avance DRX 500 spectrometer (Bruker, Billerica, MA, USA) at 500 MHz (^1^H) and 125 MHz (^13^C). The signals of the deuterated solvents were taken as references. Two-dimensional (2D) NMR measurements were carried out with standard Bruker software. In the COSY, HSQC, and HMBC experiments, gradient-enhanced versions were applied. Flash chromatography (FC) was performed on a CombiFlash Rf+ Lumen instrument with integrated UV, UV–Vis, and ELS detection using normal phase (silica 60, 0.045–0.063 mm, Molar Chemicals, and RediSep Rf Gold, Teledyne Isco, Lincoln NE, USA) flash column. Sephadex LH-20 (25–100 μm, Sigma-Aldrich, MO, USA) was employed for gel filtration (GF). Reversed-phase HPLC (RP-HPLC) and normal-phase HPLC (NP-HPLC) separations were carried out on a Shimadzu LC-10 A S HPLC instrument equipped with a UV–Vis detector (Shimadzu, Co., Ltd., Kyoto, Japan) over reversed-phase (RP-HPLC, LiChrospher RP-18, 5 μm, 250 × 4 mm) and normal-phase (NP-HPLC, LiChrospher Si60, 5 μm, 250 × 4 mm) columns, respectively. Preparative thin-layer chromatography (Prep TLC) was conducted using silica plates (20 × 20 cm silica gel 60 F_254_, Merck 105,554). Sigma-Aldrich Kft. (Budapest, Hungary) and Molar Chemicals (Halásztelek, Hungary) provided the chemicals used in this research.

### 3.2. Mushroom Material

Sporocarps of *Pholiota populnea* (Pers.) Kuyper & Tjall–Beuk. (*Strophariaceae*) were gathered in the autumn of 2017 near Szeged, Hungary (46.400556, 20.190556). The samples were identified by Attila Sándor (Mushroom Society of Szeged, Hungary) and stored at −20 °C until processing. A voucher specimen was deposited at the Institute of Pharmacognosy, University of Szeged, Szeged, Hungary (No. H019).

### 3.3. Extraction and Isolation

The fresh mushroom material (4.2 kg) was ground in a blender and then percolated with MeOH (20 L) at room temperature. After concentration, the dry residue (151 g) was dissolved in 600 mL of 50% aqueous MeOH and subjected to solvent–solvent partition with *n*-hexane (5 × 500 mL), CHCl_3_ (5 × 500 mL), and then EtOAc (5 × 500 mL). The chloroform-soluble phase (7 g) was subjected to gel filtration (GF) on Sephadex LH-20, using dichloromethane–methanol (1:1) as a mobile phase, providing 10 fractions (F1-10). The combined fractions F5 (780 mg) and F6 (320 mg) were further separated by flash chromatography (NP-FC) on silica gel (12 g) using an *n*-hexane–acetone gradient system (linear from 0% to 100% acetone, t = 65 min), and at the end, MeOH for 8 min. Fractions with similar compositions were combined based on TLC monitoring (G1-21). Fraction G12 (161 mg) was separated by RP-HPLC with H_2_O- MeCN (25:75) as mobile phase, which resulted in 5 fractions (K1-5). Purification of fraction K2 (54.6 mg) was conducted by NP-HPLC using a cyclohexane–ethyl acetate (25:75) solvent system to isolate compounds **1** (8.2 mg), **8** (8.3 mg), and to obtain 3 other subfractions (L1-3). Subfraction L1 (5.6 mg) was purified by NP-HPLC (mobile phase: cyclohexane–EtOAc, 15:50) to yield compound **2** (2 mg). Subfraction L3 (15 mg) was further purified by RP-HPLC, applying an isocratic system of H_2_O-MeOH (28:72), which led to the isolation of compound **3** (1.1 mg) and compound **5** (1.2 mg). Compound **6** (7.9 mg) was obtained by further purification of fraction K4 (8.8 mg) via preparative Prep TLC, using *n*-hexane–acetone (1:1) as developing system. Fraction G11 (250 mg) was first separated by HPLC (mobile phase: H_2_O-MeOH, 20:75) on a LiChrospher RP18 column, affording 3 subfractions (M1-3). RP-HPLC was applied for the final purification of M2 (10.3 mg) by using a H_2_O–MeOH (20:80) solvent system to yield compound **4** (1.2 mg). In our previous work [12] on *P. populnea*, fraction EA3 (370 mg) was obtained from the ethyl acetate phase using multiple steps of chromatographic methods. Fraction EA3 (370 mg) was further separated by NP-HPLC using cyclohexane–EtOAc (2:8) as mobile phase, and 3 subfractions (EA3a-c) were yielded. Compound **7** (1.2 mg) was isolated from fraction EA3a (90 mg) by RP-HPLC, using a linear gradient system of H_2_O–MeOH from 75% to 95% MeOH, and then an isocratic system of H_2_O–MeOH (10:90). 

### 3.4. Spectroscopic and Physical Characteristic of Compounds

Pholiol L (**1**) colorless amorphous solid; [*α*]^23^_D_ −11.6 (*c* 0.1, CHCl_3_); for ^1^H and ^13^C NMR spectroscopic data, see Table 1 and Table 2; HRESIMS *m*/*z* 647.4147 [M + H]^+^ (calcd for C_37_H_59_O_9_^+^ 647.4154).

Pholiol M (**2**) colorless amorphous solid; [*α*]^23^_D_ −10.9 (*c* 0.05, CHCl_3_); for ^1^H and ^13^C NMR spectroscopic data, see Table 1 and Table 2; HRESIMS *m*/*z* 703.4078 [M-H]^−^ (calcd for C_39_H_59_O_11_^−^ 703.4063).

Pholiol N (**3**) white amorphous powder; [*α*]^23^_D_ −8.9 (*c* 0.05, CHCl_3_); for ^1^H and ^13^C NMR spectroscopic data, see Table 1 and Table 2; HRESIMS *m*/*z* 727.4041 [M + Na]^+^ (calcd for C_39_H_60_O_11_Na^+^ 727.4028).

Pholiol O (**4**) colorless amorphous solid; [*α*]^27^_D_ +11.8 (*c* 0.05, CHCl_3_); for ^1^H and ^13^C NMR spectroscopic data, see Table 1 and Table 2; HRESIMS *m*/*z* 725.3877 (M + Na)^+^, (calcd for C_39_H_58_O_11_Na^+^ 725.3871) and 703.4063 [M + H]^+^ (calcd for C_39_H_59_O_11_^+^ 703.4052).

Pholiol *p* (**5**) white amorphous powder; [*α*]^23^ −1.6 (*c* 0.05, MeOH); for ^1^H and ^13^C NMR spectroscopic data, see Table 1 and Table 2; HRESIMS *m*/*z* 683.3767 [M + Na]^+^ (calcd for C_37_H_56_O_10_Na^+^ 683.3766).

Pholiol Q (**6**) colorless amorphous solid; [*α*]^27^_D_ +5.0 (*c* 0.1, MeOH); for ^1^H and ^13^C NMR spectroscopic data, see Table 1 and Table 2; HRESIMS *m*/*z* 657.4010 [M–H]^−^ (calcd for C_38_H_57_O_9_^−^, 657.4008).

Pholiol R (**7**) colorless white amorphous solid; [*α*]^22^_D_ +24.9 (*c* 0.05, MeOH); for ^1^H and ^13^C NMR spectroscopic data, see Table 1 and Table 2; HRESIMS *m*/*z* 613.3768 [M–H]^−^ (calcd for C_36_H_53_O_8_^−^ 613.3746).

Pholiol S (**8**) colorless amorphous solid; [*α*]^23^_D_ −14.2 (*c* 0.1, CHCl_3_); for ^1^H and ^13^C NMR spectroscopic data, see Table 1 and Table 2; HRESIMS *m*/*z* 669.3623 [M + Na]^+^ (calcd for C_36_H_54_O_10_Na^+^ 669.3609).

### 3.5. Cytotoxic and Antiproliferative Assays

*Cell Line Cultures*: The human colon adenocarcinoma cell lines, Colo 205 (ATCC-CCL-222) doxorubicin-sensitive parent and Colo320/MDR-LRP (ATCC-CCL-220.1) doxorubicin resistant expressing ABCB1, were purchased from LGC Promochem (Teddington, UK). The cells were cultured in RPMI-1640 medium supplemented with 10% heat-inactivated fetal bovine serum (FBS), 2 mM L-glutamine, 1 mM Na-pyruvate, 100 mM HEPES. The MRC-5 (ATCC CCL-171) human embryonic lung fibroblast cell line and the hormone-responsive breast cancer cell line MCF-7 (ATCC HTB-22) were purchased from Sigma-Aldrich (Merck KGaA, Darmstadt, Germany). The MCF-7 and MRC-5 cells were cultured in Eagle’s Minimal Essential Medium (EMEM) containing 4.5 g/L of glucose and supplemented with a non-essential amino acid mixture, a selection of vitamins, and 10% FBS. The cell lines were incubated at 37 °C, 5% CO_2_, and 95% air atmosphere. The cells were detached with Trypsin-Versene (EDTA) solution for 5 min at 37 °C. The human non-small cell lung cancer cell line A549 was kindly provided by Brigitte Marian (Institute of Cancer Research, Department of Medicine I, Medical University of Vienna, Austria). A549 cells were grown in Eagle’s Minimal Essential Medium (EMEM) supplemented with 10% heat-inactivated fetal bovine serum.

*Antiproliferative Assays*: The antiproliferative effect of the compounds was tested in decreasing serial dilutions of compounds (starting with 100 µM, then two-fold serial dilution) on human cell lines (Colo 205, Colo 320, MCF-7, A549, and MRC-5) in 96-well flat-bottomed microtiter plates. Firstly, the compounds were diluted in 100 µL of the medium, and then, 6 × 10^3^ cells (Colo 205, Colo 320) in 100 µL of RPMI medium were added to each well, excluding the medium control wells. The adherent MCF-7, A549, and MRC-5 cells (6 × 10^3^ cells/well) were seeded in EMEM medium for at least 4 h before the assay. The two-fold serial dilutions of the compounds were made in a separate plate (100–0.19 μM) and then transferred to the plates containing the adherent-corresponding cell line. Culture plates were incubated at 37 °C for 72 h, and at the end of the incubation period, 20 μL of MTT (thiazolyl blue tetrazolium bromide) solution (from a 5 mg/mL stock solution) was added to each well and incubated for an additional 4 h. Then, 100 μL of sodium dodecyl sulfate (SDS) solution (10% SDS in 0.01 M HCl) was added to each well, and the plates were further incubated at 37 °C overnight in a CO_2_ incubator. Cell growth was determined by measuring the optical density (OD) at 540/630 nm with a Multiscan EX ELISA reader (Thermo Labsystems, Cheshire, WA, USA). The percentage of inhibition of cell growth was determined according to Equation (1), and expressed as IC_50_ values, defined as the concentration that induces 50% growth inhibition. IC_50_ values and the standard deviation (SD) of triplicate experiments were calculated using GraphPad Prism 5 software for Windows. Doxorubicin (2 mg/mL, Teva Pharmaceuticals, Budapest, Hungary) was used as a positive control and the vehicle DMSO as the negative control (Table 3).

(1) $$IC_{50}=\left[\frac{OD_{sample}-OD_{medium\;control}}{OD_{cell\;control}-OD_{medium\;control}}\right]\times 100$$

Assay for Cytotoxic Effect: The effects of increasing concentrations of the compounds on cell growth were tested in 96-well flat-bottomed microtiter plates, as described for the antiproliferative assay, using 1 × 10^4^ cells/well. The culture plates were incubated at 37 °C for 24 h; at the end of the incubation period, 20 μL of MTT solution (from a 5 mg/mL stock solution) was added to each well. After incubation at 37 °C for 4 h, 100 μL of sodium dodecyl sulfate (SDS) solution (10% SDS in 0.01 M HCl) was added to each well, and the plates were further incubated at 37 °C overnight. Cell growth was determined by measuring the optical density (OD) at 540 nm (ref 630 nm) with a Multiscan EX ELISA reader (Thermo Labsystems, Cheshire, WA, USA). Inhibition of cell growth was expressed as IC_50_ (Table 4).

Sample preparation: The compounds were dissolved in DMSO to achieve the final concentration of 5 mM. The starting concentration of the compounds was 100 µM and 2-fold serial dilutions were prepared for the antiproliferative and cytotoxic assays. The following concentrations were used: 100-50-25-12.5-6.25-3.125-1.56-0.78-0.39-0.195 µM.

## Figures and Tables

**Table 1:** ^1^H NMR data of pholiols L–Q (**1**–**8**) [(500 MHz, *δ* ppm (*J* = Hz)].

No.	1 ^a^	2 ^a^	3 ^a,c^	4 ^b^	5 ^b,c^	6 ^b^	7 ^b^	8 ^a^
1 *α*	1.44 m	1.70 t (12.1)	2.18 t (11.6)	1.40 m	1.31 m	1.49 m	1.81 m	1.53 t (12.3)
1 *β*	2.21 dd (12.5, 3.8)	2.87 dd (12.1, 4.1)	2.26 dd (11.6, 4.3)	3.25 dd (13.0, 4.6)	3.23 dd (12.8, 4.6)	2.39 m	2.64 m	2.21 dd (12.5, 4.3)
2	3.92 dt (3.8, 10.5)	5.29 dt (4.1, 10.9)	5.17 dt (4.3, 10.9)	5.24 dt (4.6, 11.0)	5.14 dt (4.6, 11.0)	5.24 dt (4.3, 10.6)	5.74 dd (13.9, 5.5)	5.19 dt (4.3, 10.9)
3	4.60 d (10.5)	4.83 d (10.9)	4.87 d (10.9)	4.81 d (11.0)	3.28 d (11.0)	4.75 d (10.6)	–	4.83 d (10.9)
5	1.82 m	1.81 m	2.01 m	1.83 dd (14.9, 2.3)	1.75 dd (14.9, 2.2)	1.30 m	1.51 m	1.85 m
6 *α*	2.42 m (2H)	2.44 m	2.48 m (2H)	2.46 dd (15.7, 2.3)	2.50 dd (15.4, 2.2)	2.14 m (2H)	2.14 m	2.43 m (2H)
6 *β*		2.54 m		2.66 m	2.71 m		2.34 m	
7	–	–	–	–	–	5.53 t (3.9)	5.59 d (6.7)	–
11 *α*	2.32 m (2H)	4.57 br t (5.7)		–	–	5.39 d (6.1)	5.49 d (6.1)	2.29 m (2H)
11 *β*			4.49 m					
12 *α*	1.79 m (2H)	2.28 m	2.46 m	2.87 d (16.1)	2.90 d (16.2)	2.25 d (17.5)	2.30 d (17.9)	1.78 m (2H)
12 *β*		1.88 m	1.84 m	2.58 d (16.1)	2.62 d (16.2)	2.14 dd (17.5, 6.1)	2.18 dd (17.9, 6.1)	
15	2.07 m, 1.74 m	2.08 m, 1.83 m	2.08 m, 1,65 m	2.13 m, 1.82 m	2.17 m, 1.82 m	1.66 m, 1.43 m	1.70 m, 1.47 m	2.07 m, 1.73 m
16	2.00 m, 1.37 m	2.04 m, 1.45 m	2.00 m, 1.33 m	2.04 m, 1.46 m	2.08 m, 1.49 m	2.03 m, 1.42 m	2.06 m, 1.45 m	2.00 m, 1.38 m
17	1.42 m	1.43 m	1.55 m	1.76 m	1.80 m	1.63 m	1.66 m	1.43 m
18	0.66 s	0.89 s	0.65 s	0.84 s	0.88 s	0.61 s	0.67 s	0.65 s
19	1.24 s	1.50 s	1.34 s	1.44 s	1.47 s	1.14 s	1.40 s	1.30 s
20	1.40 m	1.43 m	1.36 m	1.44 m	1.49 m	1.44 m	1.45 m	1.43 m
21	0.91 d (6.2)	0.94 d (6.2)	0.91 d (6.3)	0.92 d (7.1)	0.97 d (6.6)	0.93 d (6.6)	0.95 d (6.7)	0.92 d (6.7)
22	1.83 m, 1.31 m	1.83 m, 1.31 m	1,82 m, 1.30 m	1.80 m, 1.25 m	1.82 m, 1.33 m	1.77 m, 1.23 m	1.79 m, 1.26 m	1.85 m, 1.35 m
23	2.52 m (2H)	2.53 m (2H)	2.55 m, 2.49 m	2.68 m (2H)	2.72 m (2H)	2.67 m (2H)	2.67 m (2H)	2.40 m, 2.27 m
26	1.38 s	1.38 s	1.38 s	1.29 s	1.34 s	1.29 s	1.31 s	
27	1.38 s	1.38 s	1.39 s	1.29 s	1.34 s	1.29 s	1.31 s	
28	0.91 s	0.90 s	0.92 s	0.93 s	1.11 s	0.91 s	1.11 s	0.90 s
29	0.96 s	1.03 s	1.01 s	1.04 s	1.00 s	1.03 s	1.26 s	1.00 s
30	0.92 s	0.86 s	1.12 s	0.84 s	1.25 s	0.93 s	0.94 s	0.91 s
2′	2.75 m, 2.57 m	2.68 m, 2.54 m	2.63 m, 2.53 m	2.63 m (2H)	2.78 m, 2.73 m	2.66 m, 2.54 m	2.81 m, 2.74 m	2.67 m, 2.52 m
4′	1.42 s	1.35 s	1.35 s	1.35 s	1.44 s	1.33 s	1.44 s	1.33 s
5′	2.92 m, 2.27 m	2.70 m, 2.62 m	2.68 m, 2.62 m	2.68 m (2H)	2.76 m (2H)	2.54 m, 2.39 m	2.81 m, 2.74 m	2.70 m, 2.61 m
7′	3.72 s	3.71 s	3.71 s	3.67 s	3.73 s	–	–	–
OAc	–	2.07 s		2.06 s		2.06 s	–	2.06 s

^a^ measured in CDCl_3_; ^b^ in CD_3_OD; Signal of H-12 in the ^1^H NMR spectrum measured in CD_3_OD was 4.48 dd (*J* = 9.1, 5.5 Hz); ^c^ measured at 600 MHz.

**Table 2:** ^13^C NMR data of pholiols L–Q (**1**–**8**) (125 MHz, *δ* ppm).

Position	1 ^a^	2 ^a^	3 ^a,c^	4 ^b^	5 ^b,c^	6 ^b^	7 ^b^	8 ^a^
1	42.78	39.49	39.90	40.51	40.38	42.53	43.71	40.36
2	66.89	69.89	70.36	70.63	73.22	71.13	73.41	70.10
3	84.53	79.44	79.06	80.56	79.83	81.69	210.63	79.18
4	38.98	39.59	39.47	40.23	40.62	40.31	49.01	39.45
5	49.77	50.29	49.75	50.93	51.36	50.41	53.05	49.50
6	36.56	36.57	36.80	37.14	37.39	23.91	24.59	36.44
7	198.29	199.71	199.18	202.76	203.47	121.19	123.19	198.02
8	139.13	139.69	141.71	152.20	152.11	143.87	144.02	139.33
9	164.11	160.48	159.15	151.87	152.11	146.28	145.50	163.27
10	40.68	40.85	40.62	41.54	41.68	39.75	39.50	40.67
11	23.99	67.31	65.55	203.60	203.95	118.31	118.71	23.98
12	30.28	42.90	44.60	52.54	52.52	38.96	38.94	30.10
13	45.13	43.57	44.60	48.58	48.88	44.96	44.82	45.10
14	47.98	49.14	48.26	50.38	50.35	51.54	51.60	47.98
15	32.06	32.15	32.78	33.32	33.30	32.56	32.57	32.02
16	28.82	28.88	27.75	28.13	28.15	28.74	28.75	28.73
17	49.11	48.43	49.93	50.32	50.29	52.29	52.31	48.94
18	15.95	17.45	16.98	17.32	17.22	16.31	16.34	15.95
19	19.89	21.18	21.18	19.07	18.93	23.89	23.20	19.56
20	36.10	36.14	35.96	37.01	37.01	37.13	37.13	36.01
21	18.70	18.76	18.52	18.81	18.80	18.82	18.81	18.51
22	30.19	30.19	30.08	30.84	30.77	31.05	31.04	31.11
23	32.73	32.75	32.69	33.84	33.79	33.97	33.98	31.05
24	215.01	214.92	214.82	217.59	217.92	217.78	217.74	178.71
25	76.34	76.38	76.16	77.89	77.97	77.90	77.90	–
26	26.73	26.75	26.73	26.75	26.73	26.78	26.78	–
27	26.71	26.72	26.73	26.78	26.76	26.75	26.75	–
28	27.99	28.11	27.86	28.43	28.60	28.97	25.32	27.83
29	17.50	17.52	17.36	17.71	17.09	18.25	22.22	17.42
30	25.19	25.28	25.44	26.02	25.98	26.08	26.10	25.16
1′	171.41	171.44	171.66	171.94	172.54	172.17	171.66	171.41
2′	46.33	44.99	45.06	46.32	46.59	47.22	46.76	45.10
3′	70.37	69.72	69.81	70.60	70.94	70.76	71.15	69.70
4′	28.29	27.70	27.75	27.97	27.89	27.80	27.61	27.65
5′	43.68	45.04	45.15	45.96	45.97	47.70	46.79	44.88
6′	173.26	172.28	172.12	173.13	173.44	171.74	171.66	172.24
7′	52.05	51.94	52.00	51.93	51.99			
Ac*C*O		170.84	170.68	172.52	–	172.64		170.75
Ac*C*H_3_		21.07	21.08	20.96.	–	21.05		21.04

^a^ measured in CDCl_3_; ^b^ in CD_3_OD; ^c^ measured at 150 MHz.

**Table 3:** Antiproliferative activity (IC_50_ µM) of pholiols L, M, O, Q, and S (**1**, **2**, **4**, **6**, and **8**) in human sensitive (Colo 205) and resistant (Colo 320) colon adenocarcinoma cells. and normal embryonal fibroblast (MRC-5) cell line.

Compd.	Colo 205		Colo 320		MCF-7		A549		MRC-5	
	Mean	SD	Mean	SD	Mean	SD	Mean	SD	Mean	SD
**1**	32.41	0.89	28.07	4.62	**21.74**	0.88	53.73	1.27	70.86	1.22
**2**	**23.91**	0.026	32.51	2.97	**2.48**	0.16	>100	–	>100	–
**4**	**23.39**	0.060	42.14	0.15	**9.95**	0.37	51.53	1.61	42.89	1.34
**6**	49.97	0.52	69.19	2.67	46.28	1.86	51.21	1.04	>100	–
**8**	59.87	0.55	68.54	4.40	35.33	3.03	89.84	0.75	>100	–
Dox	0.0617	0.0128	0.25	0.06	0.155	0.0027	1.04	0.097	0.52	0.018
DMSO	>2%	–	>2%	–	>2%	–	>2%	–	>2%	–

Dox = doxorubicin.

**Table 4:** Cytotoxic activity in human sensitive (Colo 205) and resistant (Colo 320) colon adenocarcinoma cells and relative resistance ratio of pholiols L, M, O, Q, and S (**1**, **2**, **4**, **6**, and **8**).

Compd.	IC_50_ (µM)				RR ^a^	IC_50_ (µM)					
	Colo 205 (A)		Colo 320 (B)		(B/A)	MCF-7		A549		MRC-5	
	Mean	SD	Mean	SD		Mean	SD	Mean	SD	Mean	SD
**1**	40.33	1.64	33.92	1.84	**0.84**	43.69	0.03	93.61	1.94	66.08	1.36
**2**	35.93	0.44	67.22	3.86	1.87	>100	–	58.12	0.70	>100	–
**4**	31.52	0.91	91.52	4.96	2.90	>100	–	56.86	1.53	57.99	0.82
**6**	56.12	0.84	34.73	1.24	**0.62**	43.78	0.18	85.88	2.41	>100	–
**8**	57.50	0.96	57.52	2.36	1.0	42.99	0.61	83.65	6.06	55.27	0.41
Dox	3.32	0.083	11.96	0.88	3.60	5.73	1.02	10.22	0.07	3.60	0.35
DMSO	>2%	–	>2%	–		>2%	–	>2%	–	>2%	–

^a^ RR (relative resistance ratio) = IC_50_ resistant/IC_50_ sensitive; Dox = doxorubicin

## Data Availability

Data are contained within the article and Supplementary Materials.

## Supplementary Materials

The following supporting information can be downloaded at: https://www.mdpi.com/article/10.3390/ph16010104/s1, Figures S1–S56: NMR and MS spectra.
