# Supplementary material for: Isolation of the Lanostane Triterpenes Pholiols L–S from Pholiota populnea and Evaluation of Their Antiproliferative and Cytotoxic Activities
Source: Pharmaceuticals (Basel). 2023 Jan 10;16(1):104. doi: 10.3390/ph16010104 (PMC9862111; doi:10.3390/ph16010104)
Supplement: Supplementary file 1 [file pharmaceuticals-16-00104-s001.zip › pharmaceuticals-2147556-supplementary.pdf]

SUPPORTING INFORMATION

Isolation of the Lanostane Triterpenes Pholiols L–S from *Pholiota populnea* and evaluation of their Antiproliferative and Cytotoxic Activities

Morteza Yazdani [1], Anita Barta [1], Anasztázia Hetényi [2], Róbert Berkecz [3], Gabriella Spengler [4], Attila Ványolós [5], Judit Hohmann [1,6,7,*]

[1] *Institute of Pharmacognosy, University of Szeged, H-6720 Szeged, Hungary*

[2] *Department of Medical Chemistry, University of Szeged, 6720 Szeged, Szeged, Hungary*

[3] *Institute of Pharmaceutical Analysis, University of Szeged, 6720 Szeged, Hungary*

[4] *Department of Medical Microbiology, Albert Szent-Györgyi Health Center and Albert Szent-Györgyi Medical School, University of Szeged, H-6725 Szeged, Hungary*

[5] *Department of Pharmacognosy, Semmelweis University, H-1085 Budapest, Hungary*

[6] *Interdisciplinary Centre for Natural Products, University of Szeged, H-6720 Szeged, Hungary*

[7] *ELKH-USZ Biologically Active Natural Products Research Group, University of Szeged, H-6720 Szeged, Hungary*

* Correspondence: hohmann.judit@szte.hu; Tel.: +36-62-546453

CONTENT

Fig. S1. ^{1}H NMR spectrum of compound **1** (500 MHz, $CDCl_3$)....4
Fig. S2. ^{13}C JMOD spectrum of compound **1** (125 MHz, $CDCl_3$) 4
Fig. S3. ^{1}H,^{1}H-COSY spectrum of compound **1** (500 MHz, $CDCl_3$) 5
Fig. S4. HSQC spectrum of compound **1** (500/125 MHz, $CDCl_3$)....5
Fig. S5. HMBC spectrum of compound **1** (500/125 MHz, $CDCl_3$)....6
Fig. S6. NOESY spectrum of compound **1** (500 MHz, $CDCl_3$)....6
Fig. S7. HRESIMS spectrum of compound **1** 7
Fig. S8. ^{1}H NMR spectrum of compound **2** (500 MHz, $CDCl_3$)....7
Fig. S9. ^{13}C JMOD spectrum of compound **2** (125 MHz, $CDCl_3$) 8
Fig. S10. ^{1}H-^{1}H COSY spectrum of compound **2** (500 MHz, $CDCl_3$) 8
Fig. S11. HSQC spectrum of compound **2** (500/125 MHz, $CDCl_3$) 9
Fig. S12. HMBC spectrum of compound **2** (500/125 MHz, $CDCl_3$) 9
Fig. S13. NOESY spectrum of compound **2** (500 MHz, $CDCl_3$) 10
Fig. S14. HRESIMS spectrum of compound **2** 10
Fig. S15. ^{1}H NMR spectrum of compound **3** (600 MHz, $CDCl_3$) 11
Fig. S16. ^{13}C-JMOD spectrum of compound **3** (150 MHz, $CDCl_3$) 11
Fig. S17. ^{1}H-^{1}H COSY spectrum of compound **3** (600 MHz, $CDCl_3$) 12
Fig. S18. HSQC spectrum of compound **2** (600/150 MHz, $CDCl_3$) 12
Fig. S19. HMBC spectrum of compound **2** (600/150 MHz, $CDCl_3$) 13
Fig. S20. NOESY spectrum of compound **3** (600 MHz, $CDCl_3$) 13
Fig. S21. HRESIMS spectrum of compound **3** 14
Fig. S22. ^{1}H NMR spectrum of compound **4** (500 MHz, CD_3OD).... 14
Fig. S23. ^{13}C-JMOD spectrum of compound **4** (125 MHz, CD_3OD) 15
Fig. S24. ^{1}H-^{1}H COSY spectrum of compound **4** (500 MHz, CD_3OD) 15
Fig. S25. HSQC spectrum of compound **4** (500/125 MHz, $CDCl_3$) 16
Fig. S26. HMBC spectrum of compound **4** (500/125 MHz, $CDCl_3$) 16
Fig. S27. NOESY spectrum of compound **4** (500 MHz, CD_3OD) 17
Fig. S28. HRESIMS spectrum of compound **4** 17
Fig. S29. ^{1}H NMR spectrum of compound **5** (500 MHz, CD_3OD).... 18
Fig. S30. ^{13}C-JMOD spectrum of compound **5** (150 MHz, CD_3OD) 18
Fig. S31. ^{1}H-^{1}H COSY spectrum of compound **5** (600 MHz, CD_3OD) 19
Fig. S32. HSQC spectrum of compound **5** (600/150 MHz, CD_3OD) 19
Fig. S33. HMBC spectrum of compound **5** (600/150 MHz, CD_3OD) 20
Fig. S34. NOESY spectrum of compound **5** (600 MHz, CD_3OD) 20
Fig. S35. HRESIMS spectrum of compound **5** 21
Fig. S36. ^{1}H NMR spectrum of compound **6** (500 MHz, CD_3OD).... 21
Fig. S37. ^{13}C-JMOD spectrum of compound **6** (125 MHz, CD_3OD) 22
Fig. S38. ^{1}H-^{1}H COSY spectrum of compound **6** (500 MHz, CD_3OD) 22
Fig. S39. HSQC spectrum of compound **6** (500/125 MHz, CD_3OD) 23
Fig. S40. HMBC spectrum of compound **6** (500/125 MHz, CD_3OD) 23

Fig. S41. NOESY spectrum of compound **6** (500 MHz, CD_3OD) 24
Fig. S42. HRESIMS spectrum of compound **6** 24
Fig. S43. ^{1}H NMR spectrum of compound **7** (500 MHz, CD_3OD).......... 25
Fig. S44. ^{13}C-JMOD spectrum of compound **7** (125 MHz, CD_3OD) 25
Fig. S45. ^{1}H-^{1}H COSY spectrum of compound **7** (500 MHz, CD_3OD) 26
Fig. S46. HSQC spectrum of compound **7** (500/125 MHz, CD_3OD) 26
Fig. S47. HMBC spectrum of compound **7** (500/125 MHz, CD_3OD) 27
Fig. S48. NOESY spectrum of compound **7** (500 MHz, CD_3OD) 27
Fig. S49. HRESIMS spectrum of compound **7** 28
Fig. S50. ^{1}H NMR spectrum of compound **8** (500 MHz, $CDCl_3$).......... 28
Fig. S51. ^{13}C-JMOD spectrum of compound **8** (125 MHz, $CDCl_3$) 29
Fig. S52. ^{1}H-^{1}H COSY spectrum of compound **8** (500 MHz, $CDCl_3$) 29
Fig. S53. HSQC spectrum of compound **8** (500/125 MHz, $CDCl_3$) 30
Fig. S54. HMBC spectrum of compound **8** (500/125 MHz, $CDCl_3$) 30
Fig. S55. NOESY spectrum of compound **8** (500 MHz, $CDCl_3$) 31
Fig. S56. HRESIMS spectrum of compound **8** 31
Fig. S57. Flow chart of the isolation of compounds **1**–**8** 32

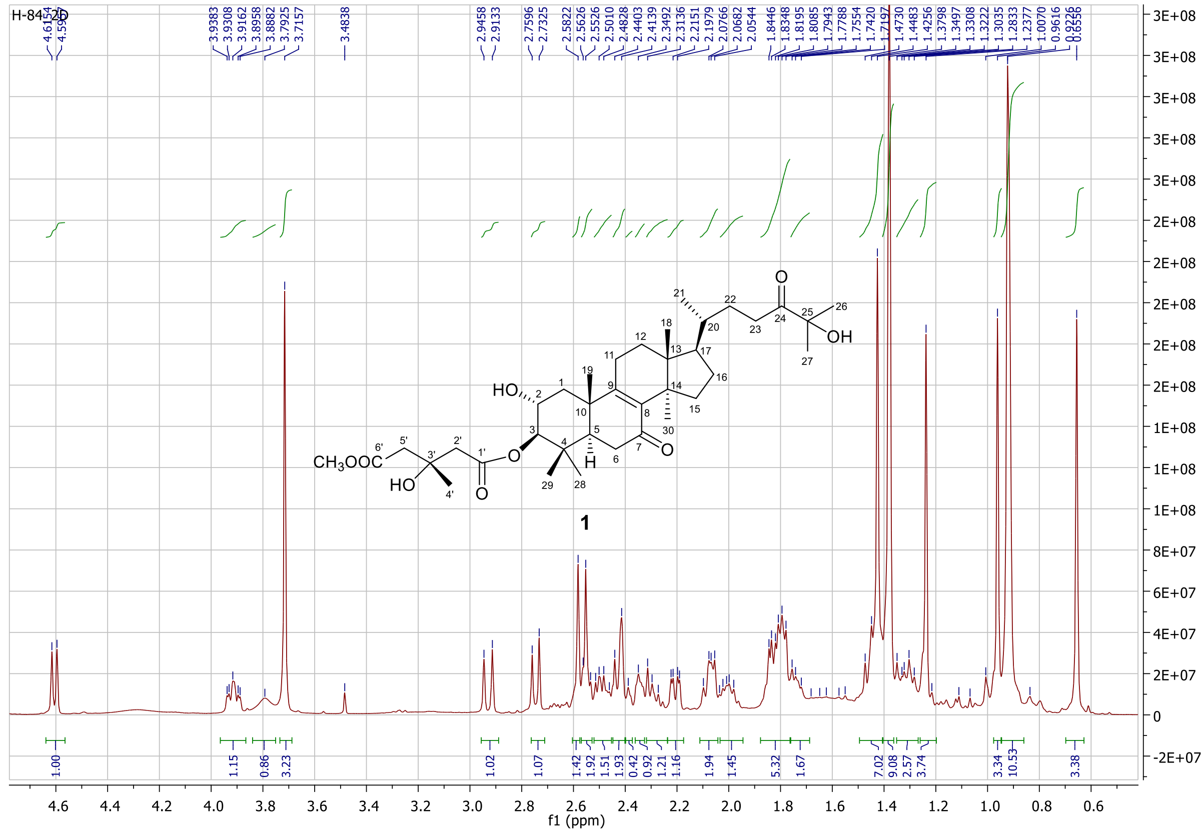

Fig. S1. ^{1}H NMR spectrum of compound **1** (500 MHz, $CDCl_3$)

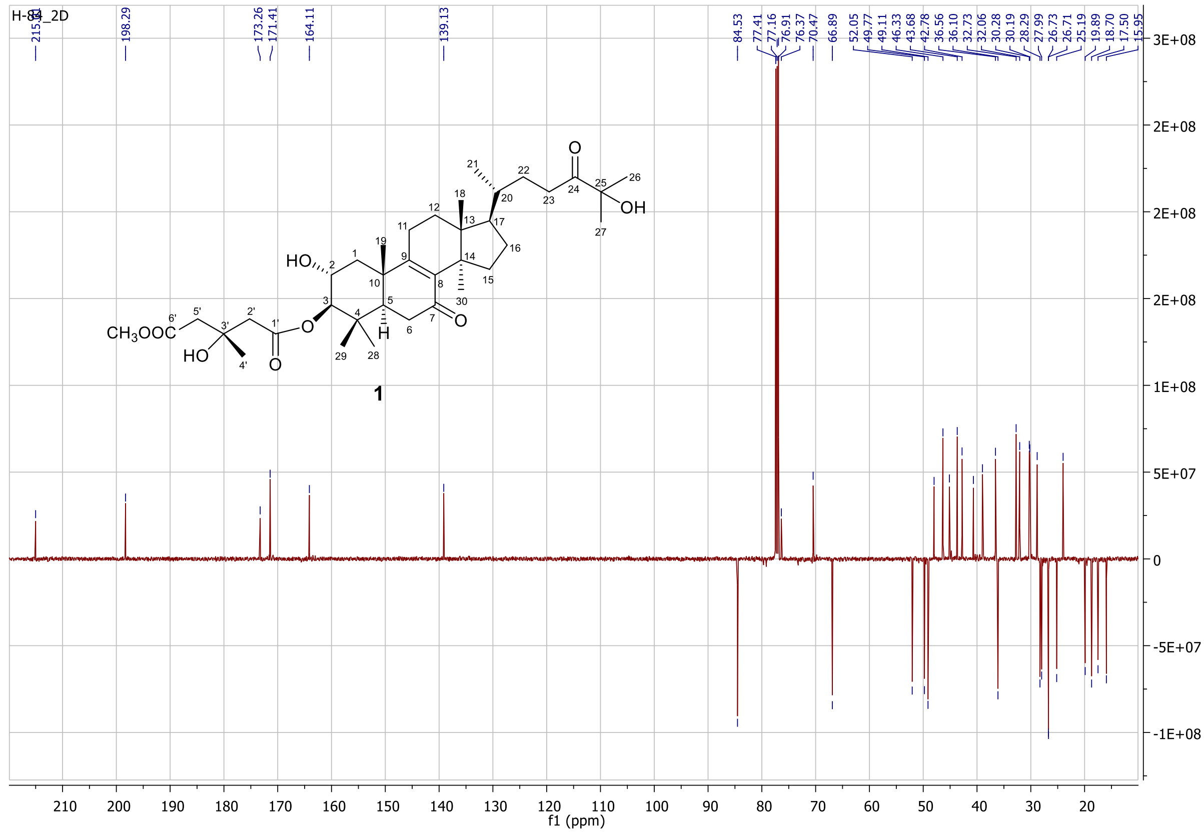

Fig. S2. ^{13}C JMOD spectrum of compound **1** (125 MHz, $CDCl_3$)

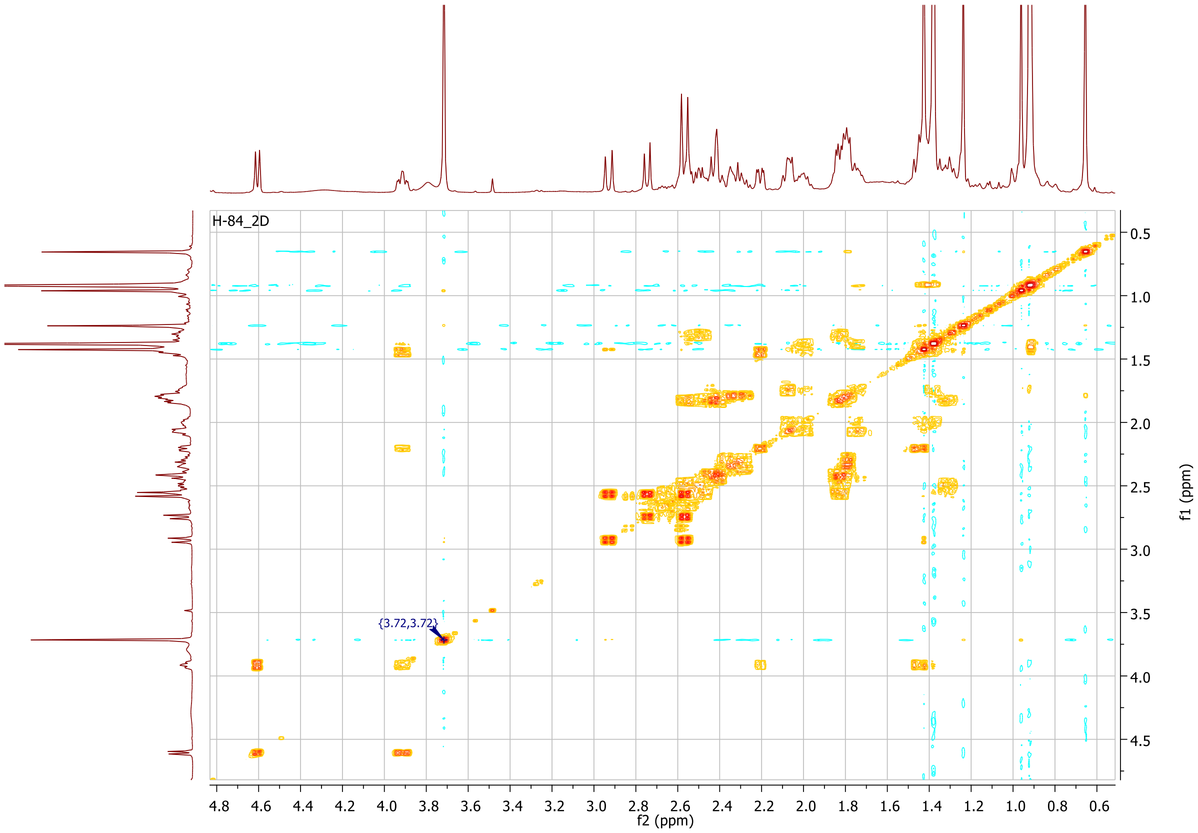

Fig. S3. $^{1}H,^{1}H$-COSY spectrum of compound **1** (500 MHz, $CDCl_3$)

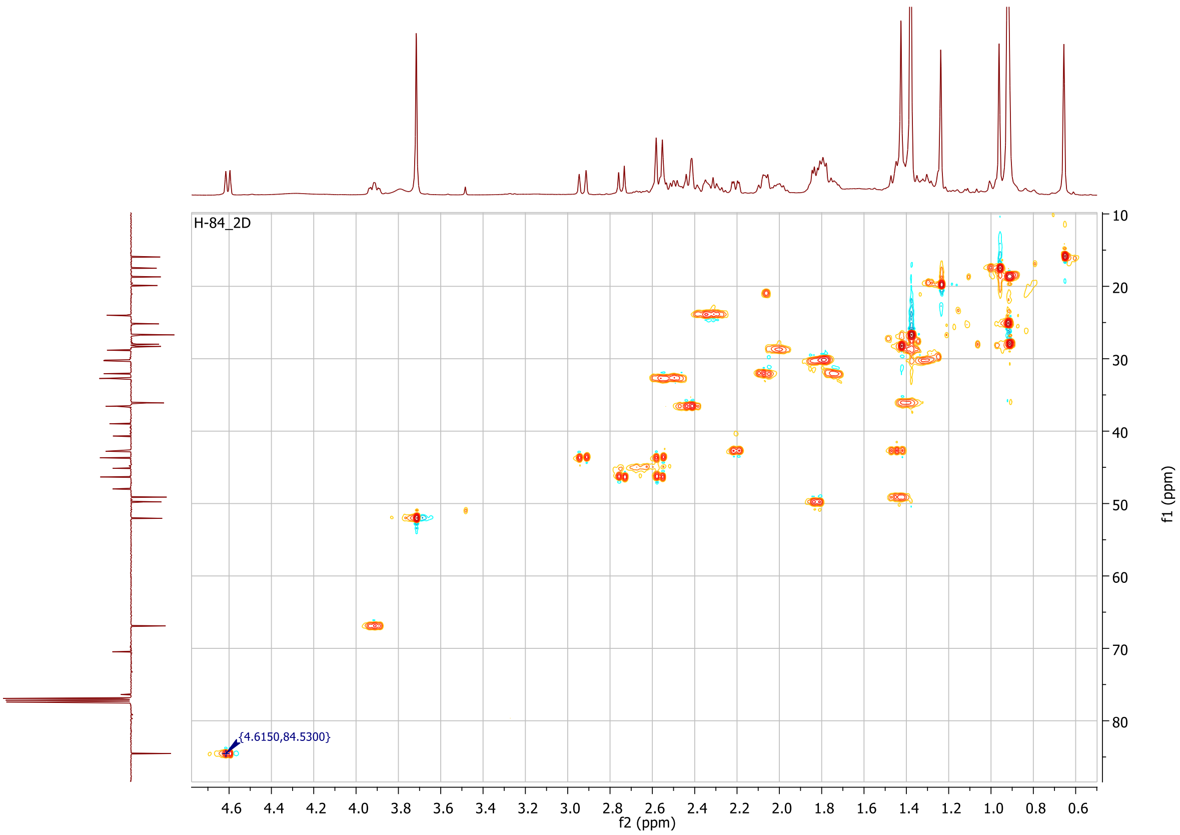

Fig. S4. HSQC spectrum of compound **1** (500/125 MHz, $CDCl_3$)

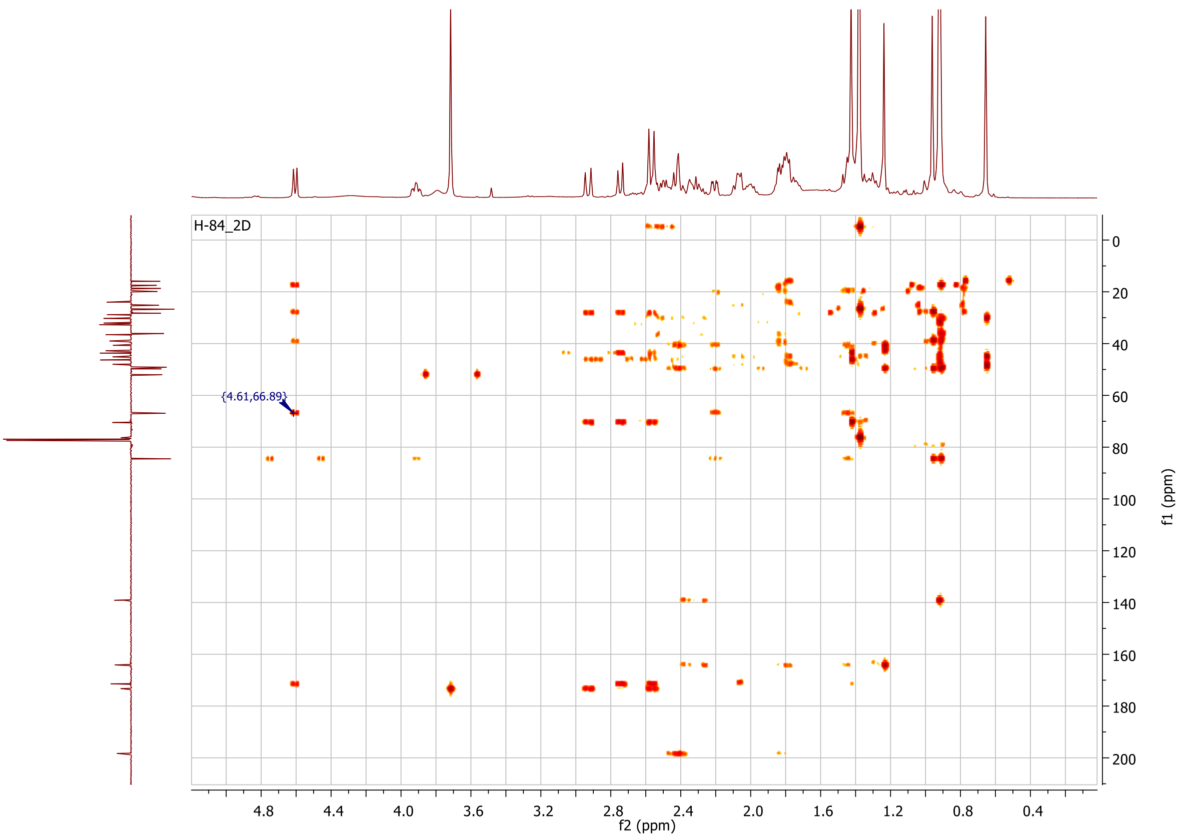

Fig. S5. HMBC spectrum of compound **1** (500/125 MHz, $CDCl_3$)

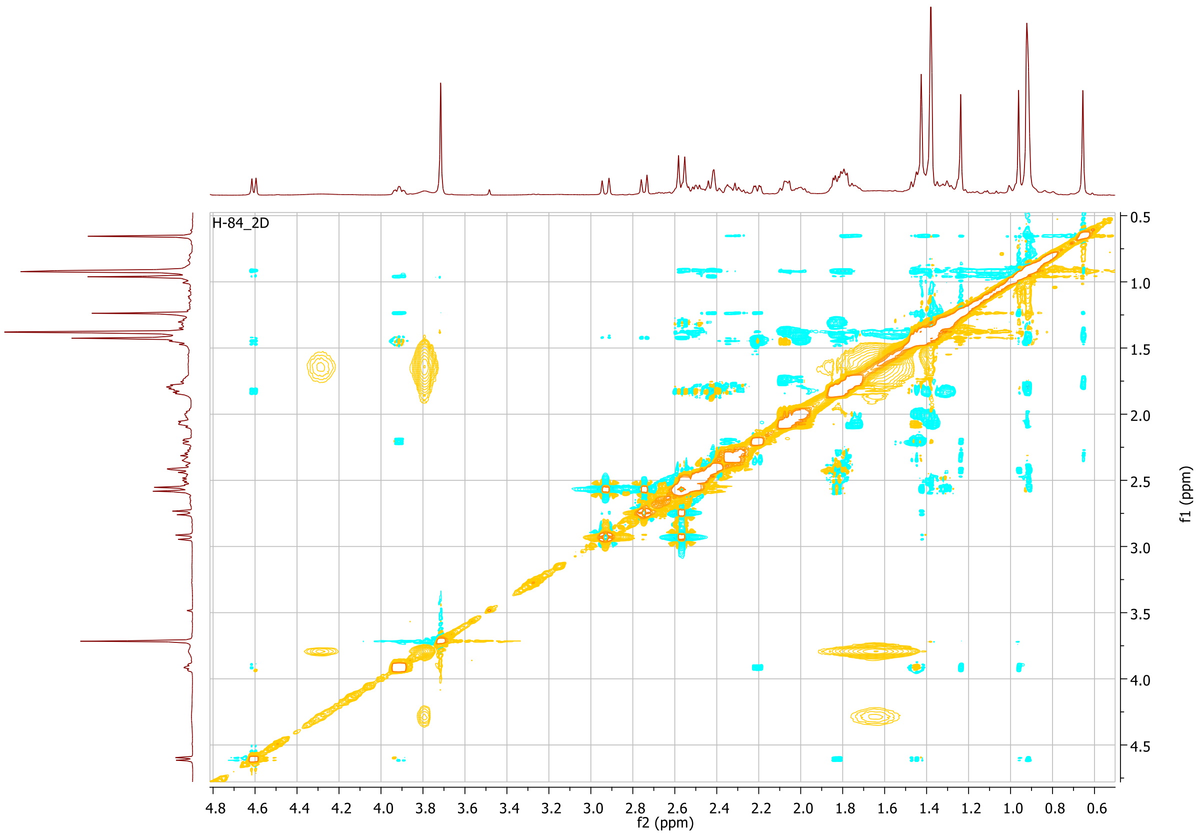

Fig. S6. NOESY spectrum of compound **1** (500 MHz, $CDCl_3$)

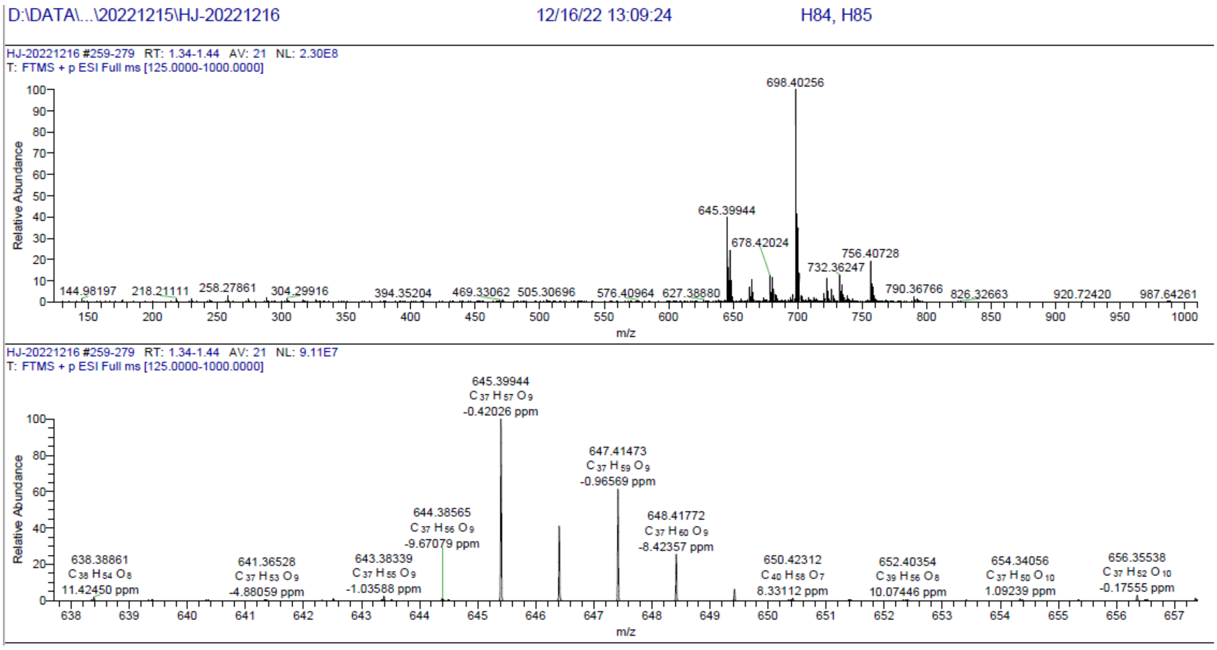

Fig. S7. HRESIMS spectrum of compound **1**

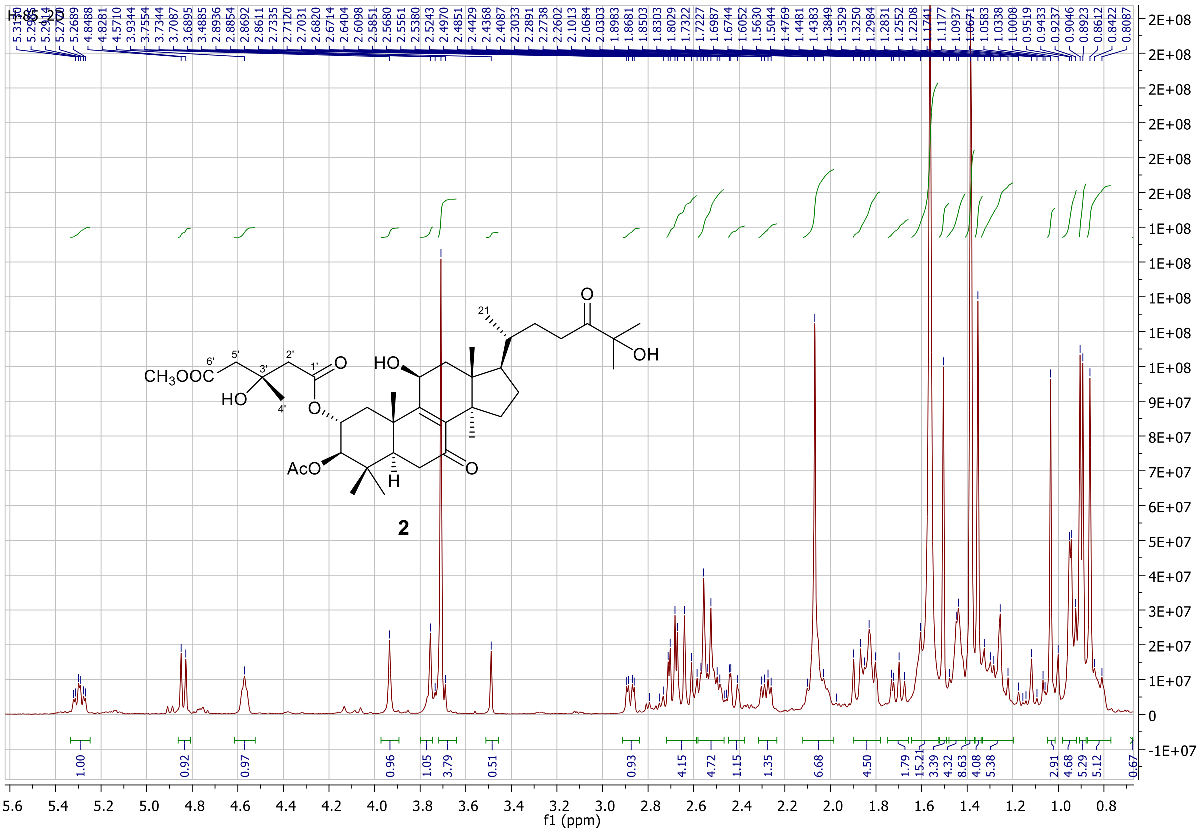

Fig. S8. ^{1}H NMR spectrum of compound **2** (500 MHz, $CDCl_3$)

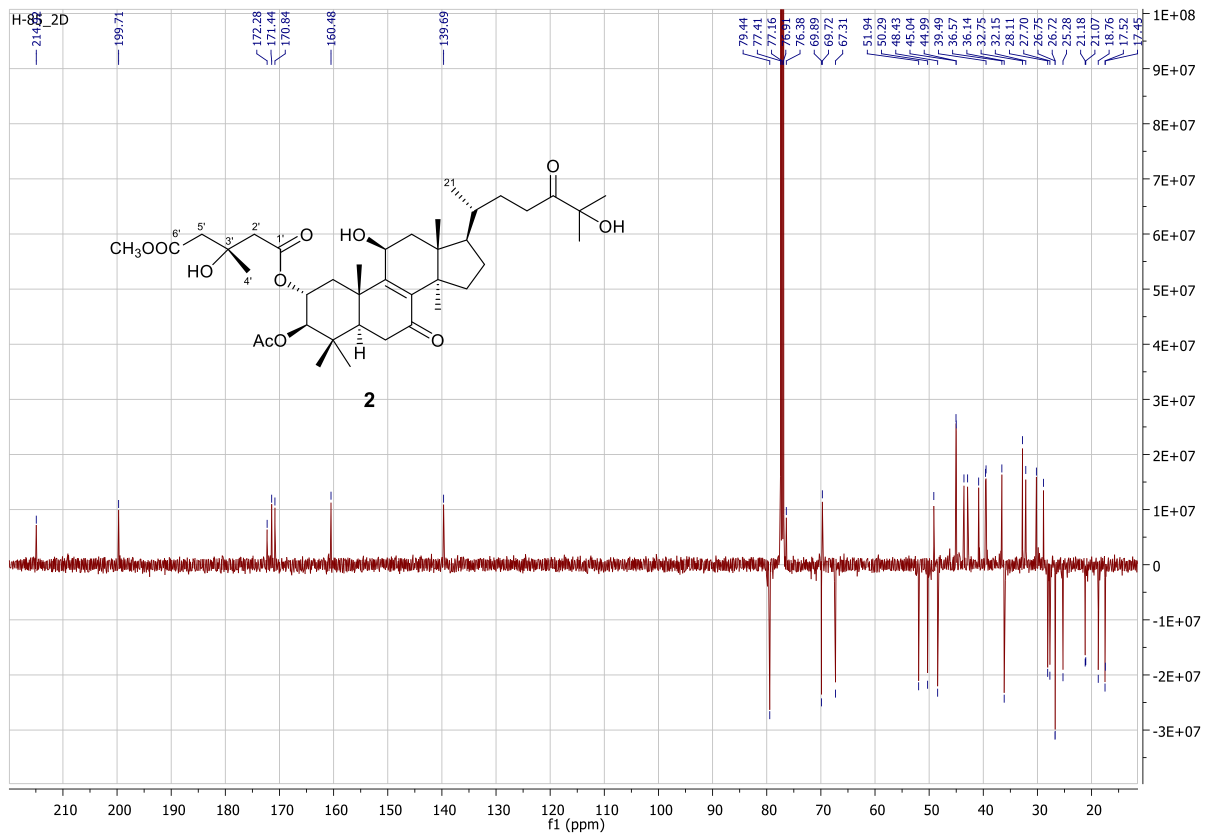

Fig. S9. ^{13}C JMOD spectrum of compound **2** (125 MHz, $CDCl_3$)

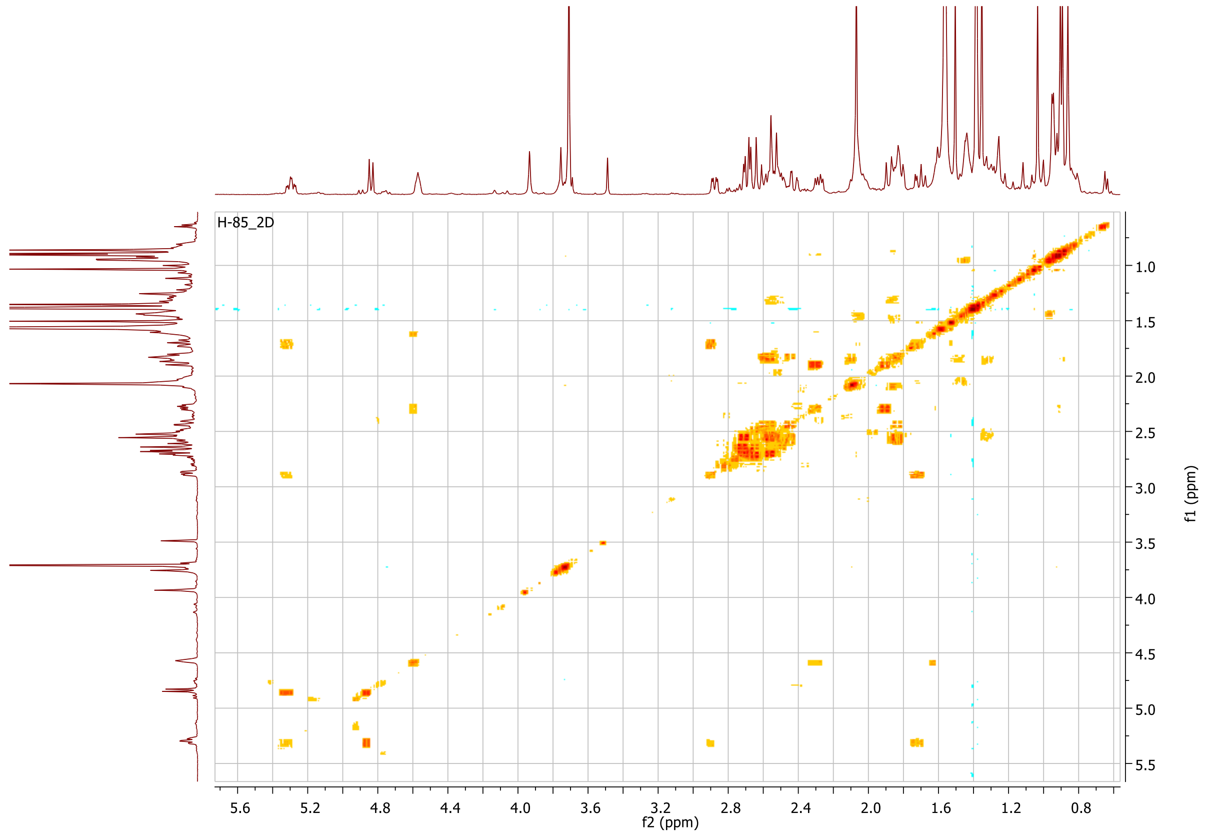

Fig. S10. ^{1}H-^{1}H COSY spectrum of compound **2** (500 MHz, $CDCl_3$)

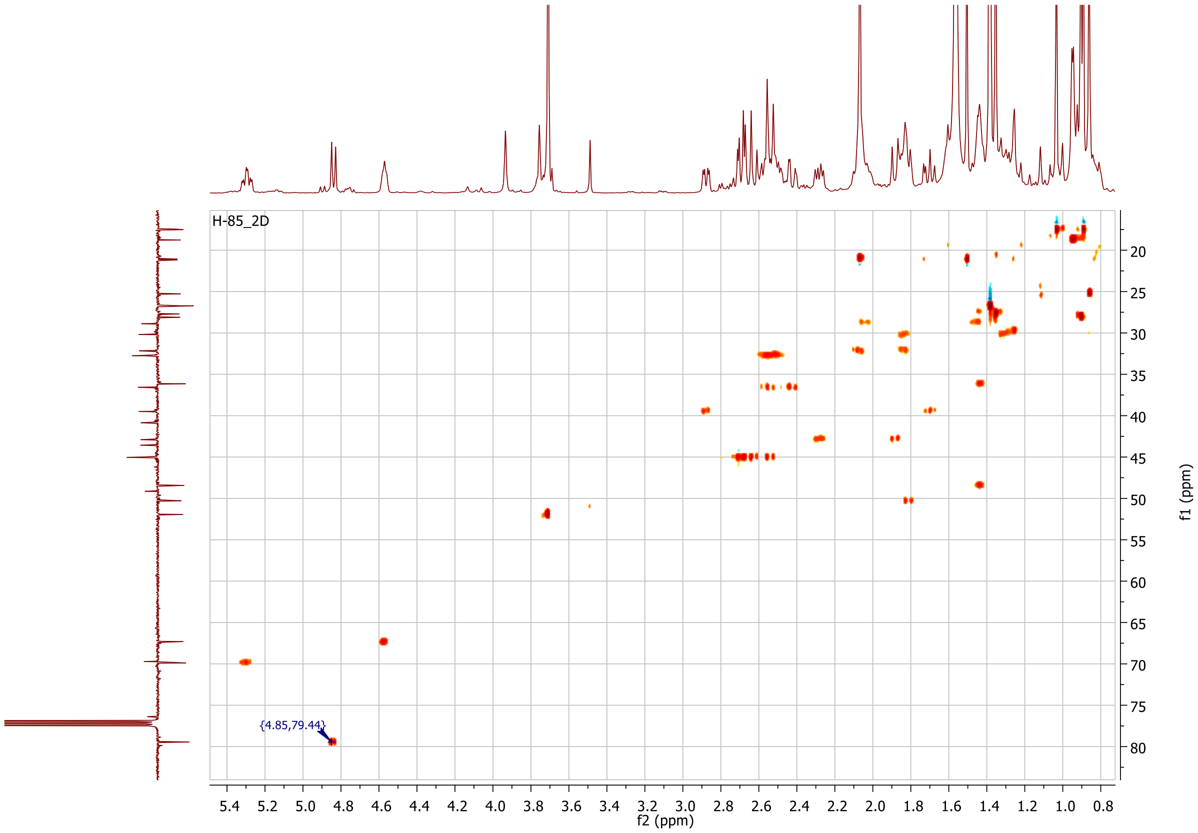

Fig. S11. HSQC spectrum of compound **2** (500/125 MHz, $CDCl_3$)

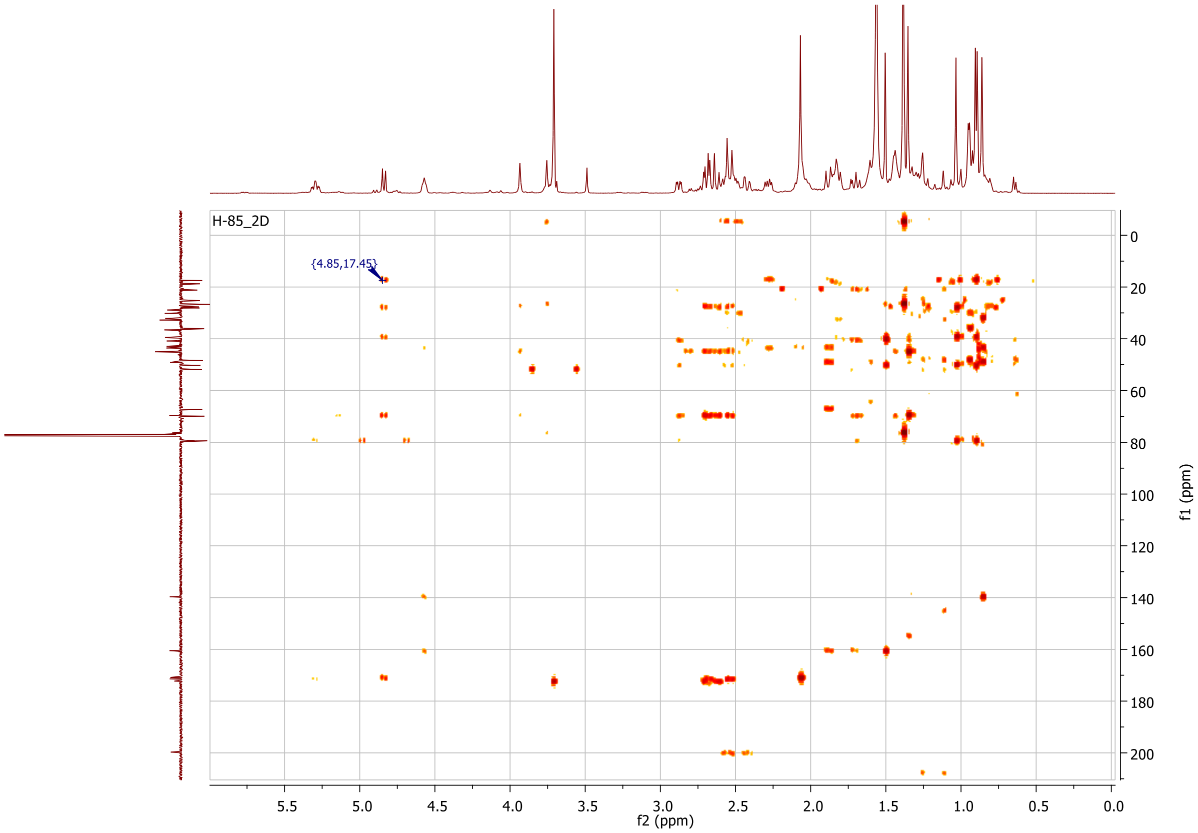

Fig. S12. HMBC spectrum of compound **2** (500/125 MHz, $CDCl_3$)

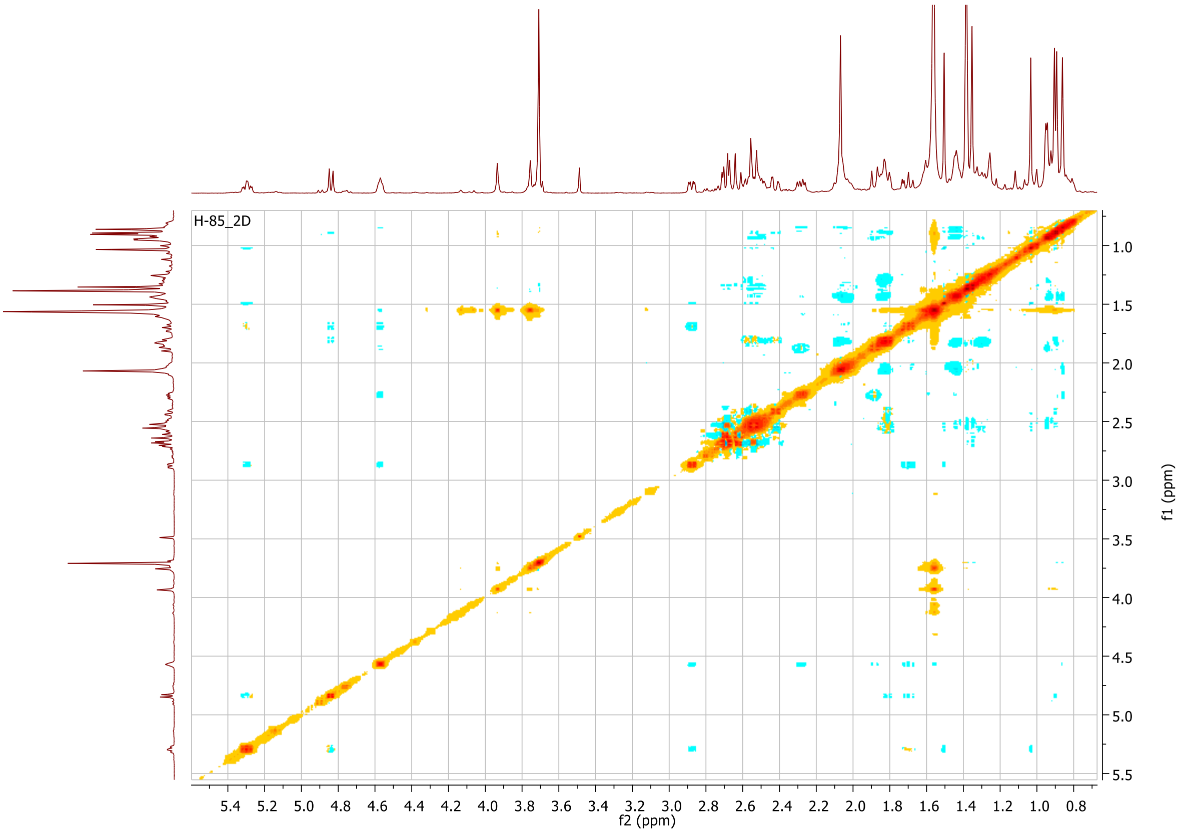

Fig. S13. NOESY spectrum of compound **2** (500 MHz, $CDCl_3$)

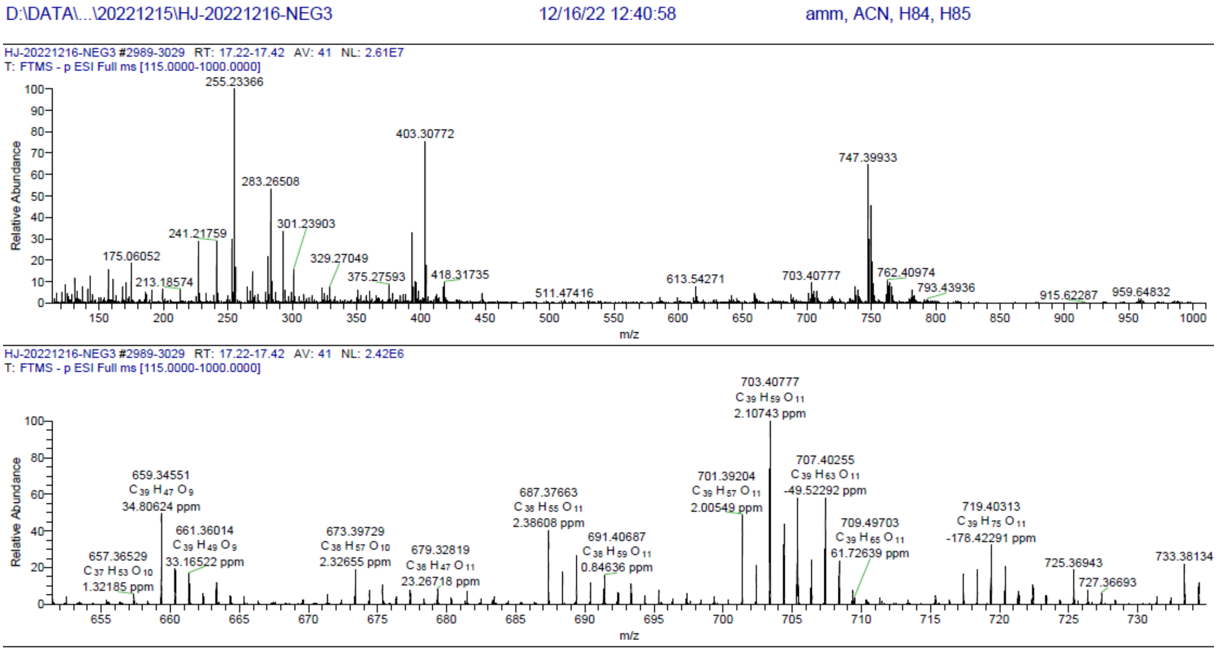

Fig. S14. HRESIMS spectrum of compound **2**

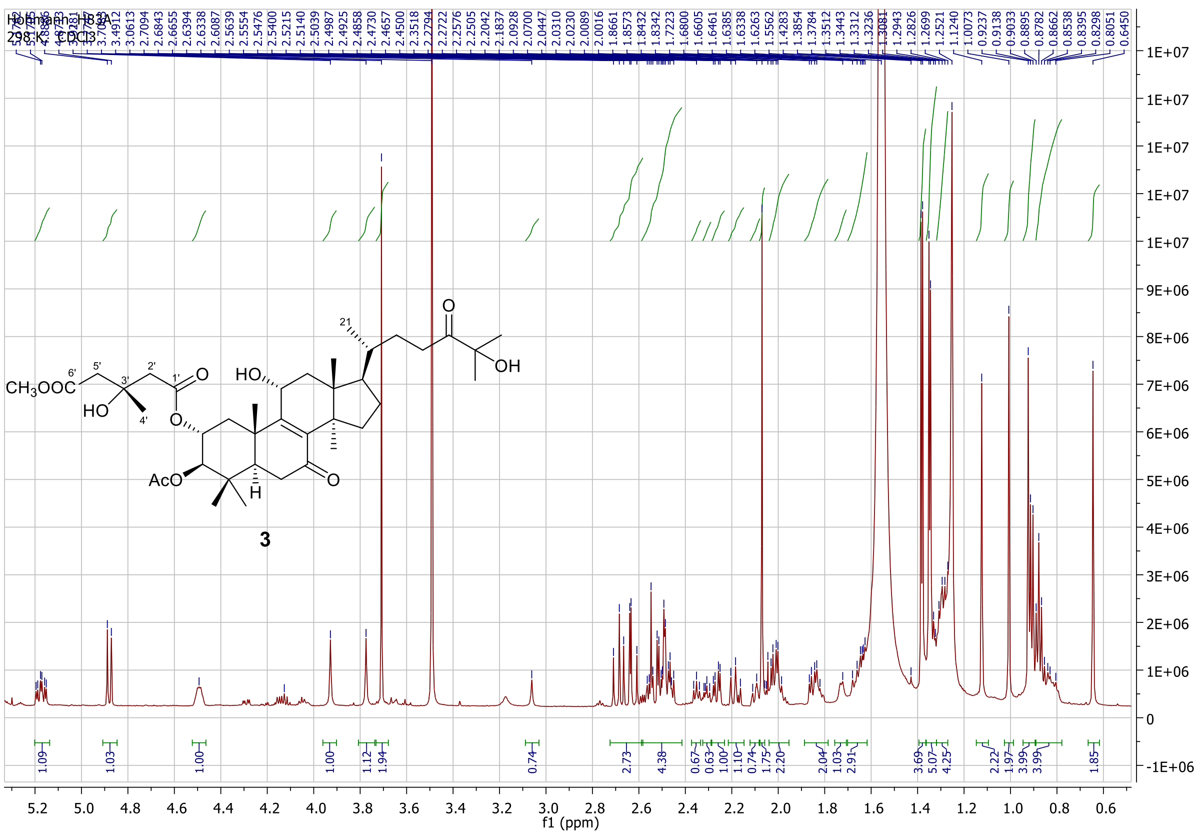

Fig. S15. ^{1}H NMR spectrum of compound **3** (600 MHz, $CDCl_3$)

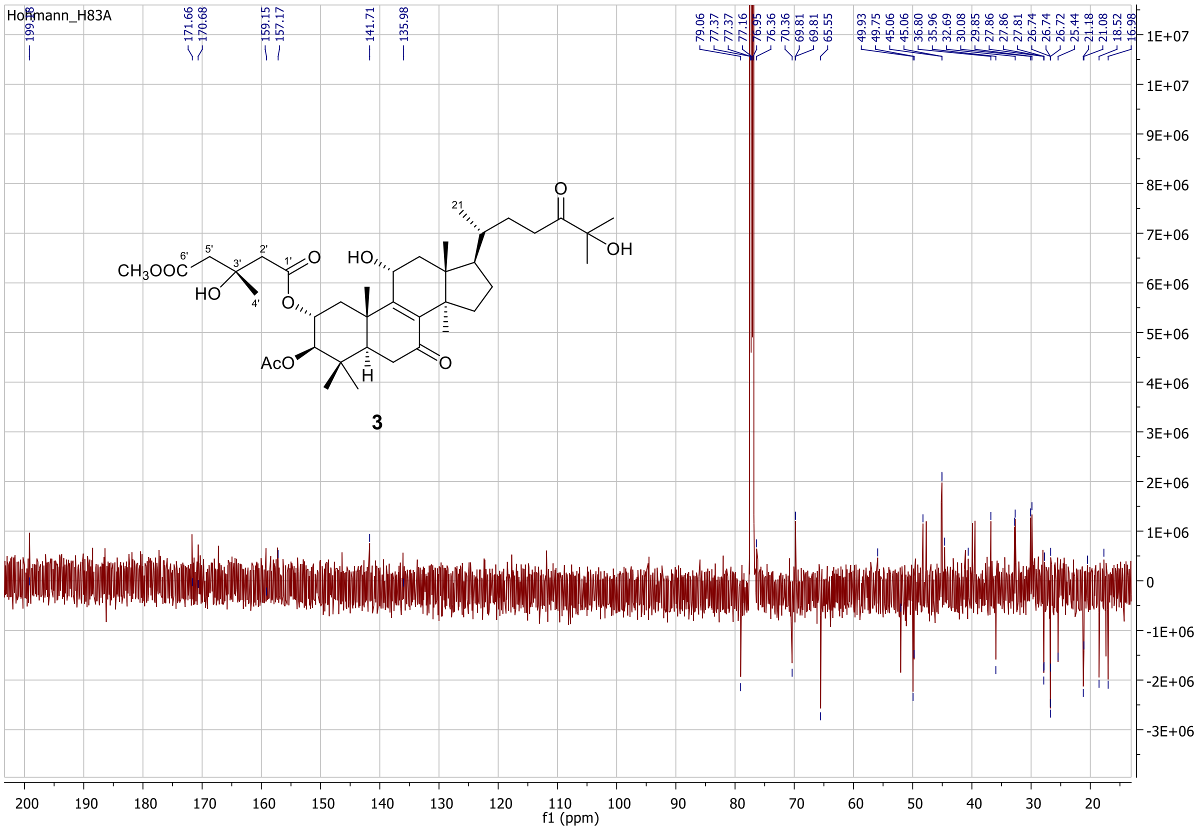

Fig. S16. ^{13}C-JMOD spectrum of compound **3** (150 MHz, $CDCl_3$)

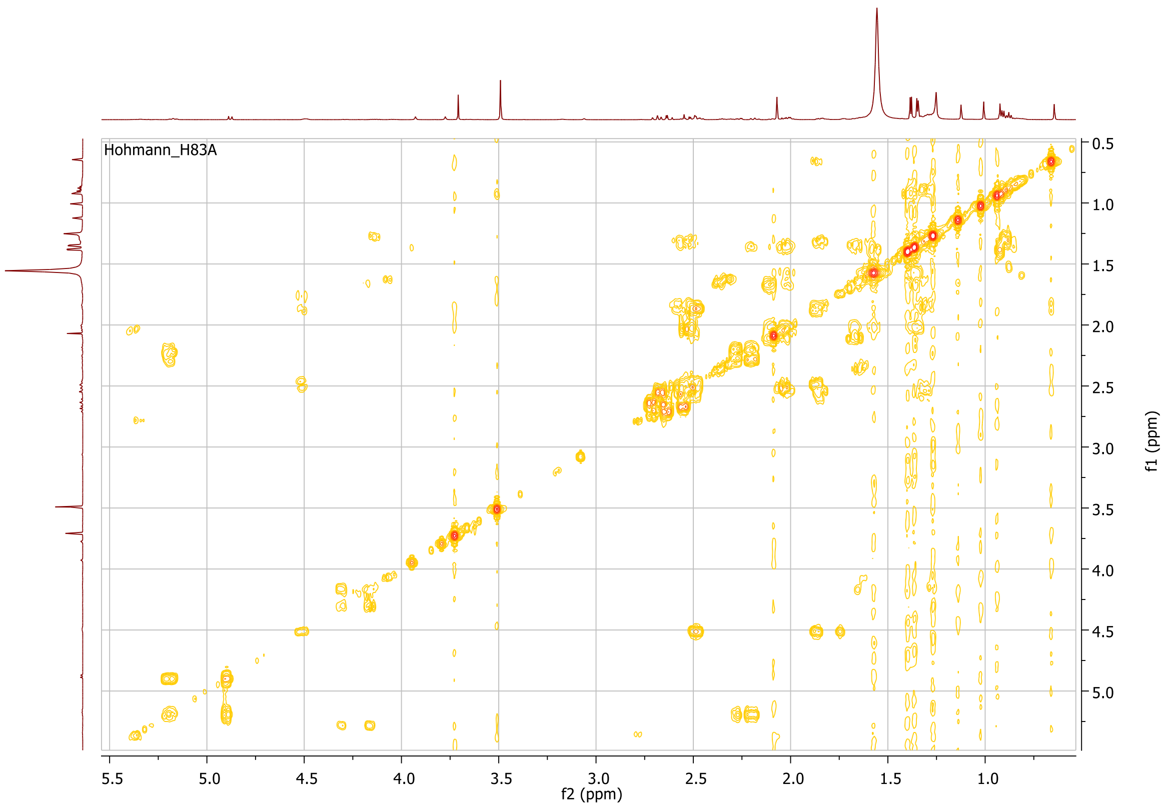

Fig. S17. ¹H-¹H COSY spectrum of compound **3** (600 MHz, $CDCl_3$)

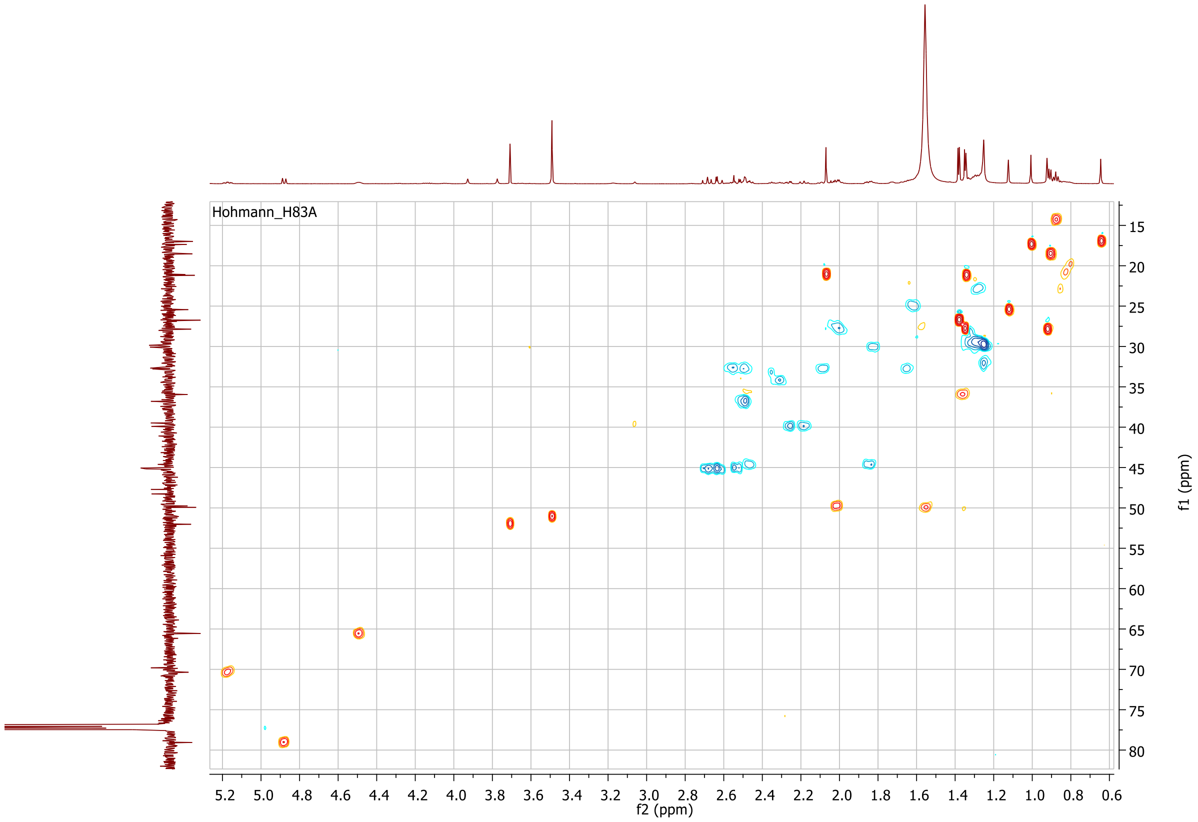

Fig. S18. HSQC spectrum of compound **2** (600/150 MHz, $CDCl_3$)

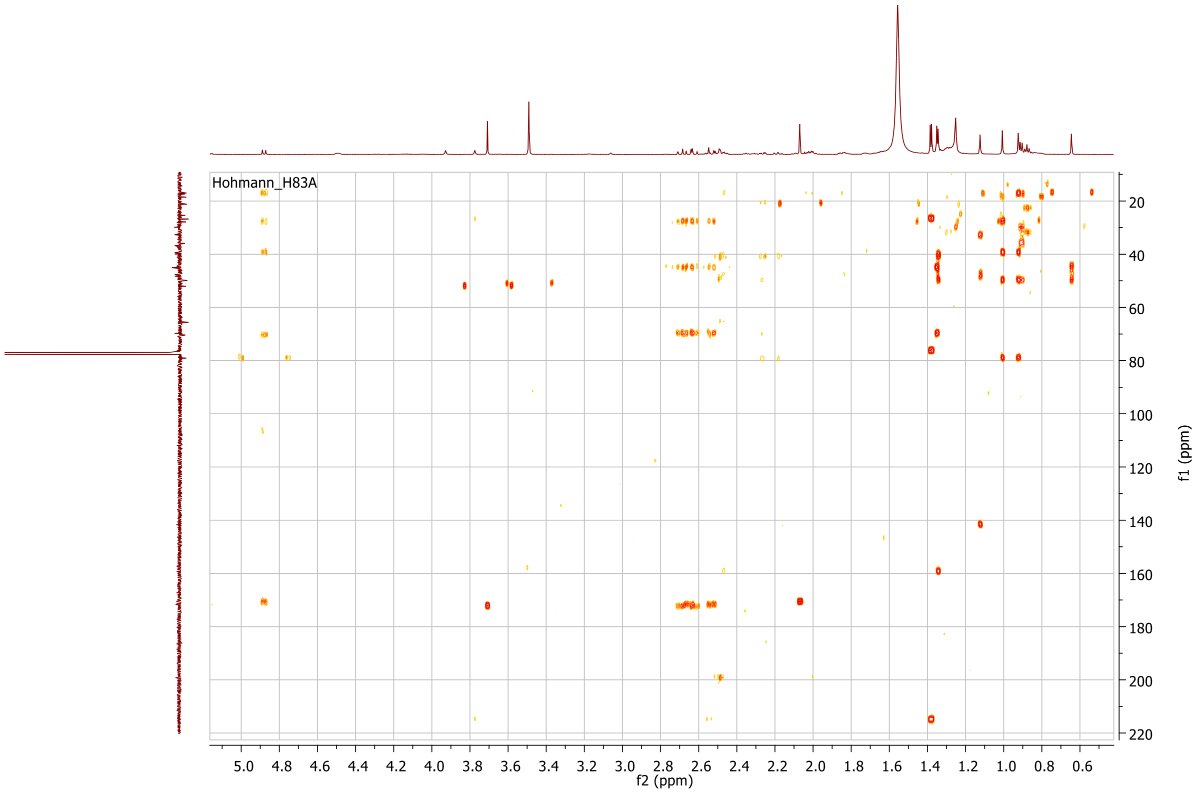

Fig. S19. HMBC spectrum of compound **2** (600/150 MHz, $CDCl_3$)

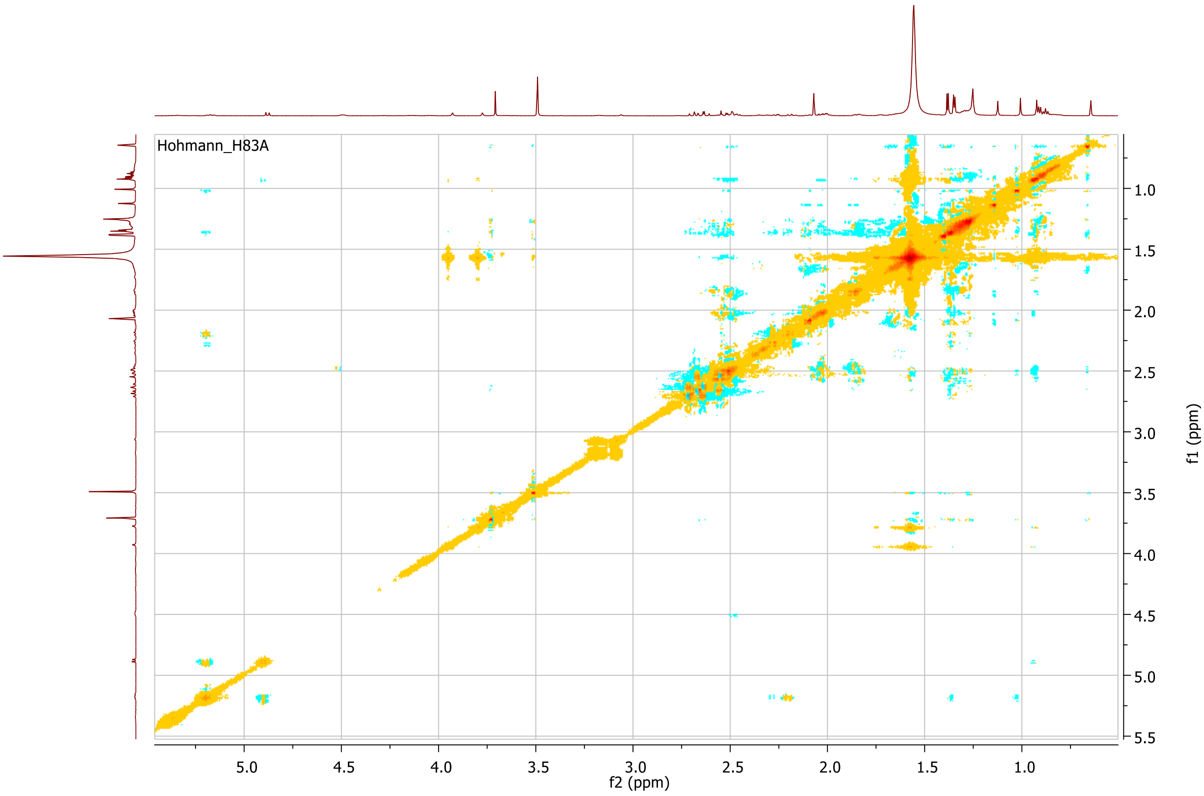

Fig. S20. NOESY spectrum of compound **3** (600 MHz, $CDCl_3$)

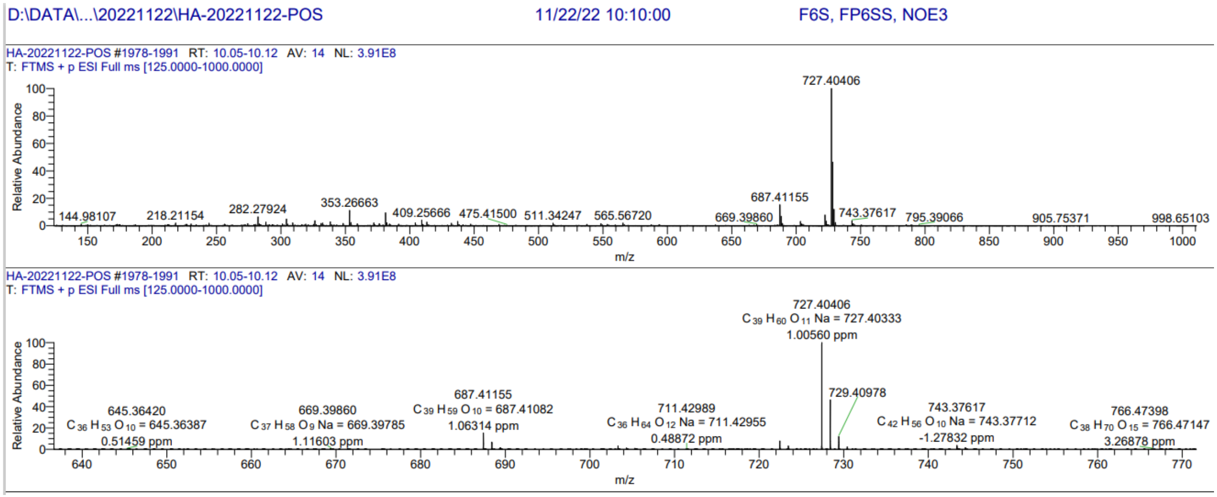

Fig. S21. HRESIMS spectrum of compound **3**

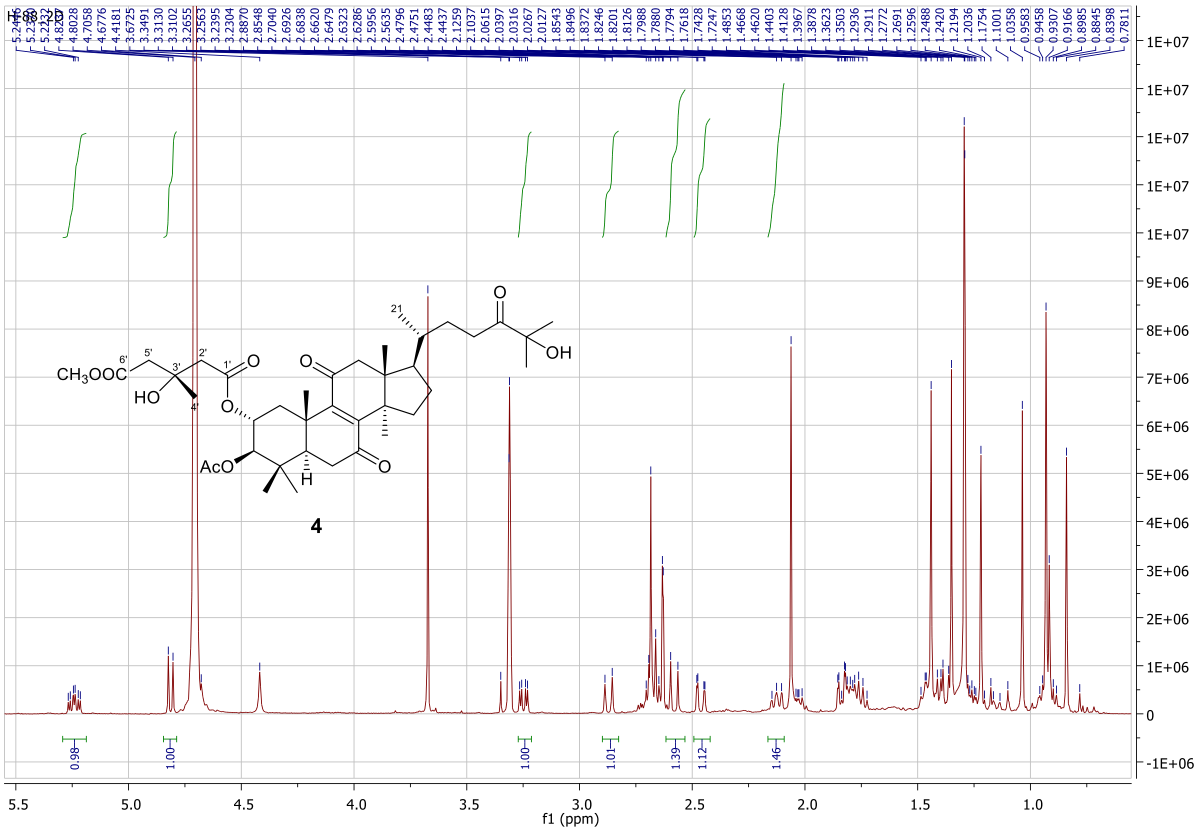

Fig. S22. ^{1}H NMR spectrum of compound **4** (500 MHz, CD_3OD)

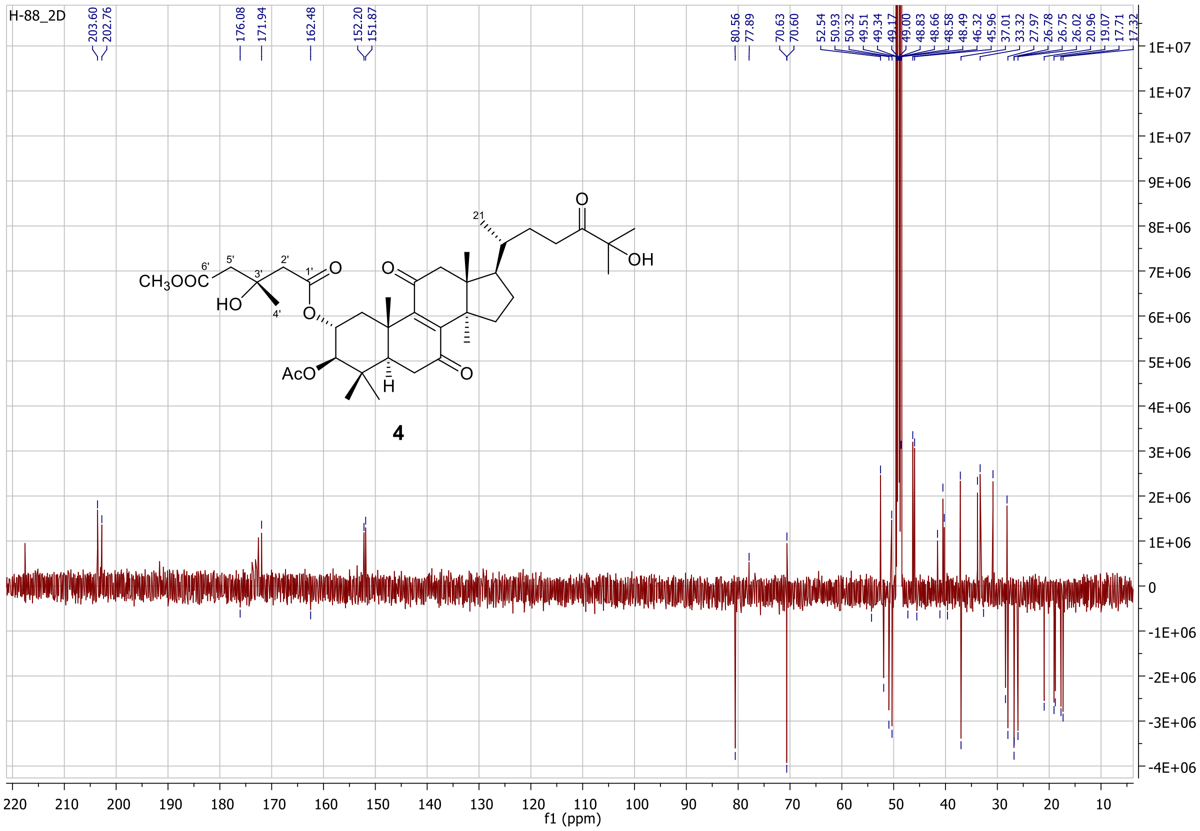

Fig. S23. ^{13}C-JMOD spectrum of compound **4** (125 MHz, CD_3OD)

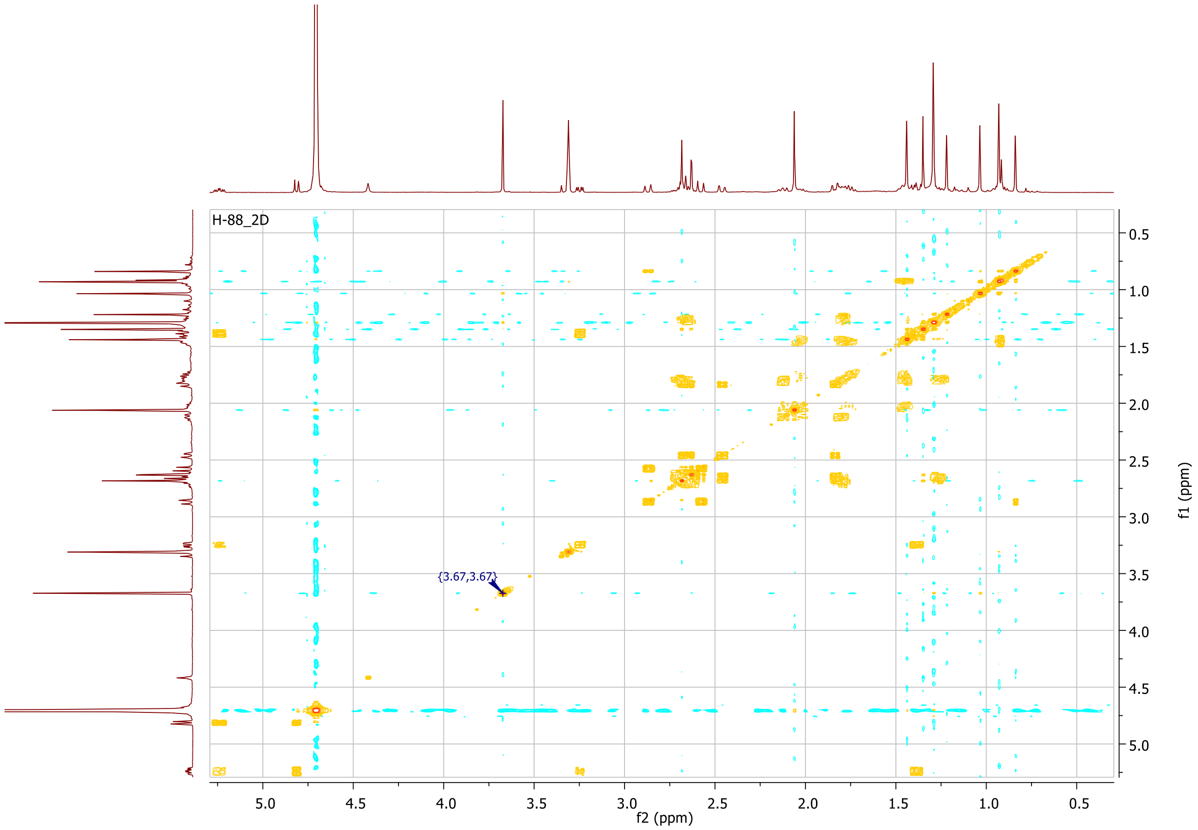

Fig. S24. ^{1}H-^{1}H COSY spectrum of compound **4** (500 MHz, CD_3OD)

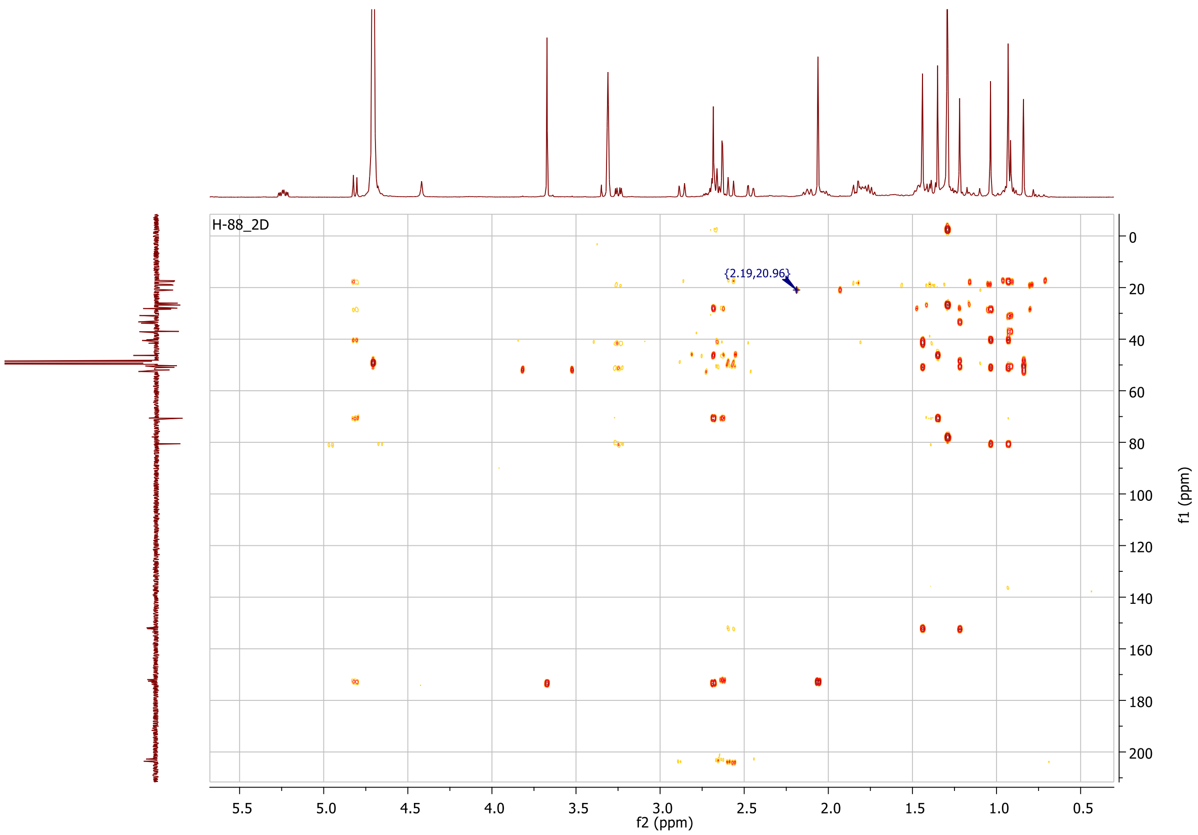

Fig. S25. HSQC spectrum of compound **4** (500/125 MHz, $CDCl_3$)

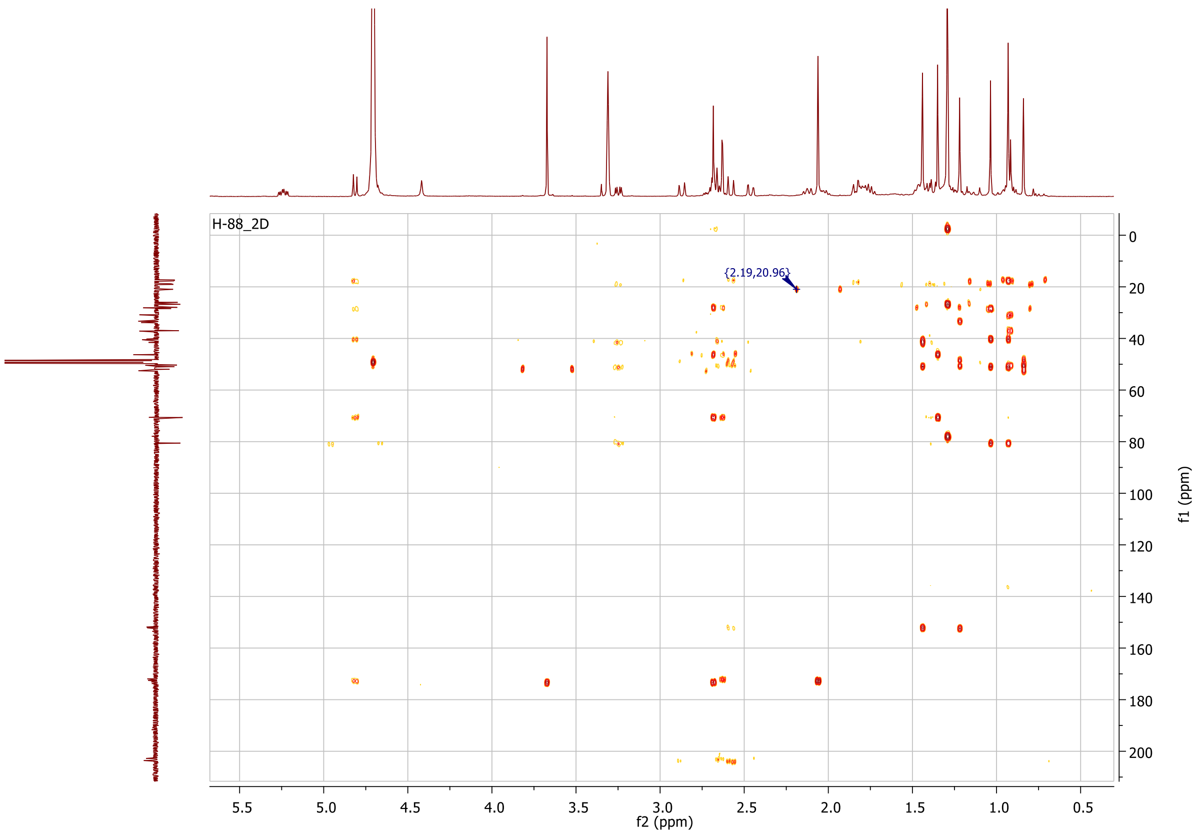

Fig. S26. HMBC spectrum of compound **4** (500/125 MHz, $CDCl_3$)

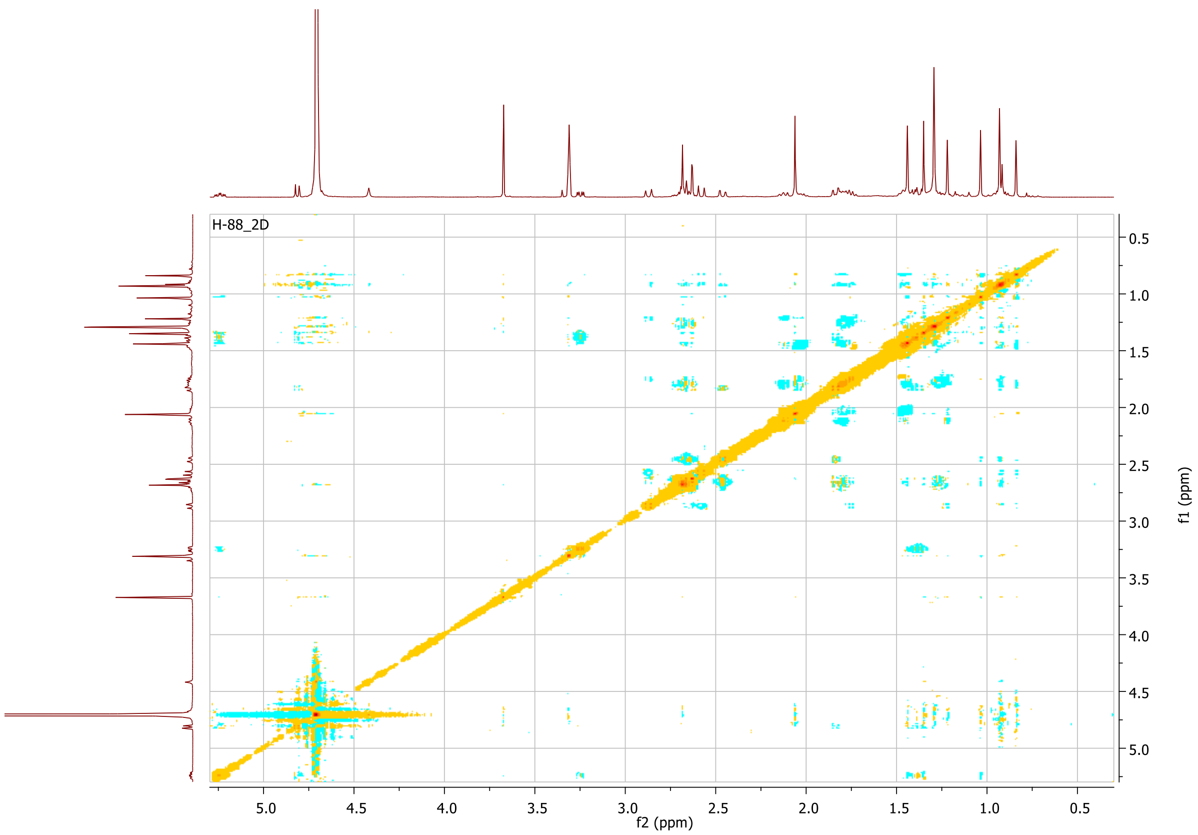

Fig. S27. NOESY spectrum of compound **4** (500 MHz, CD_3OD)

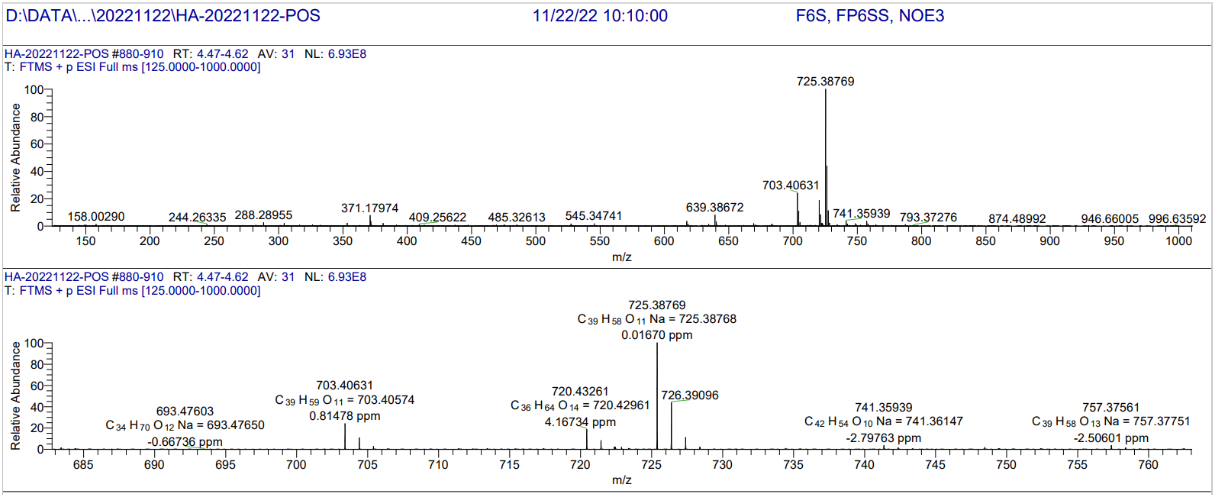

Fig. S28. HRESIMS spectrum of compound **4**

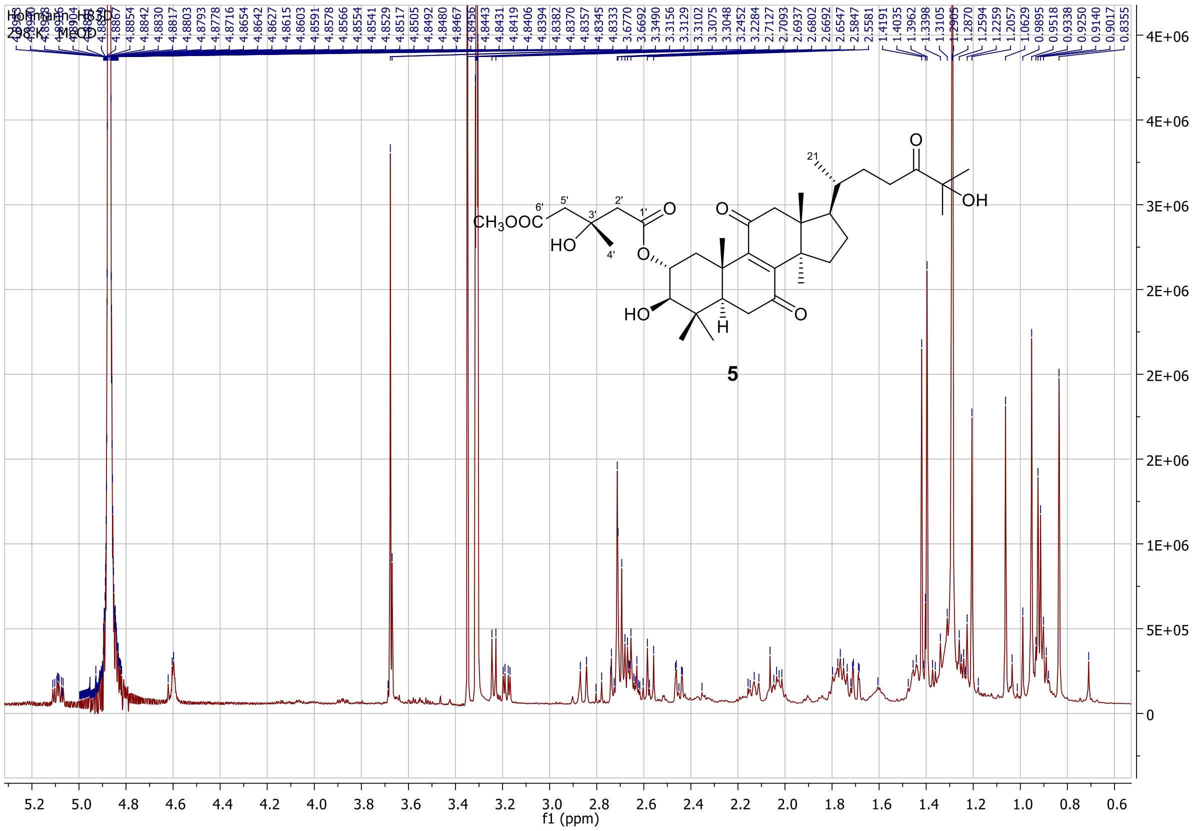

Fig. S29. ^{1}H NMR spectrum of compound **5** (500 MHz, CD_3OD)

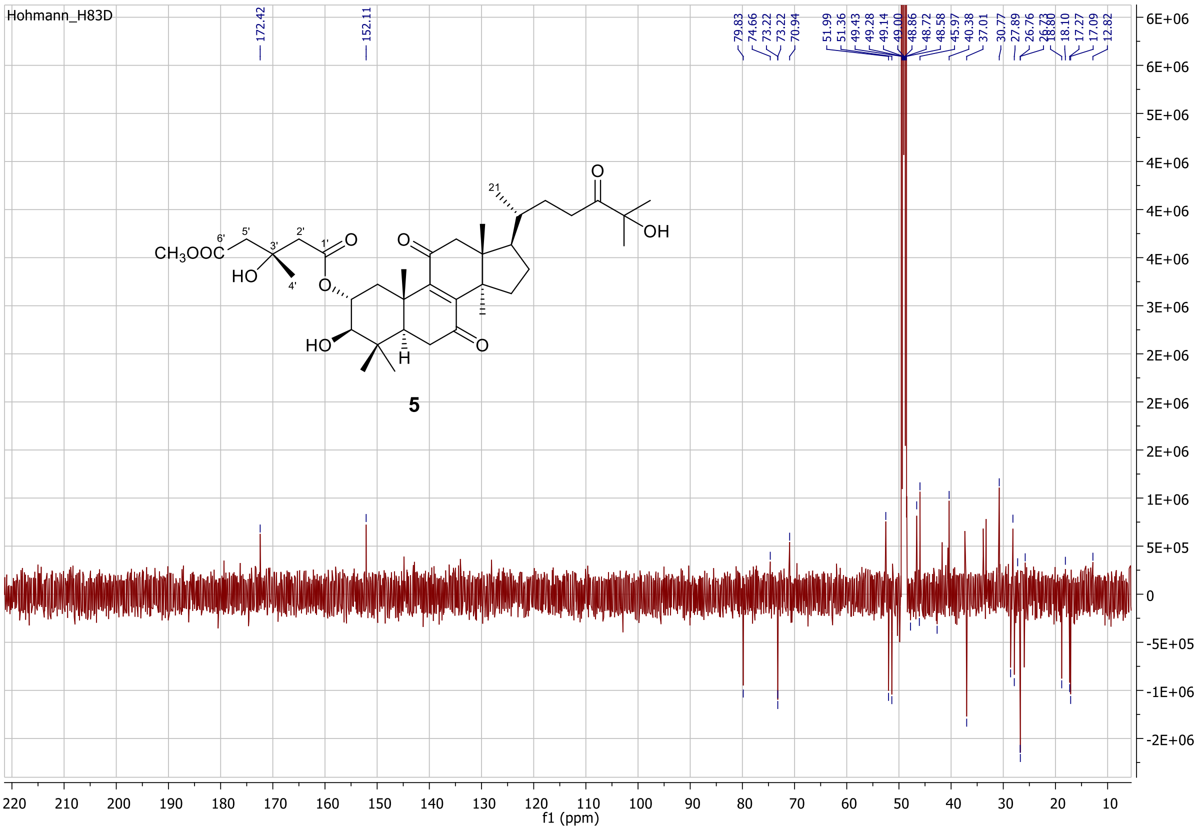

Fig. S30. ^{13}C-JMOD spectrum of compound **5** (150 MHz, CD_3OD)

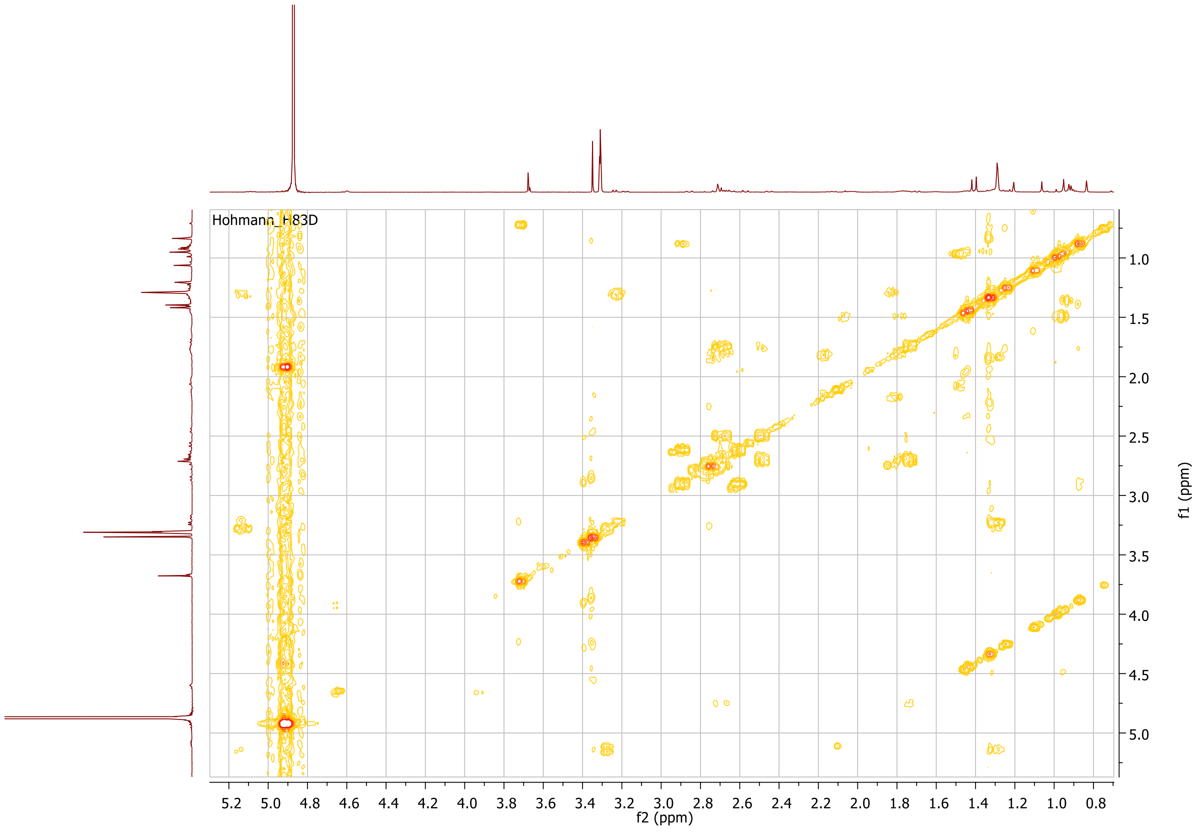

Fig. S31. ^{1}H-^{1}H COSY spectrum of compound **5** (600 MHz, CD_3OD)

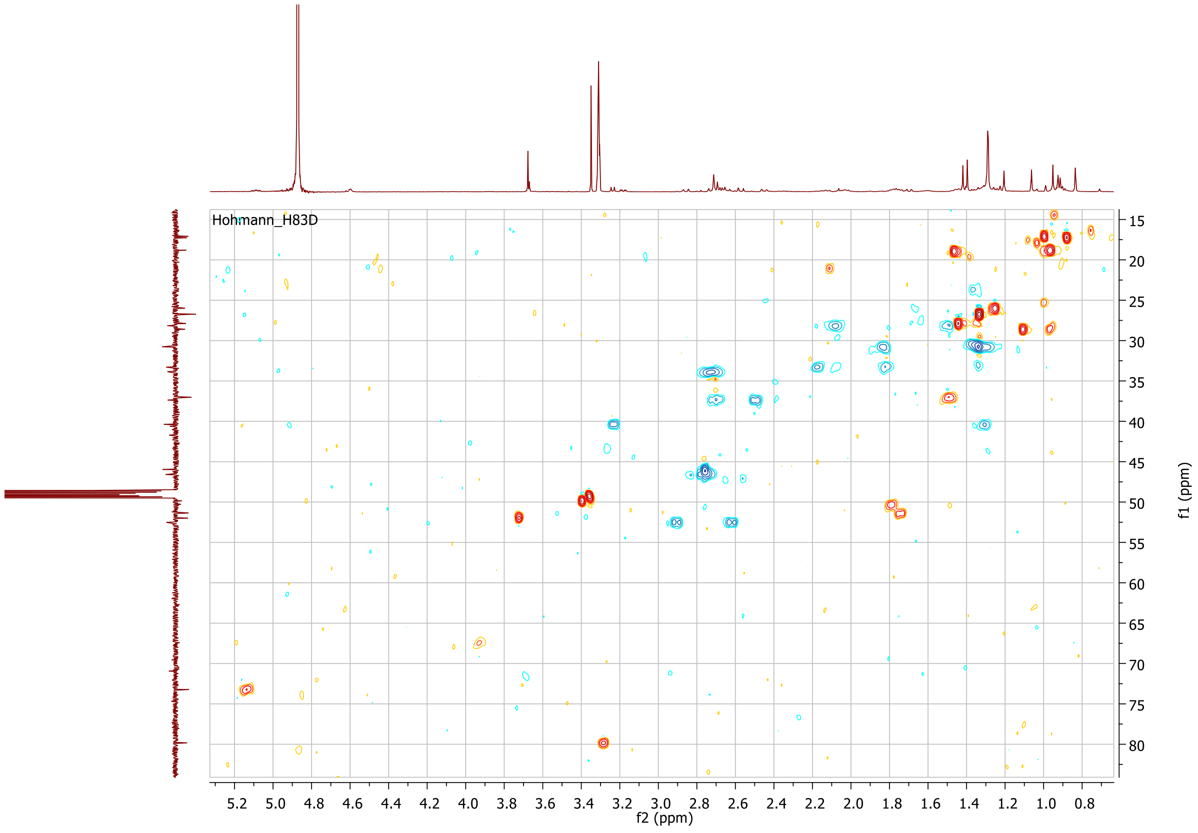

Fig. S32. HSQC spectrum of compound **5** (600/150 MHz, CD_3OD)

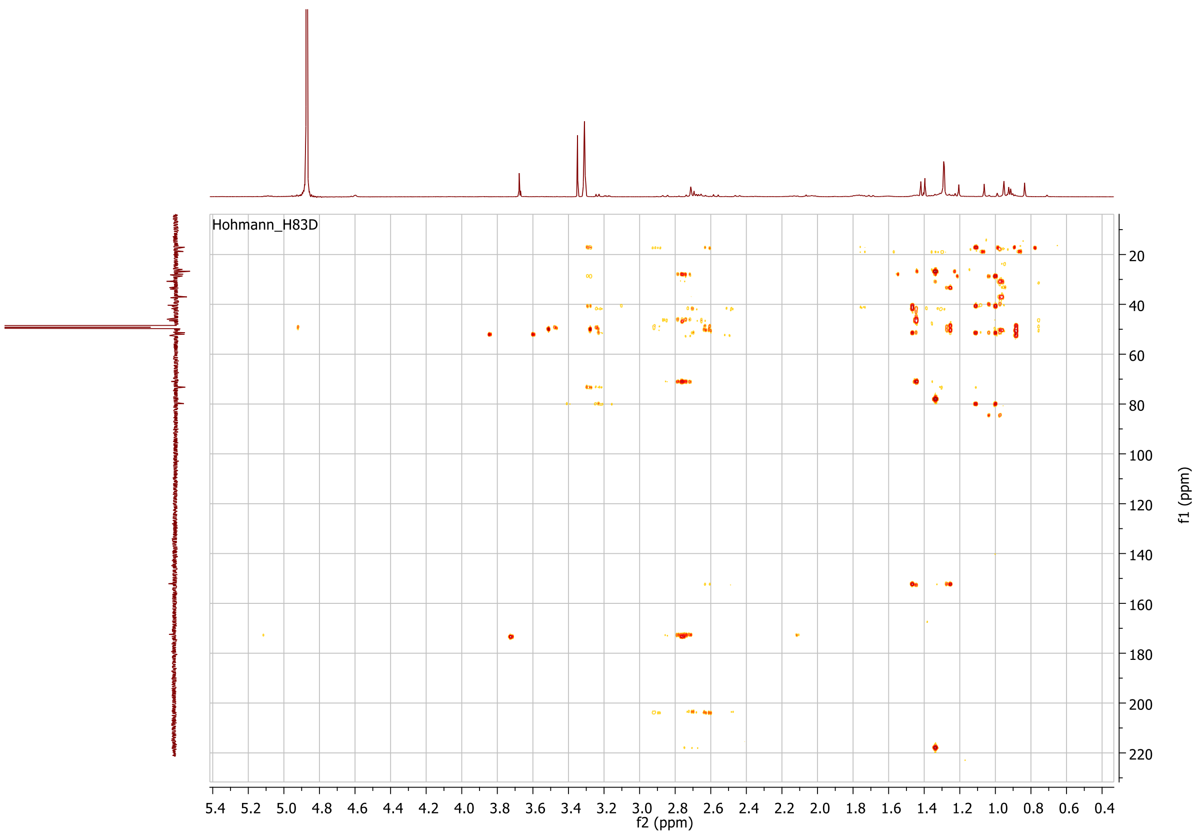

Fig. S33. HMBC spectrum of compound **5** (600/150 MHz, CD_3OD)

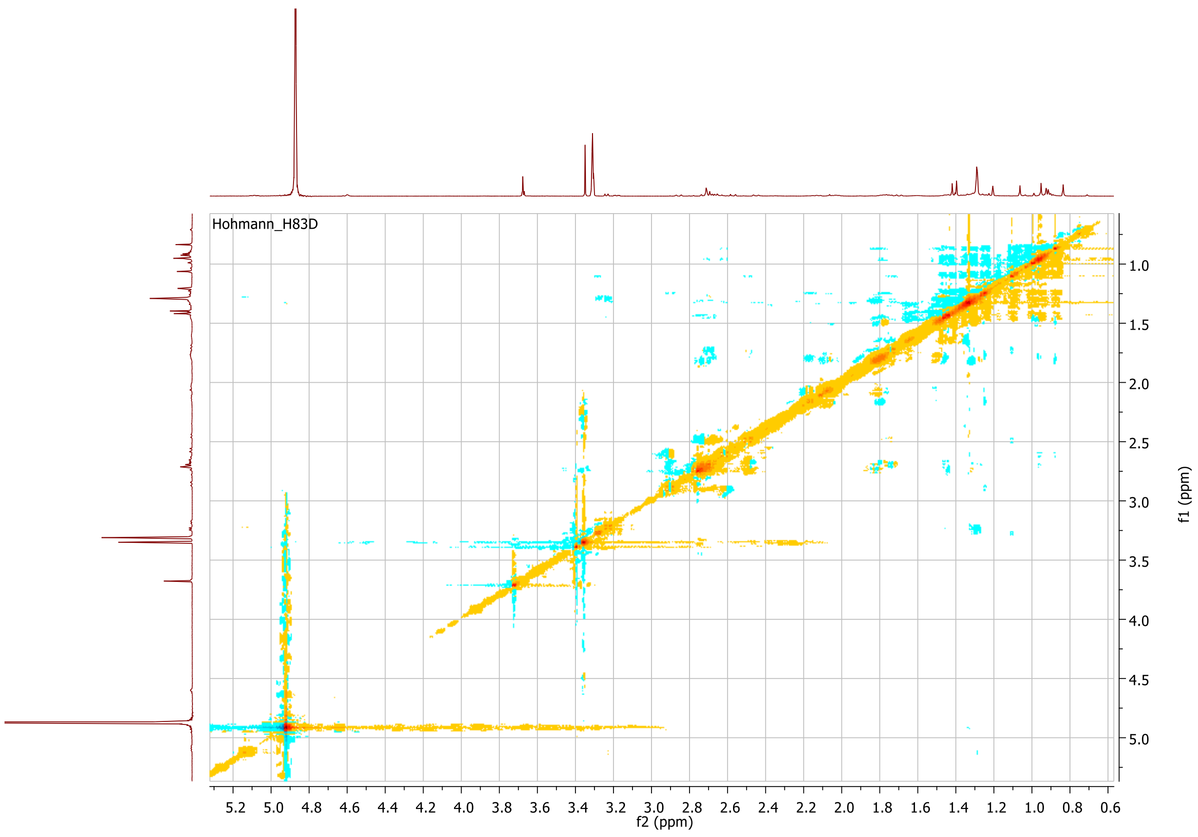

Fig. S34. NOESY spectrum of compound **5** (600 MHz, CD_3OD)

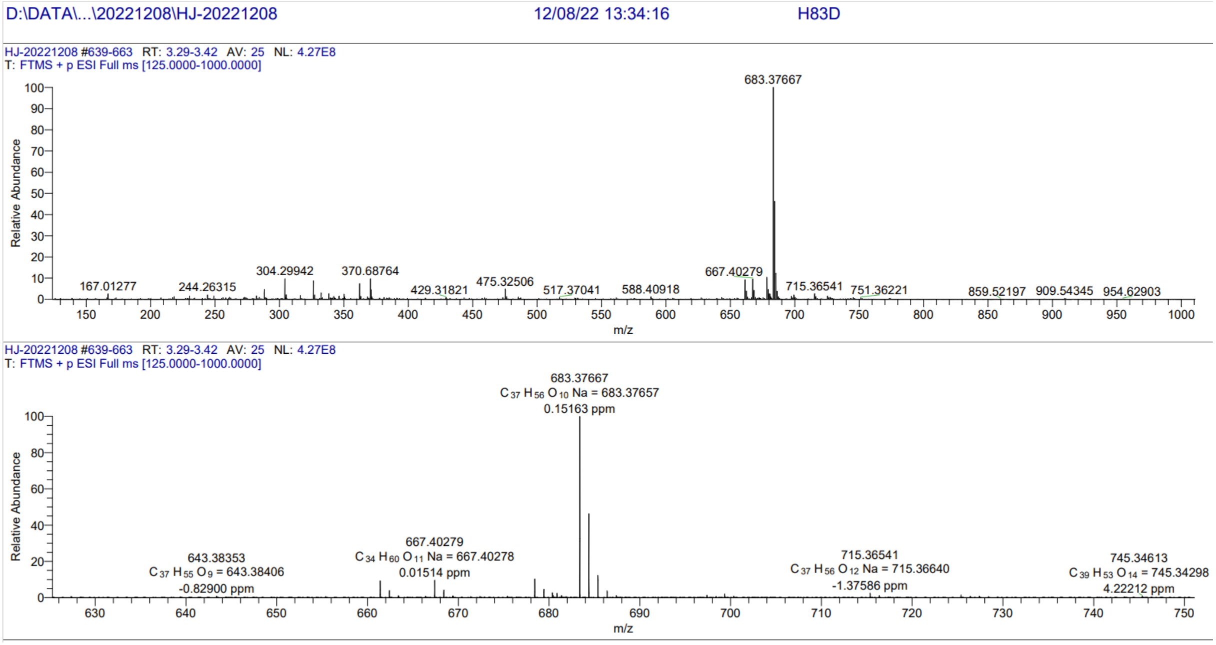

Fig. S35. HRESIMS spectrum of compound **5**

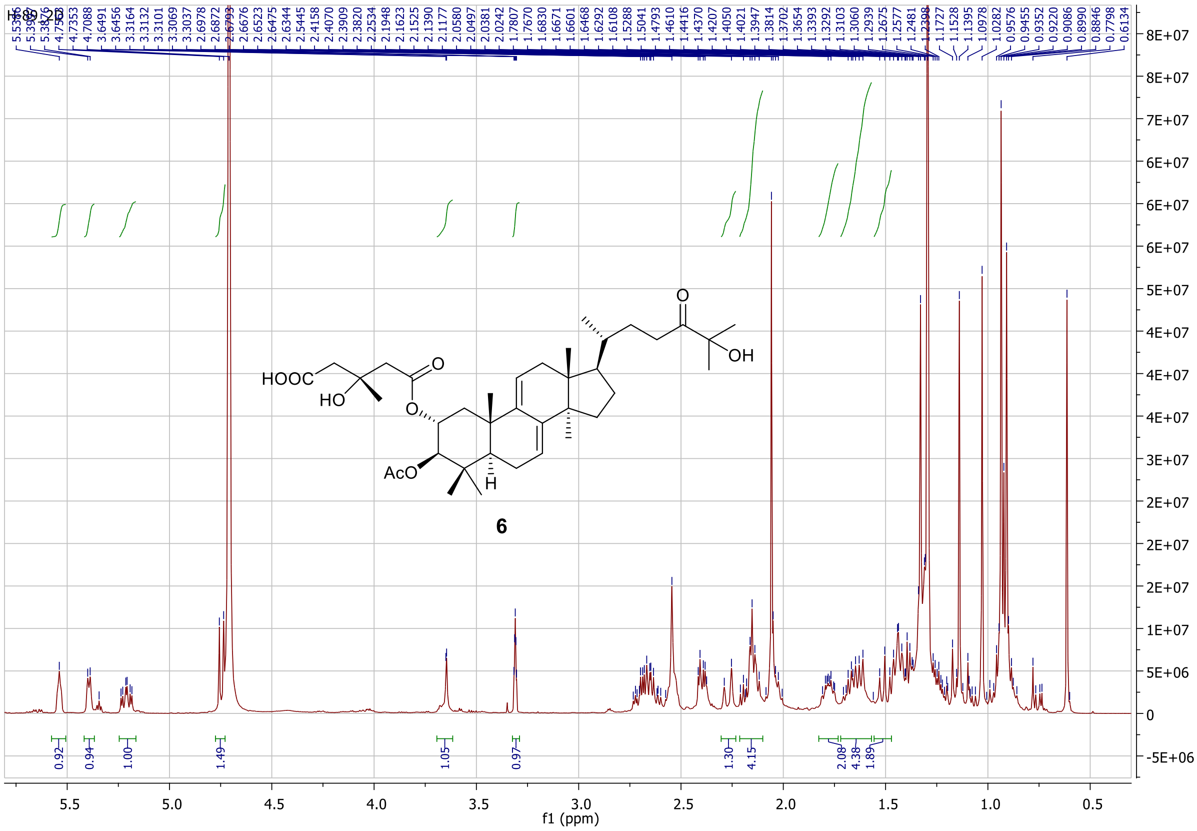

Fig. S36. ^{1}H NMR spectrum of compound **6** (500 MHz, CD_3OD)

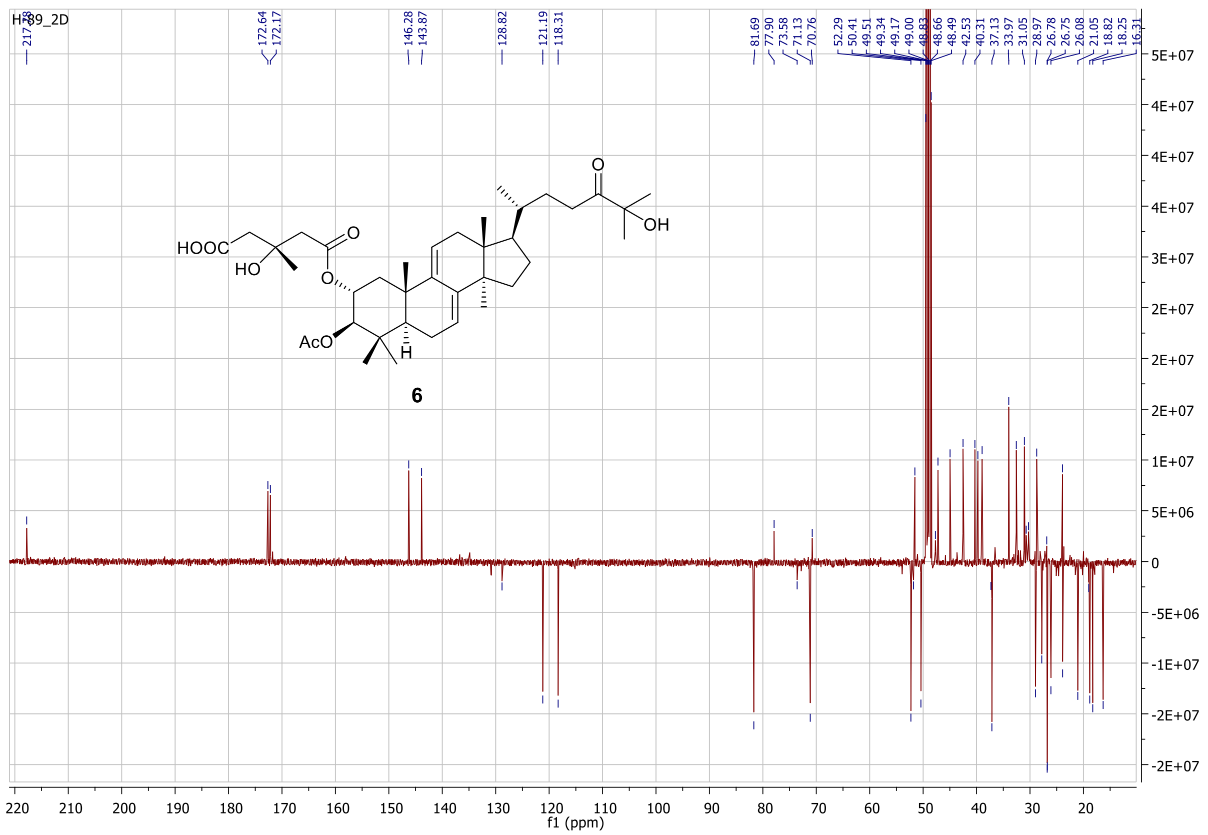

Fig. S37. ^{13}C-JMOD spectrum of compound **6** (125 MHz, CD_3OD)

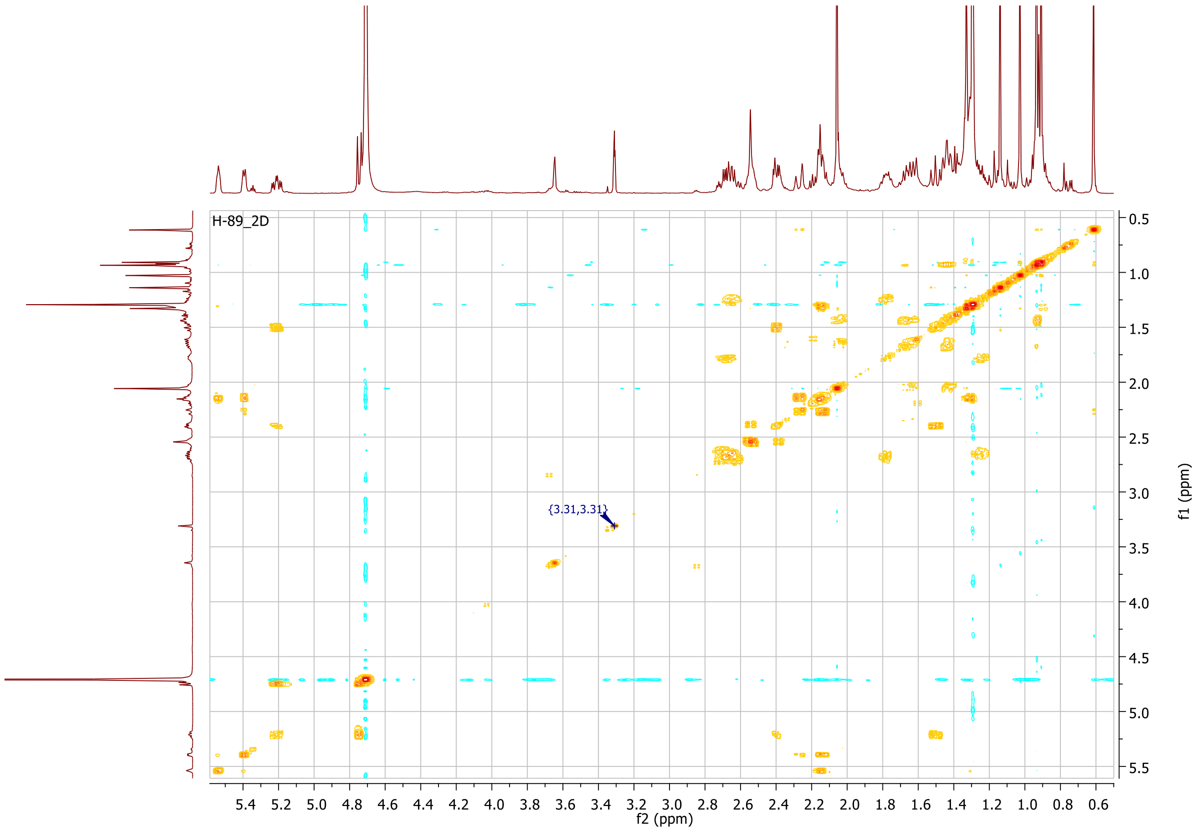

Fig. S38. ^{1}H-^{1}H COSY spectrum of compound **6** (500 MHz, CD_3OD)

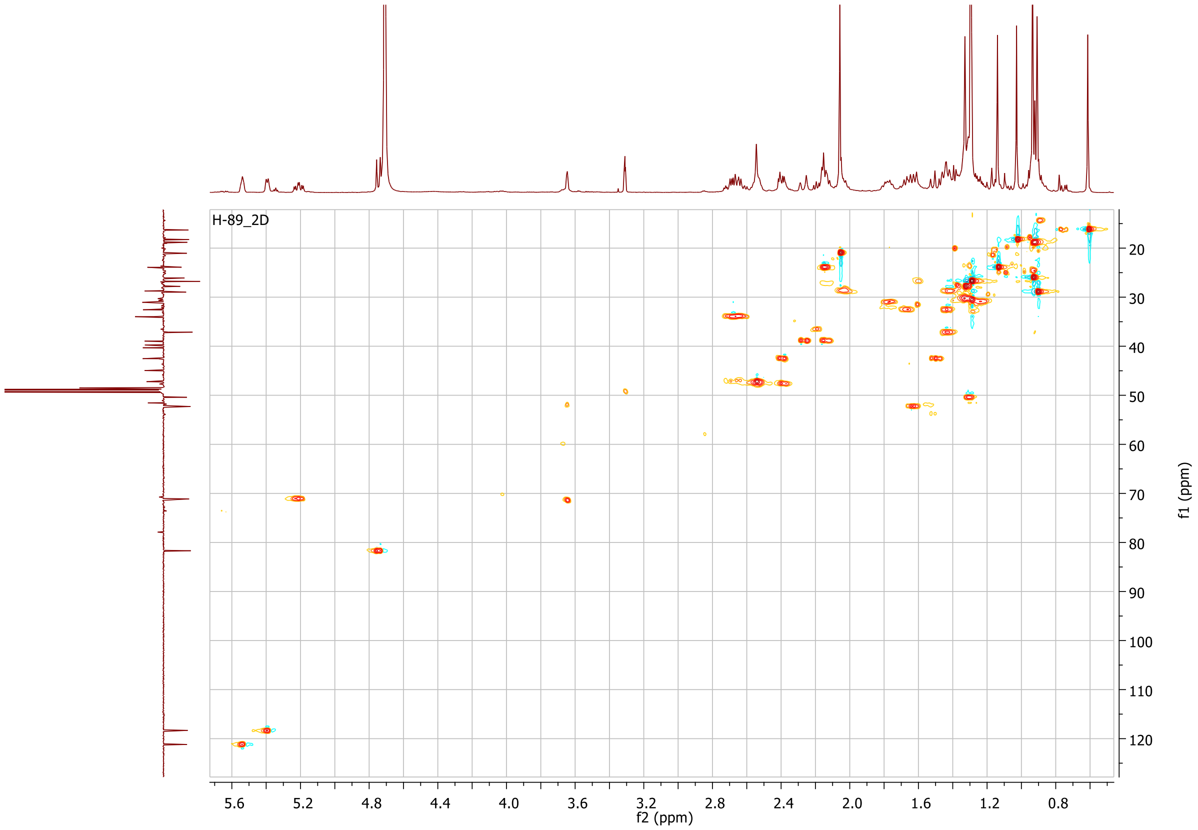

Fig. S39. HSQC spectrum of compound **6** (500/125 MHz, CD_3OD)

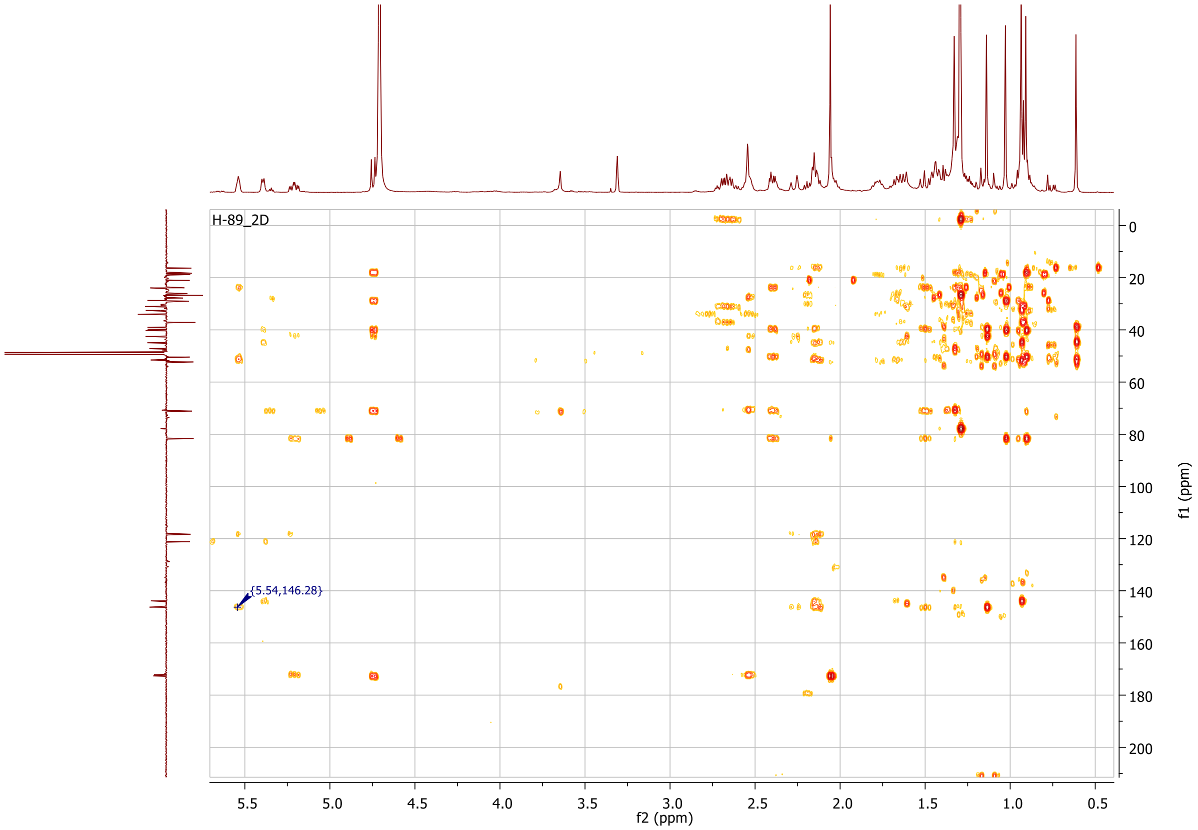

Fig. S40. HMBC spectrum of compound **6** (500/125 MHz, CD_3OD)

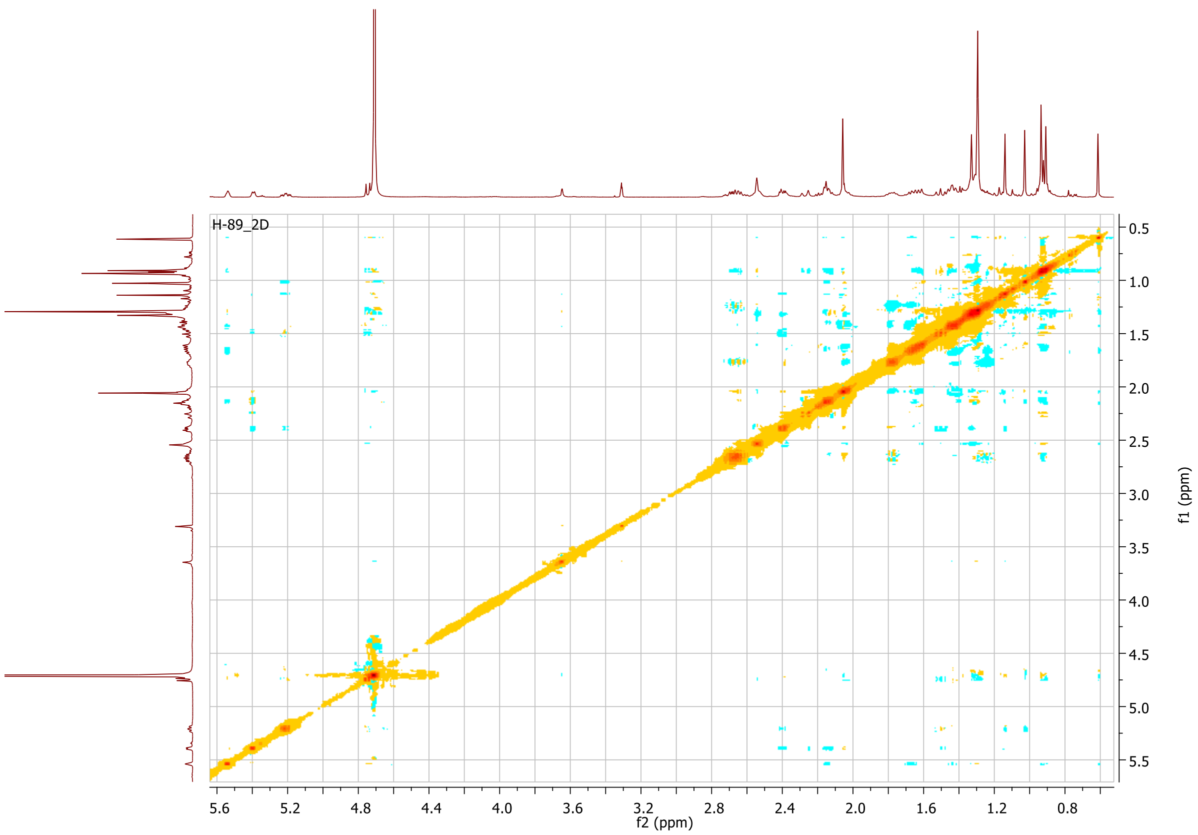

Fig. S41. NOESY spectrum of compound **6** (500 MHz, CD_3OD)

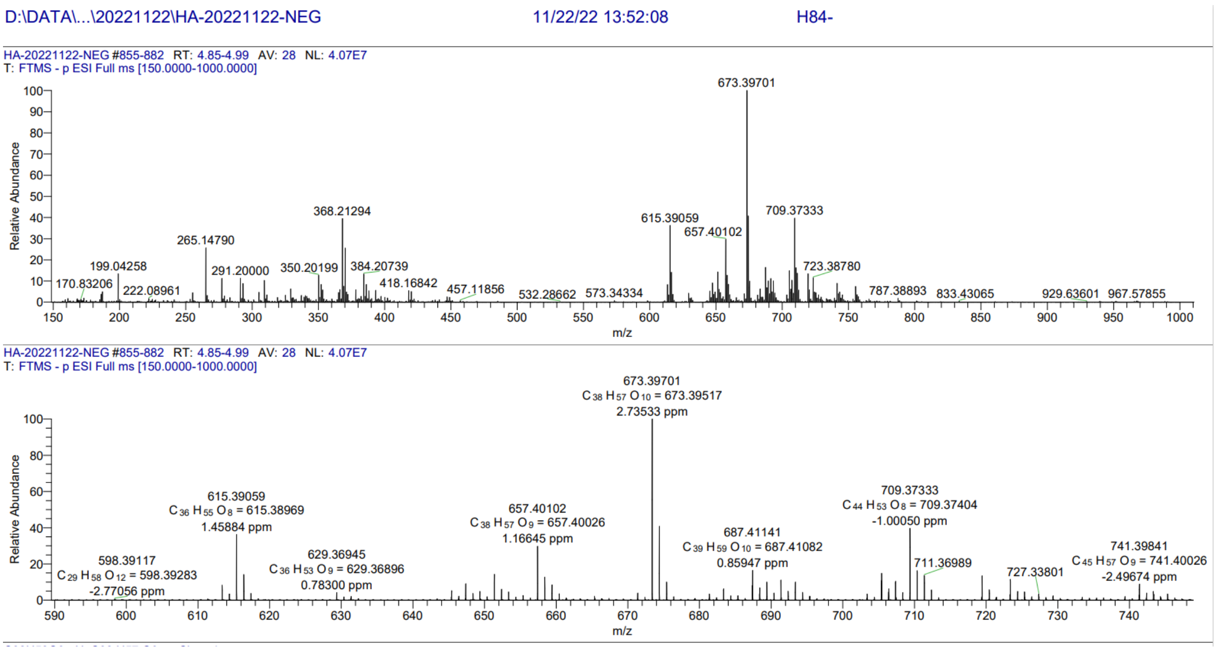

Fig. S42. HRESIMS spectrum of compound **6**

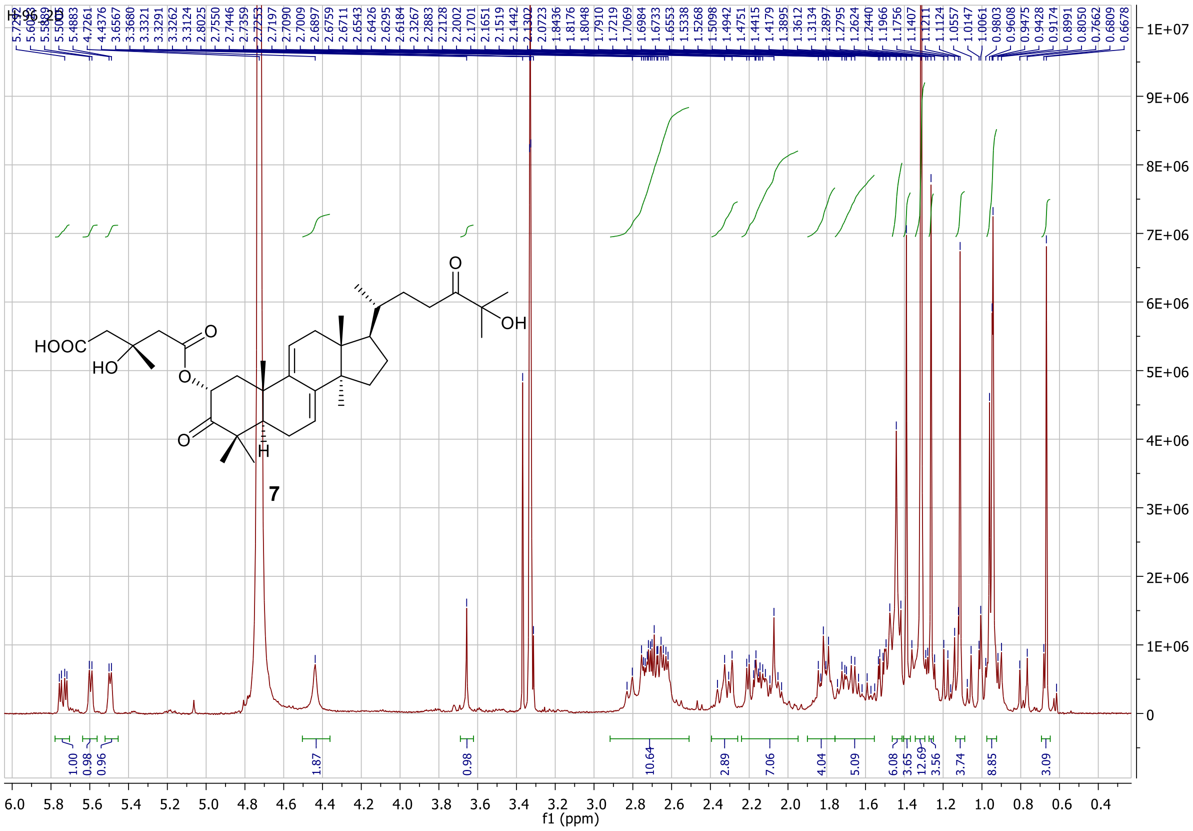

Fig. S43. ^{1}H NMR spectrum of compound **7** (500 MHz, CD_3OD)

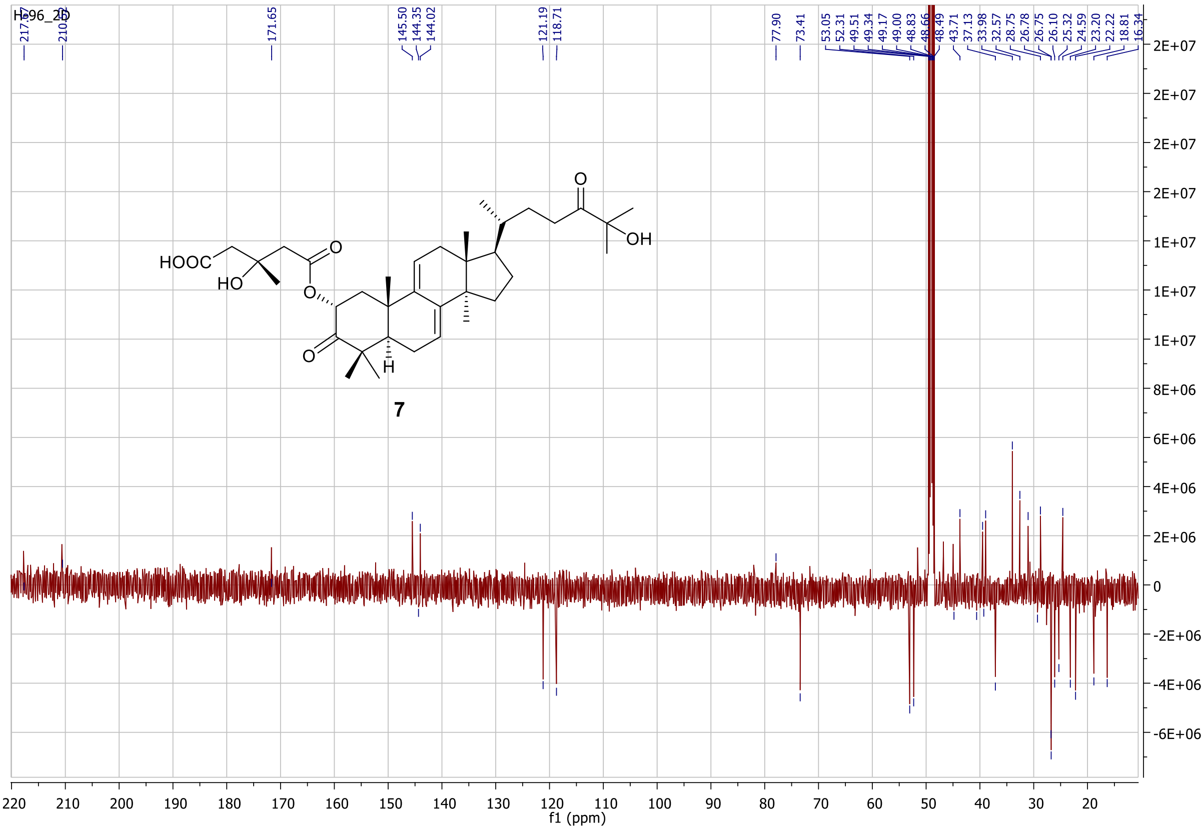

Fig. S44. ^{13}C-JMOD spectrum of compound **7** (125 MHz, CD_3OD)

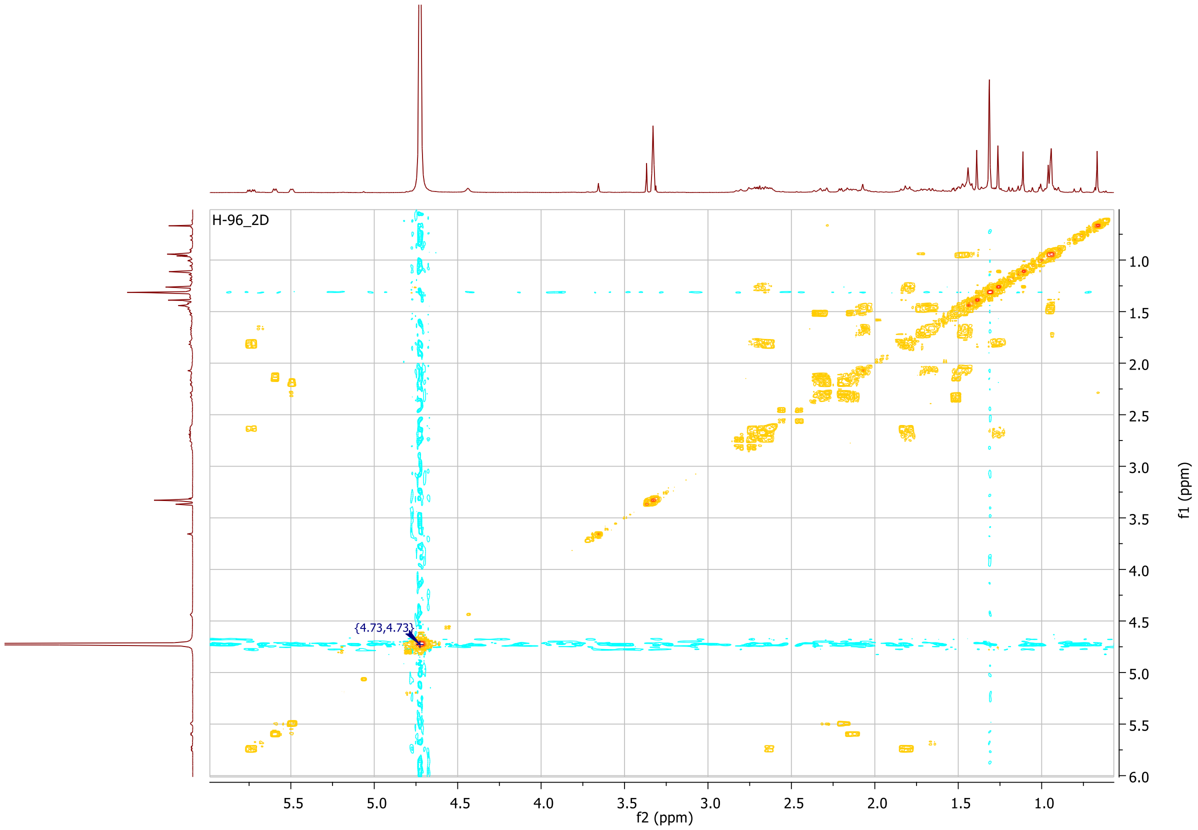

Fig. S45. ^{1}H-^{1}H COSY spectrum of compound **7** (500 MHz, CD_3OD)

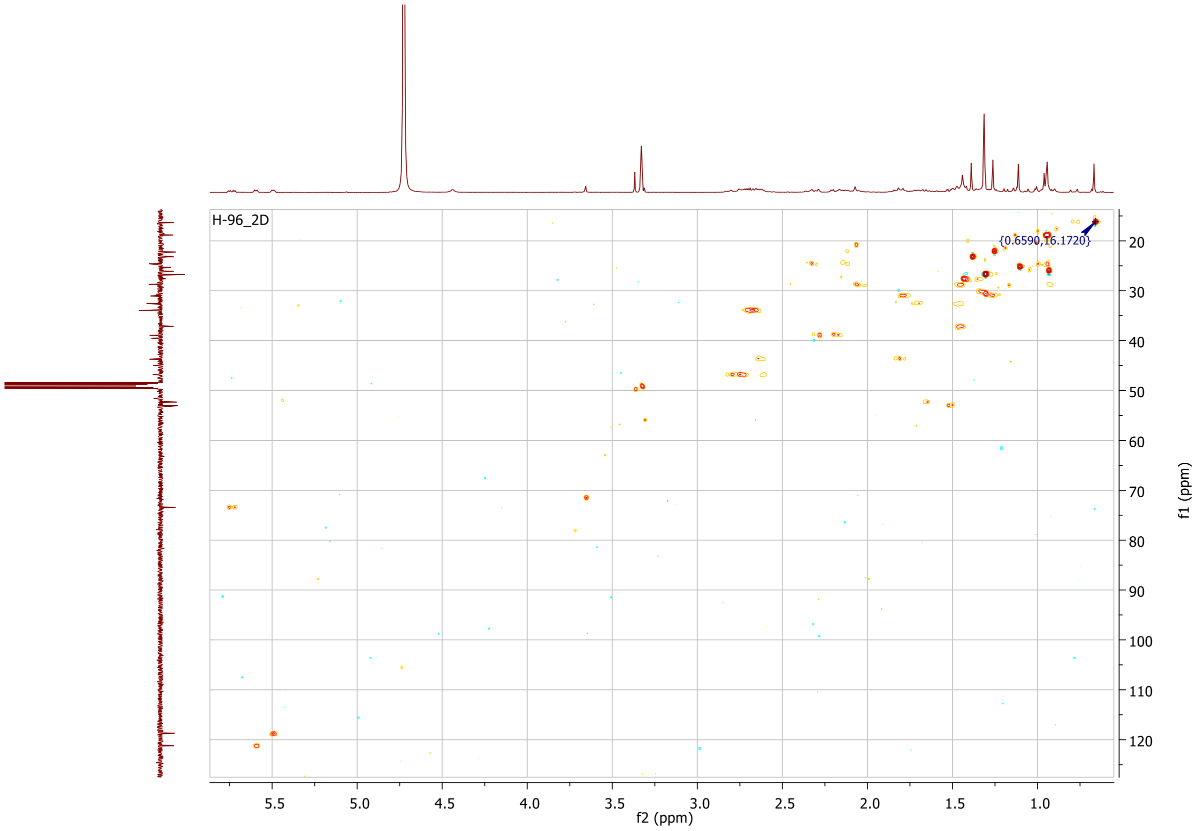

Fig. S46. HSQC spectrum of compound **7** (500/125 MHz, CD_3OD)

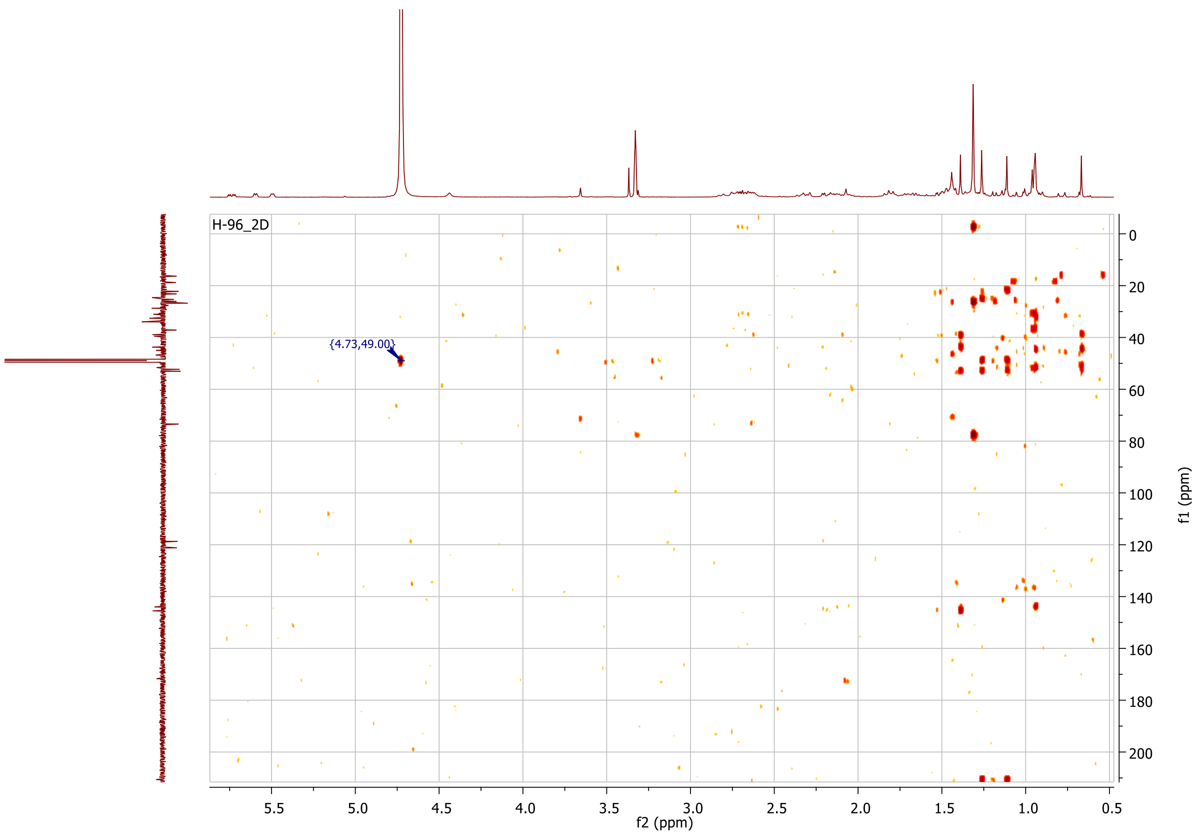

Fig. S47. HMBC spectrum of compound **7** (500/125 MHz, CD_3OD)

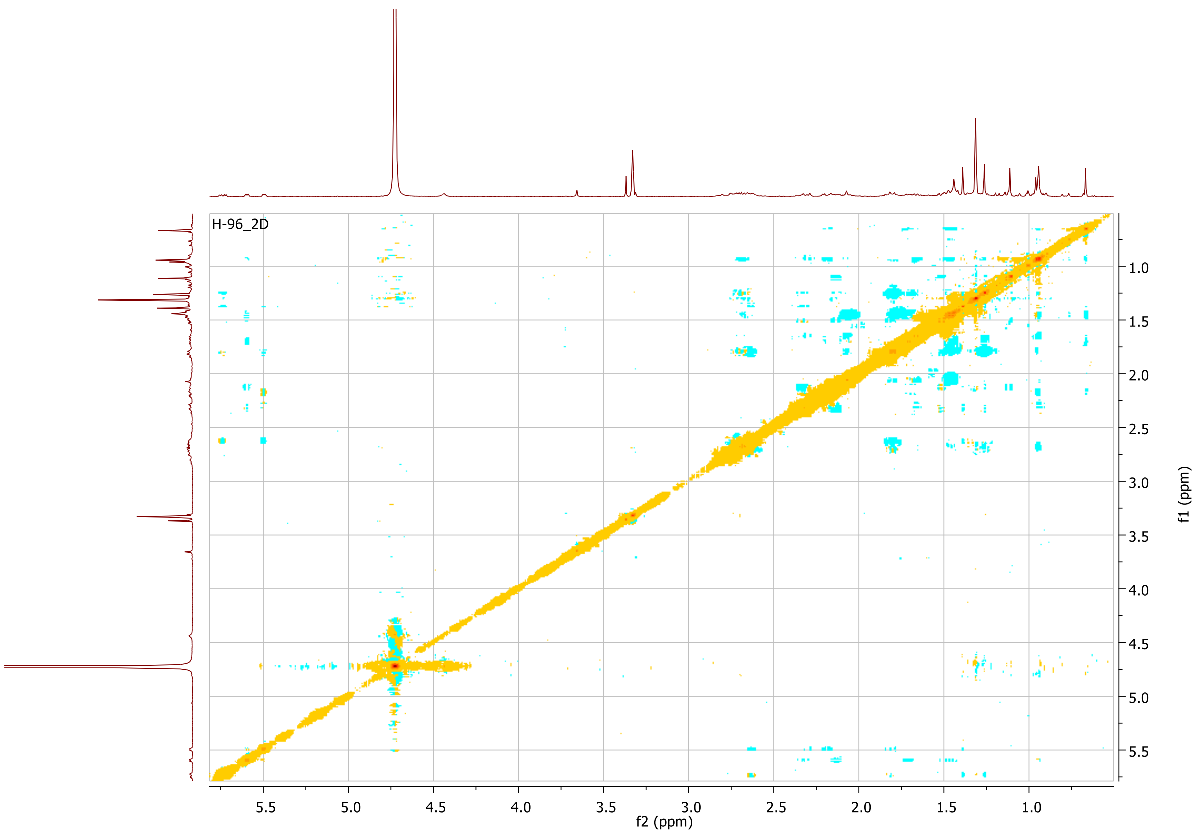

Fig. S48. NOESY spectrum of compound **7** (500 MHz, CD_3OD)

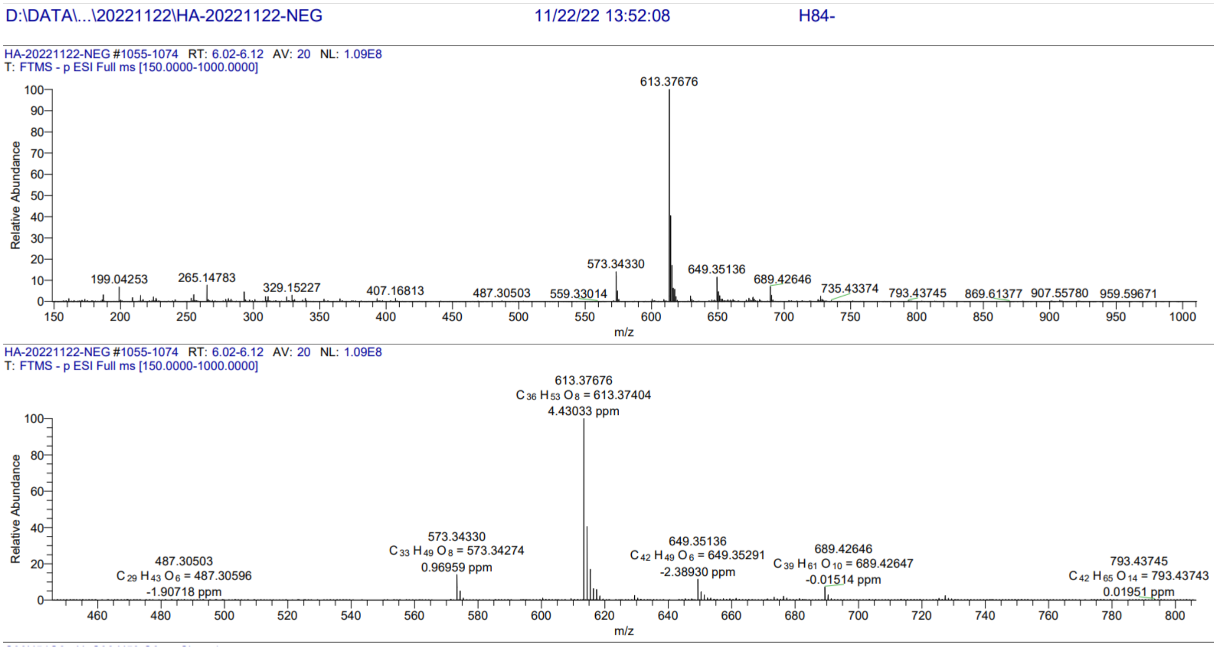

Fig. S49. HRESIMS spectrum of compound **7**

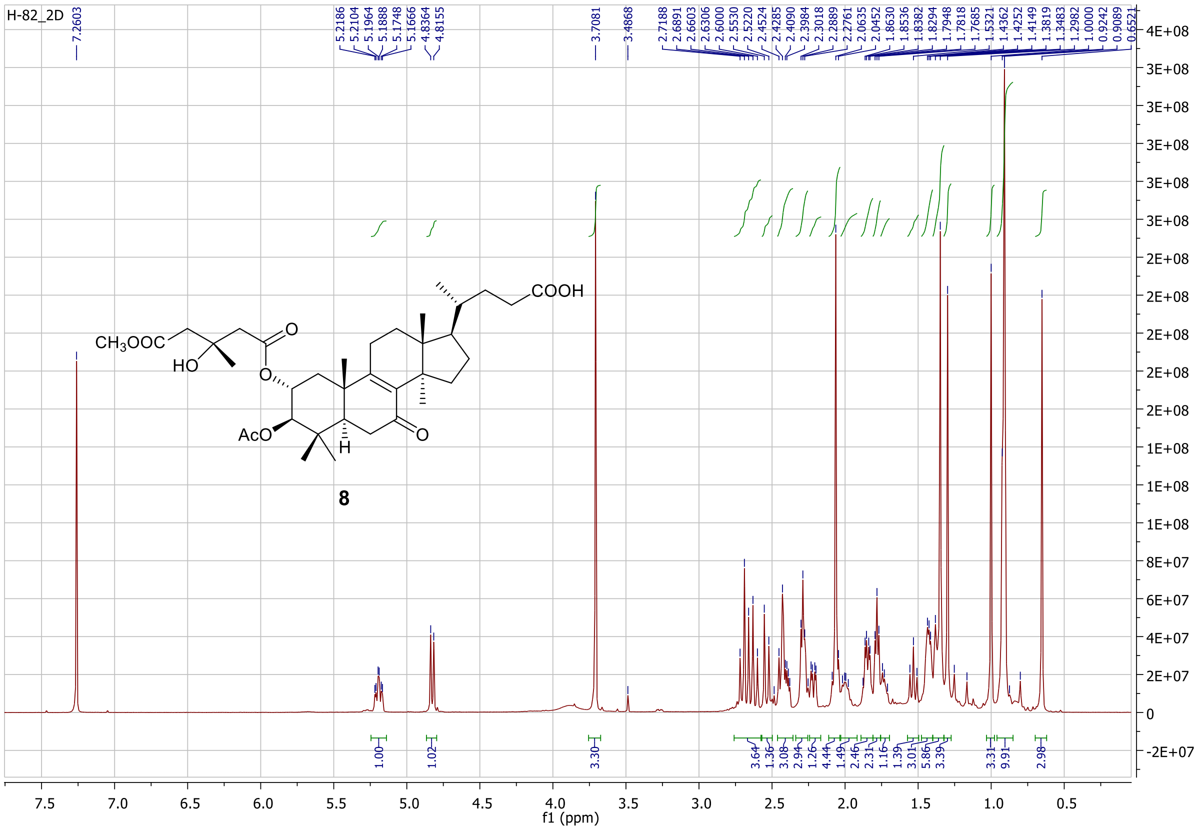

Fig. S50. ^1H NMR spectrum of compound **8** (500 MHz, $CDCl_3$)

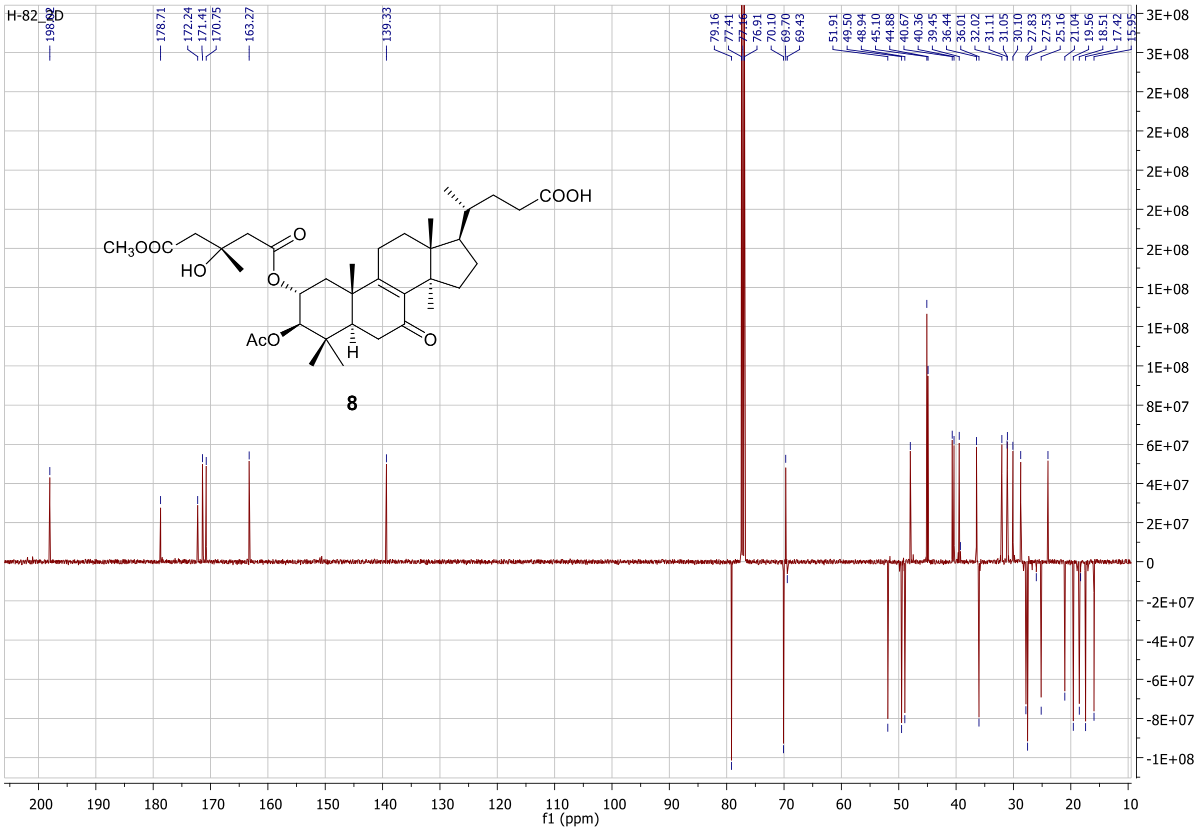

Fig. S51. ^{13}C-JMOD spectrum of compound **8** (125 MHz, $CDCl_3$)

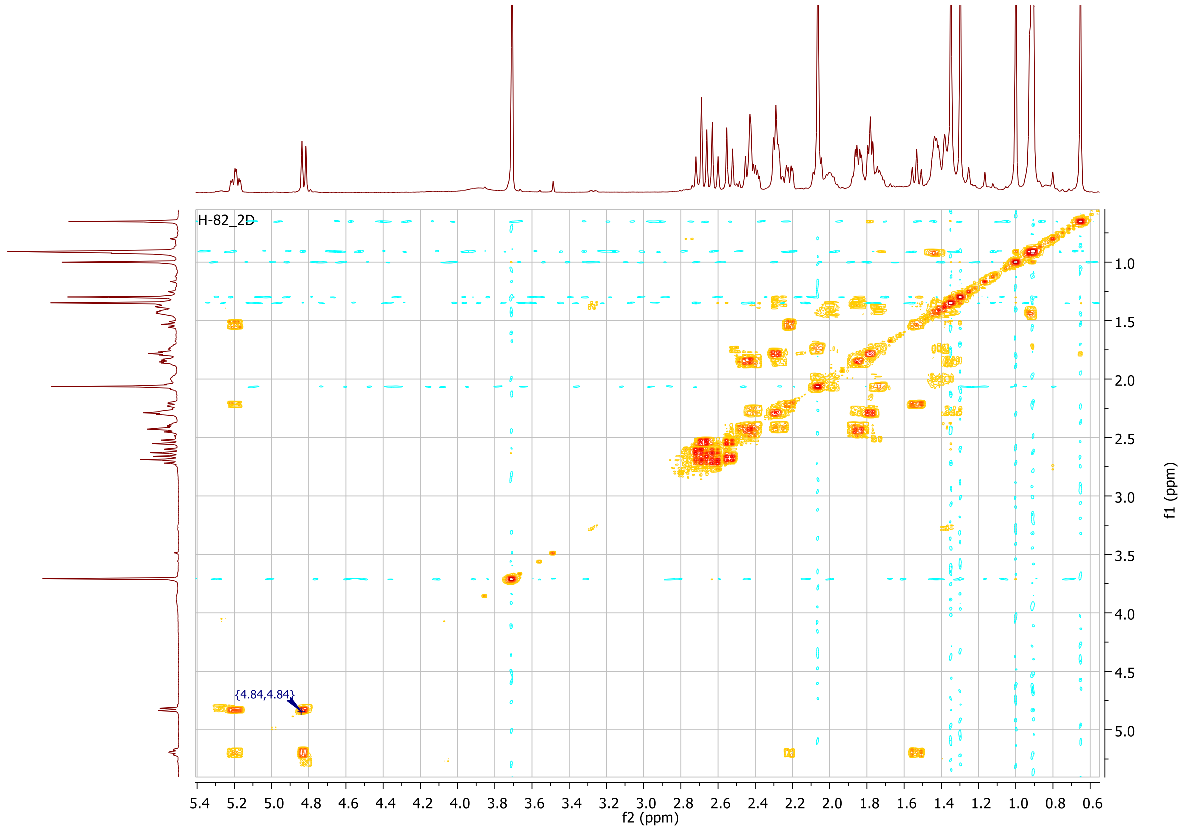

Fig. S52. ^{1}H-^{1}H COSY spectrum of compound **8** (500 MHz, $CDCl_3$)

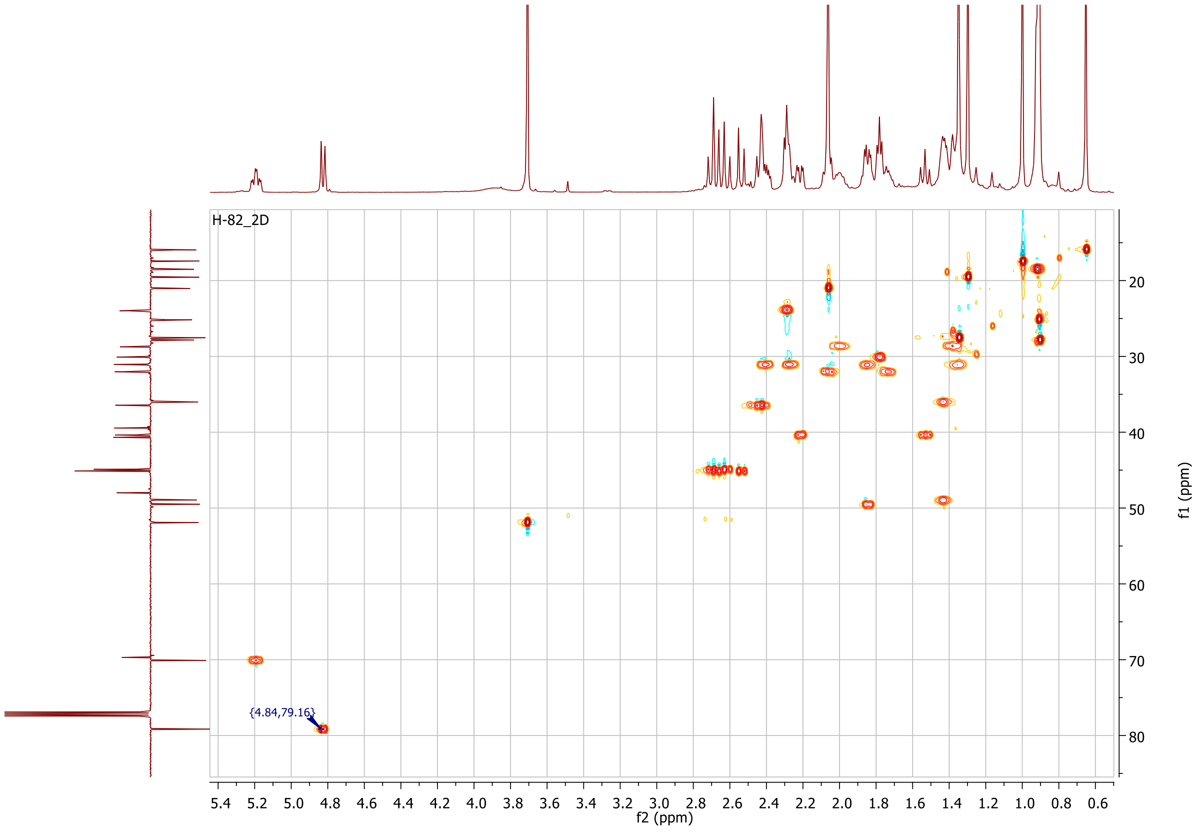

Fig. S53. HSQC spectrum of compound **8** (500/125 MHz, $CDCl_3$)

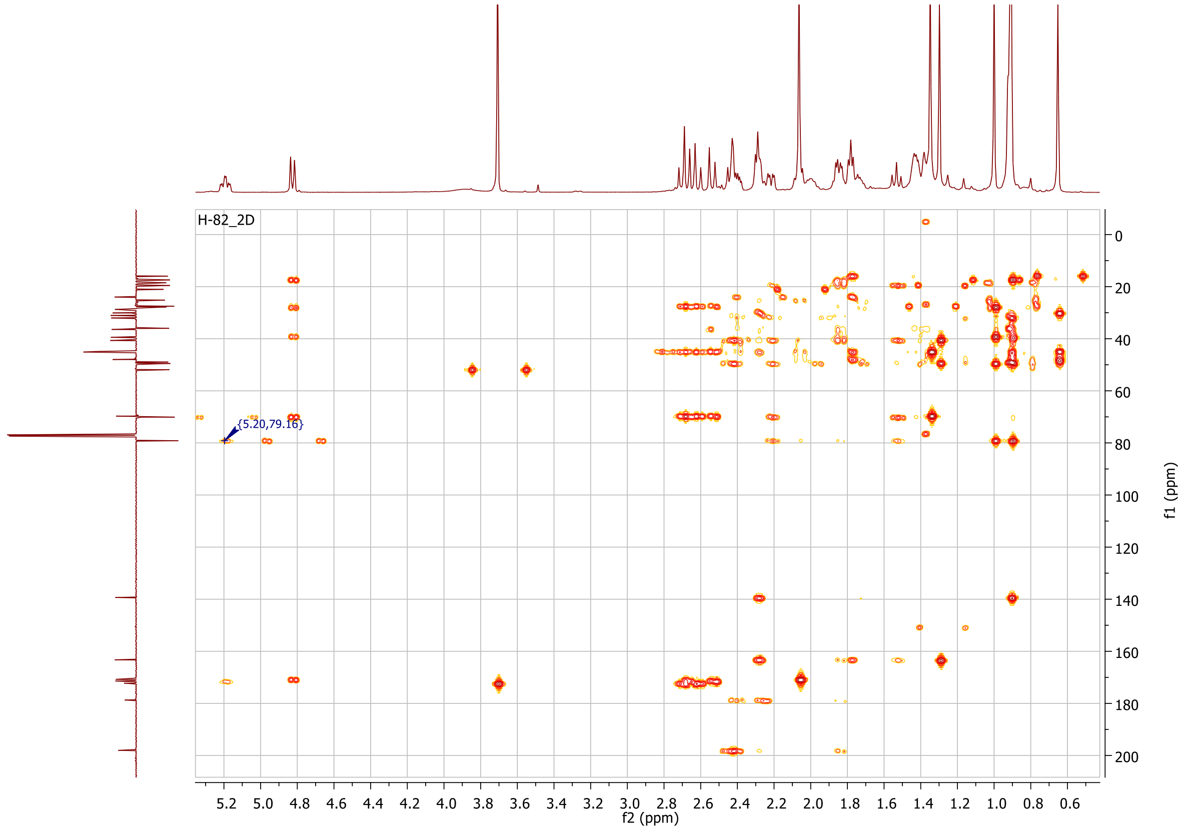

Fig. S54. HMBC spectrum of compound **8** (500/125 MHz, $CDCl_3$)

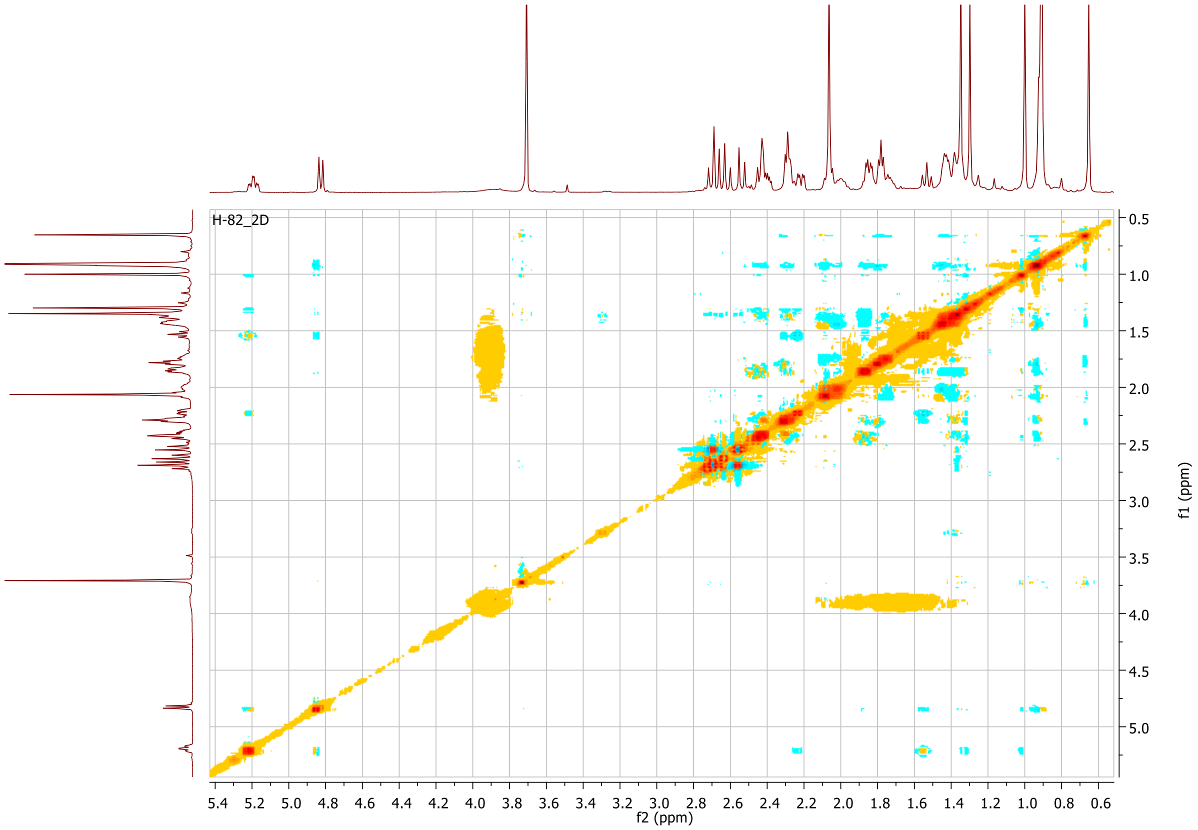

Fig. S55. NOESY spectrum of compound **8** (500 MHz, $CDCl_3$)

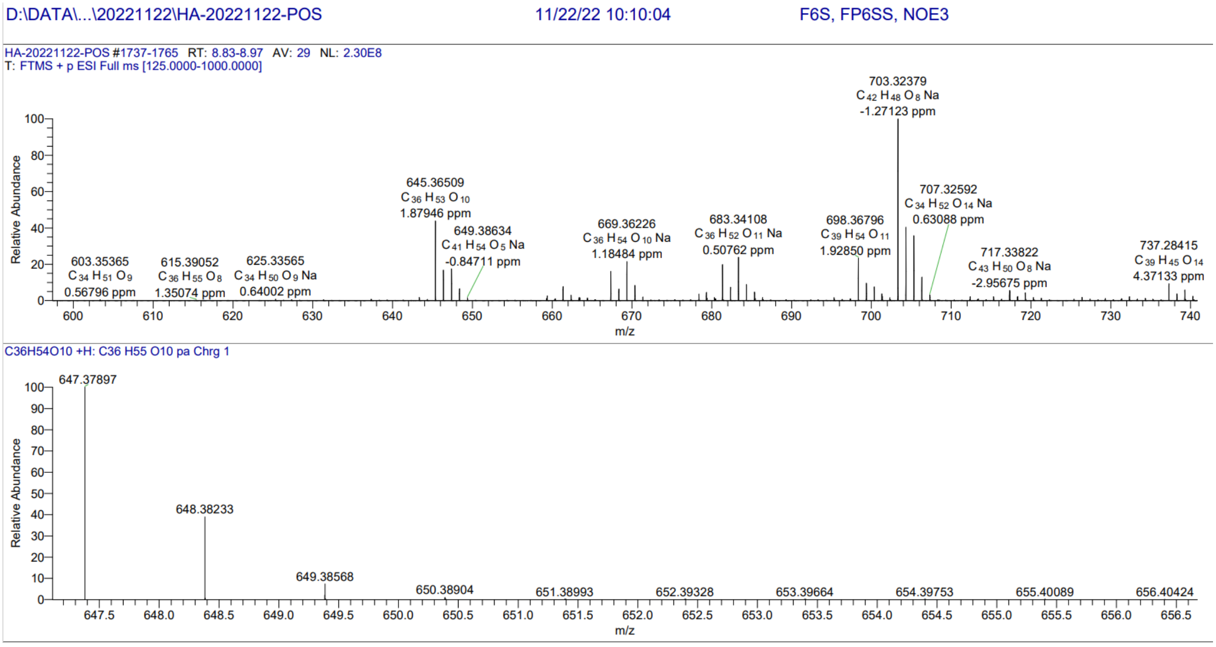

Fig. S56. HRESIMS spectrum of compound **8**

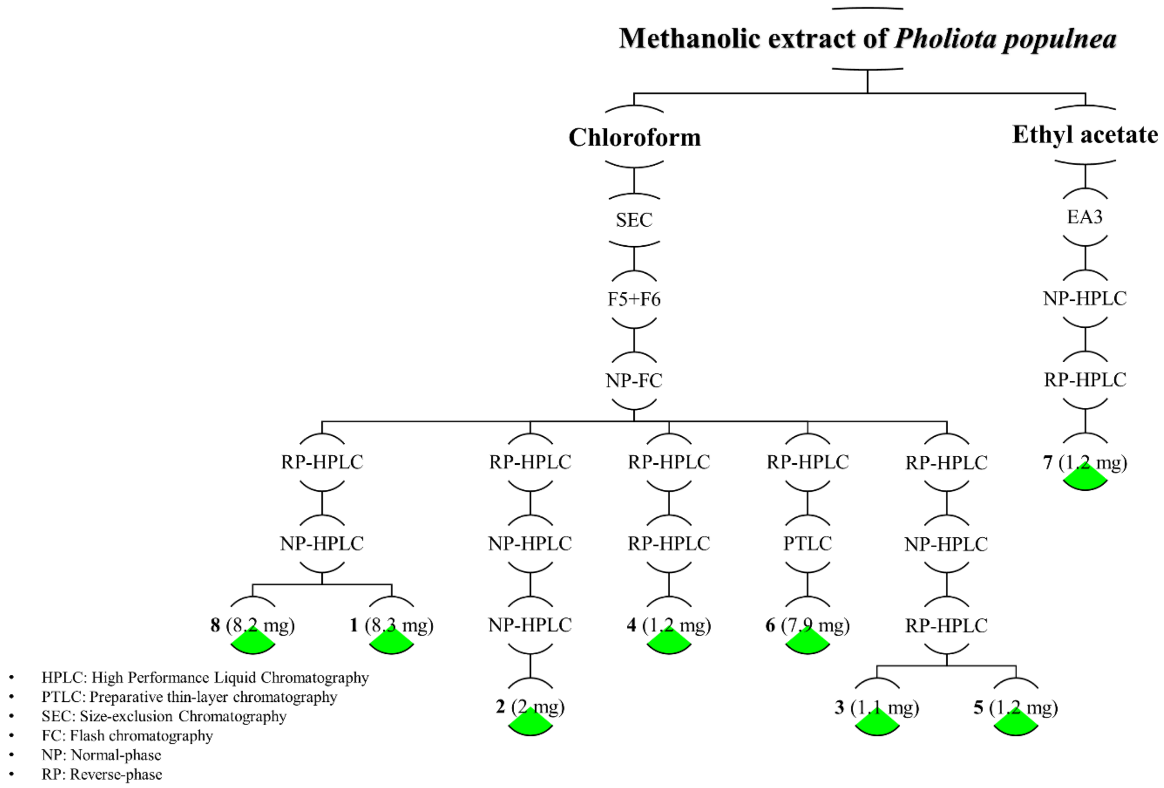

Fig. S57. Flow chart of the isolation of compounds **1–8**
